# Global incidence, risk factors, and temporal trends of nasal cancer: A population‐based analysis

**DOI:** 10.1002/cam4.70163

**Published:** 2025-04-17

**Authors:** Junjie Huang, Wing Sze Pang, Fung Yu Mak, Sze Chai Chan, Veeleah Lok, Lin Zhang, Xu Lin, Don Eliseo Lucero‐Prisno, Wanghong Xu, Zhi‐Jie Zheng, Edmar Elcarte, Mellissa Withers, Martin C. S. Wong, NCD Global Health Research Group, Association of Pacific Rim Universities (APRU)

**Affiliations:** ^1^ The Jockey Club School of Public Health and Primary Care, Faculty of Medicine Chinese University of Hong Kong Hong Kong SAR China; ^2^ Centre for Health Education and Health Promotion, Faculty of Medicine The Chinese University of Hong Kong Hong Kong SAR China; ^3^ Department of Global Public Health Karolinska Institute, Karolinska University Hospital Stockholm Sweden; ^4^ Suzhou Industrial Park Monash Research Institute of Science and Technology Suzhou China; ^5^ The School of Public Health and Preventive Medicine Monash University Clayton Victoria Australia; ^6^ Department of Thoracic Surgery, The First Affiliated Hospital, School of Medicine Zhejiang University Zhejiang Hangzhou China; ^7^ Department of Global Health and Development London School of Hygiene and Tropical Medicine London UK; ^8^ School of Public Health, Fudan University Shanghai China; ^9^ Department of Global Health School of Public Health, Peking University Beijing China; ^10^ University of the Philippines Manila Philippines; ^11^ Department of Population and Health Sciences Institute for Global Health, University of Southern California Los Angeles California USA

**Keywords:** incidence, nasal cancer, risk factors, temporal trend

## Abstract

**Background:**

Nasal cancer is a rare and fatal disease, with an incidence rate of <1 in 100,000, and a 5‐year survival rate of around 30%. The study aims to investigate the worldwide disease burden, associated risk factors, and temporal incidence patterns of nasal cancer.

**Methods:**

Data were obtained from multiple sources, including the Global Cancer Observatory, Cancer Incidence in Five Continents Plus, the Global Burden of Disease database, the World Bank, and the United Nations. The study utilized multivariable linear regression to investigate the relationship between risk factors and the incidence of nasal cancer by age for each country. Trend analysis was conducted using the joinpoint regression analysis program, and the average annual percentage change (AAPC) was calculated. The accuracy of trend estimations was assessed using the 95% confidence interval (CI). Additionally, the incidence of nasal cancer was examined by age and geographic location.

**Results:**

A total of 37,674 new cases were reported in 2020 (ASR 4.2 per 1,000,000). The highest ASRs were observed in South‐Eastern Asia (5.3) and Central and Eastern Europe (4.8). A number of risk factors were identified, such as higher HDI regions, higher prevalence of smoking, alcohol drinking, unhealthy dietary, and hypertension. In addition, physical inactivity was related to lower incidence. An overall decreasing trend was reported in the global population, but an increasing trend was discovered in males.

**Conclusions:**

The highest burden of nasal cancer was found in South‐Eastern Asia and Central and Eastern Europe, potentially due to regional genetic factors and pollution issues. Targeted interventions are need in high‐risk regions. Further studies are needed to investigate factors contributing to the increasing temporal trend of nasal cancer among the male population.

## 1 INTRODUCTION

Nasal cancer is typically fatal, the 5‐year survival rate is about 30%, and it significantly reduces the survivors' quality of life.^1^ However, the incidence rate of nasal cancer was low, with less than 1 in 100,000 people being diagnosed with,^2^, ^3^ especially in developed regions. Malignant epithelial tumors are found in over three‐quarters of all nose and paranasal sinuses malignant tumors, in which adenocarcinoma and squamous cell carcinoma are the most prevalent histologies.^4^

Previous studies have identified several risk factors associated with nasal cancer, including exposure to certain chemicals (including chromates and isopropyl alcohol) and occupational hazards, such as wood dust in the furniture industry and leather dust in the shoemaking industry.^5^, ^6^ Other possible chemical exposures involve formaldehyde, paint, lacquer, and glue.^7^ Cigarette smoking is also a significant risk factor, particularly for heavy or long‐term smokers, who are twice as likely to develop nasal cancer as nonsmokers.^8^

However, previous epidemiology studies of nasal cancer have limitations, such as being limited to western countries and relatively old data, the studies also fail to analyze the disease relating to metabolic risk factors and age groups, highlighting the need for a more comprehensive investigation. By utilizing data from international cancer registries and risk factor databases, the aim of this study is to provide a comprehensive and updated investigation of the worldwide disease burden, associated risk factors, and temporal incidence patterns of nasal cancer in demographic subgroups. This will further support the development of primary preventive policies and early diagnosis strategies.

## 2 METHODS

### 2.1 Data sources

We retrieved the 2020 incidence of nasal cancer in 185 nations from the Global Cancer Observatory (GLOBOCAN) database, a global online database that compiles data on the incidence and mortality of 26 forms of cancer.^9^ GLOBOCAN was developed in collaboration between the International Cancer Registry, population‐based cancer registries, the World Health Organization, and publicly accessible web data. Information from international or national cancer registries included in GLOBOCAN was used to calculate incidence‐to‐mortality ratios, trend analysis estimates, and adjacent country approximations.^10^ Using the decade‐long cancer incidence from 2003 to 2012 in 108 countries, the Cancer Incidence in Five Continents Plus (CI5 Plus) database was used to extract the incidence of nasal cancer by cancer site.^11^ The CI5 Plus database, used for time trend studies and interpreting differences over time, provides the most recent and historical incidence information broken down by year, population subgroup, and geographic location. We used ICD‐10 C30 to identify malignant neoplasm of nasal cavity to ensure the consistency of different registries. The GLOBOCAN and CI5 Plus are comprehensive compilation of reliable cancer registries at global, regional, and national scales, encompassing a significant proportion of the world's population, offering verified incidence data by confirming the occurrence of each newly documented cancer case within a specified timeframe. The risk factors analysis was conducted using the country‐specific information from the global burden of disease (GBD) database, which includes the prevalence of obesity, hypertension, diabetes, lipid disorders, unhealthy diet, alcohol consumption, and smoking.^12^ The GBD database measures health loss brought on by specific illnesses, accidents, and risk factors in order to improve healthcare systems and achieve health equity. Data on the early mortality and impairment of more than 350 diseases and traumas were obtained and reviewed by over 7000 researchers from 156 countries and regions. For each country, the human development index (HDI) and gross domestic product (GDP) per capita were provided by the United Nations (UN) and the World Bank, respectively.^13^, ^14^

### 2.2 Statistical analysis

The incidence of nasal cancer and several risk factors (HDI, GDP per capita, lifestyle, and metabolic risk factors by sex and age) were examined using multivariable linear regression analysis. The matching Beta coefficients (β) and 95% confidence intervals (CI) for the regression analysis were calculated. β estimates measure as a unit increment of the change in an outcome variable (ASR) to the change in a predictor variable (risk factor). Statistical significance was defined as a *p* value of 0.05 or less, and all CI are displayed for 95% of the values.

Trend analysis was performed using the Joinpoint regression analysis tool. The SEER Program of the National Cancer Institute of the United States developed the software. It was utilized to examine the temporal pattern of nasal cancer incidence by location and country using the average annual percentage change (AAPC).^15^ The study adhered to established procedures in cancer epidemiology research by utilizing the most recent 10‐year data for calculations. In order to produce the necessary standard errors, incidence data were log‐transformed, and the 95% CI were then calculated. A positive AAPC indicates an increasing trend, while a negative AAPC indicates a decreasing trend over time. In order to examine the precision of trend estimation, a 95% CI was used. For instance, a range that passes 0 denotes a persistent trend without a clear rise or fall. In addition, the incidence trend of nasal cancer by age (total population: 0–85+, young population: 15–49; old population: 50–74), gender (male and female), and geographic location (Asia, Oceania, Americas, Europe, Africa) are also assessed.

## 3 RESULTS

### 3.1 Nasal cancer incidence in 2020

The total number of new cases reported was 37,674 (ASR: 4.2 per 1,000,000) in 2020 (Table S1). South‐Eastern Asia (5.3), Central and Eastern Europe (4.8), Western Europe (4.7), South‐Central Asia (4.5), and Northern Africa (4.3) were the regions reported the highest ASRs; while Central America & Caribbean (2.8), Western Asia (3.0), Southern Europe (3.5), Northern America (3.6), and Sub‐Saharan Africa (3.7) were the regions reported the lowest ASRs. As for the country, Cabo Verde (17.1), North Macedonia (14.5), Côte d'Ivoire (13.3), Gabon (10.6), and France, La Réunion (9.9) were found to have the highest ASRs. The lowest ASRs were found in Bahrain (0.57), Eswatini (0.68), Tajikistan (0.84), El Salvador (0.90), and Guatemala (1.1).

### 3.2 Nasal cancer incidence by subgroup in 2020

**FIGURE 1:**
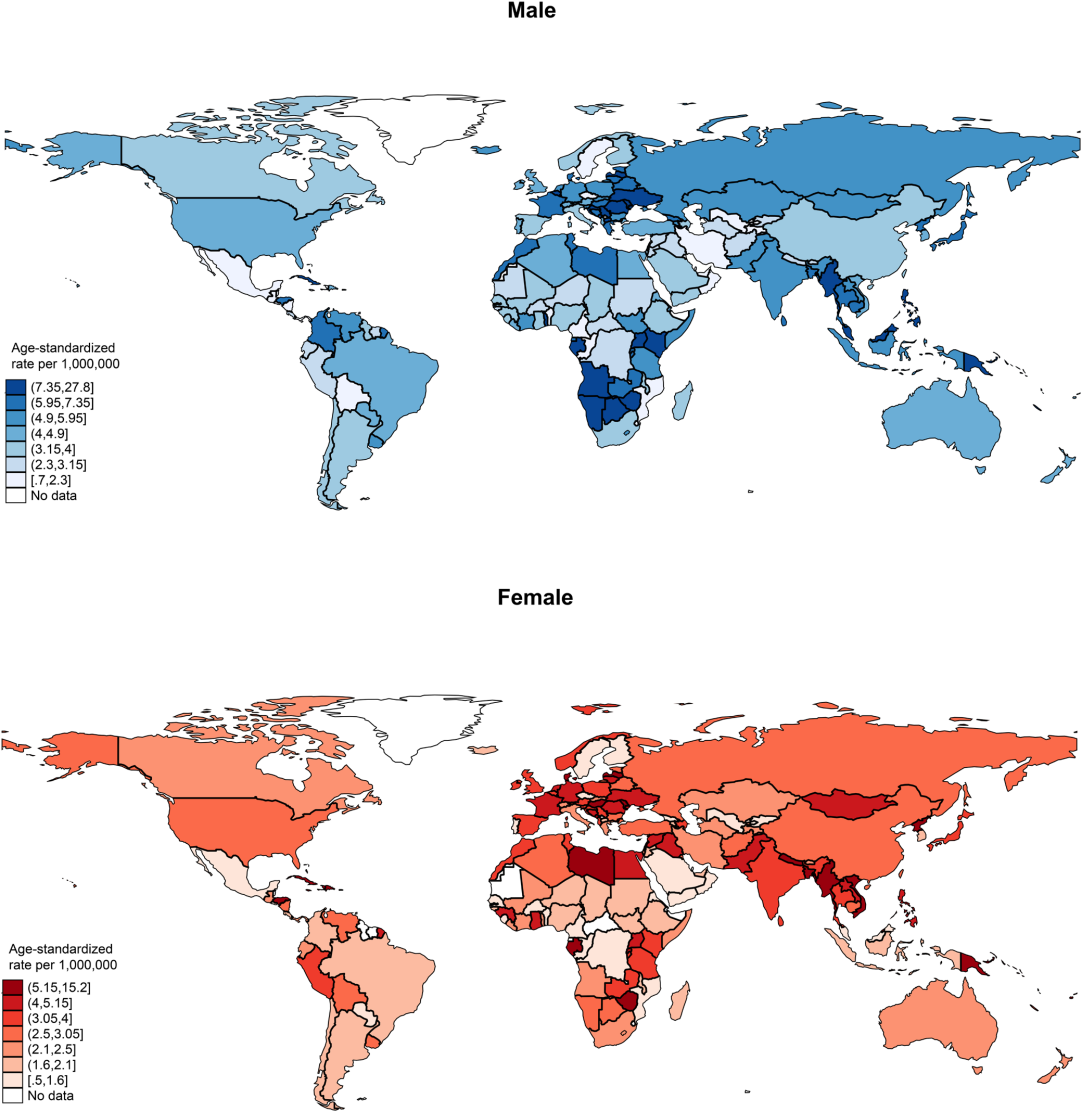
Global incidence of nasal cancer by sex, all ages, in 2020.

**FIGURE 2:**
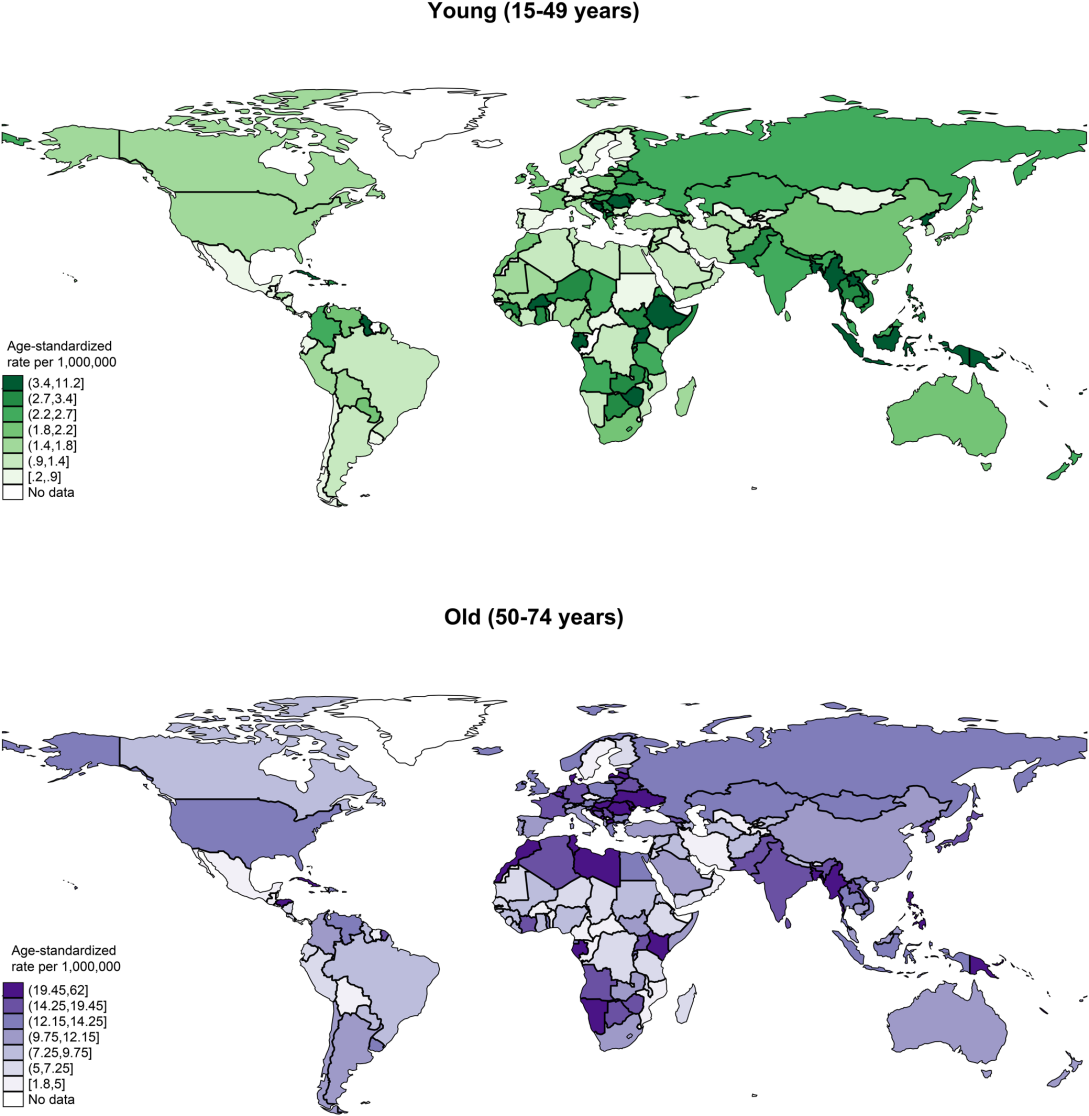
Global incidence of nasal cancer by age, both sexes, in 2020.

In 2020, the global incidence of nasal cancer in males was 21,891, with an ASR of 5.2, which was higher than that of the female population (case: 15,783, ASR: 3.6) (Figure 1, Table S1). In the male population, the five regions with the highest ASRs were South‐Eastern Asia (6.9 vs. 3.6 in females), Central and Eastern Europe (6.7 vs. 4.5), Northern Europe (6.2 vs. 3.1), Western Europe (6.0 vs. 4.4), and South‐Central Asia (5.3 vs. 4.1). Among the countries, the five with the highest ASRs were North Macedonia (27.8 vs. 2.1), Cabo Verde (26.4 vs. 10.4), Côte d'Ivoire (19.9 vs. 4.0), Namibia (15.9 vs. 3.0), and Uganda (15.1 vs. 4.3). As for the age group, the ASR incidence for the old population (case: 20,113, ASR: 13.4) was much higher than the young population (case: 8847, ASR: 2.4) (Figure 2, Table S1b). In the old population, the five regions with the highest ASRs were Western Europe (17.1 vs. 2.0 in young), Central and Eastern Europe (16.2 vs. 2.6), South‐Eastern Asia (15.6 vs. 3.9), Northern Africa (15.4 vs. 1.3), South‐Central Asia (14.6 vs. 2.5). Among the countries, the five with the highest ASRs were North Macedonia (62.0 vs. 5.4), Cabo Verde (43.2 vs. 15.4), French Guiana (38.1 vs. 2.0), Côte d'Ivoire (37.8 vs. 7.8), and Croatia (29.6 vs. 5.5).

### 3.3 Associations of risk factors with nasal cancer incidence

The overall nasal cancer incidence was linked to higher HDI (*β* = 0.254, CI 0.022–0.487, *p* = 0.032), higher prevalence of smoking (*β* = 0.086, CI 0.024–0.149, *p* = 0.007), alcohol drinking (*β* = 0.091, CI 0.024–0.159, *p* = 0.008), unhealthy dietary (*β* = 0.042, CI 0.011–0.074, *p* = 0.009), hypertension (*β* = 0.071, CI 0.031–0.111, *p* = 0.001, Table S2). Besides, a reverse association was found between nasal cancer and physical inactivity (*β* = −0.133, CI −0.246 to −0.021, *p* = 0.021). In multivariate analysis results, both hypertension (*β* = 0.006, CI 0.001–0.011, *p* = 0.015) and lipid (*β* = −0.006, CI −0.011 to 0.001, *p* = 0.036) demonstrated a significant association with the nasal cancer incidence.(Table S4).

### 3.4 Associations of risk factors with nasal cancer incidence by subgroup

**FIGURE 3:**
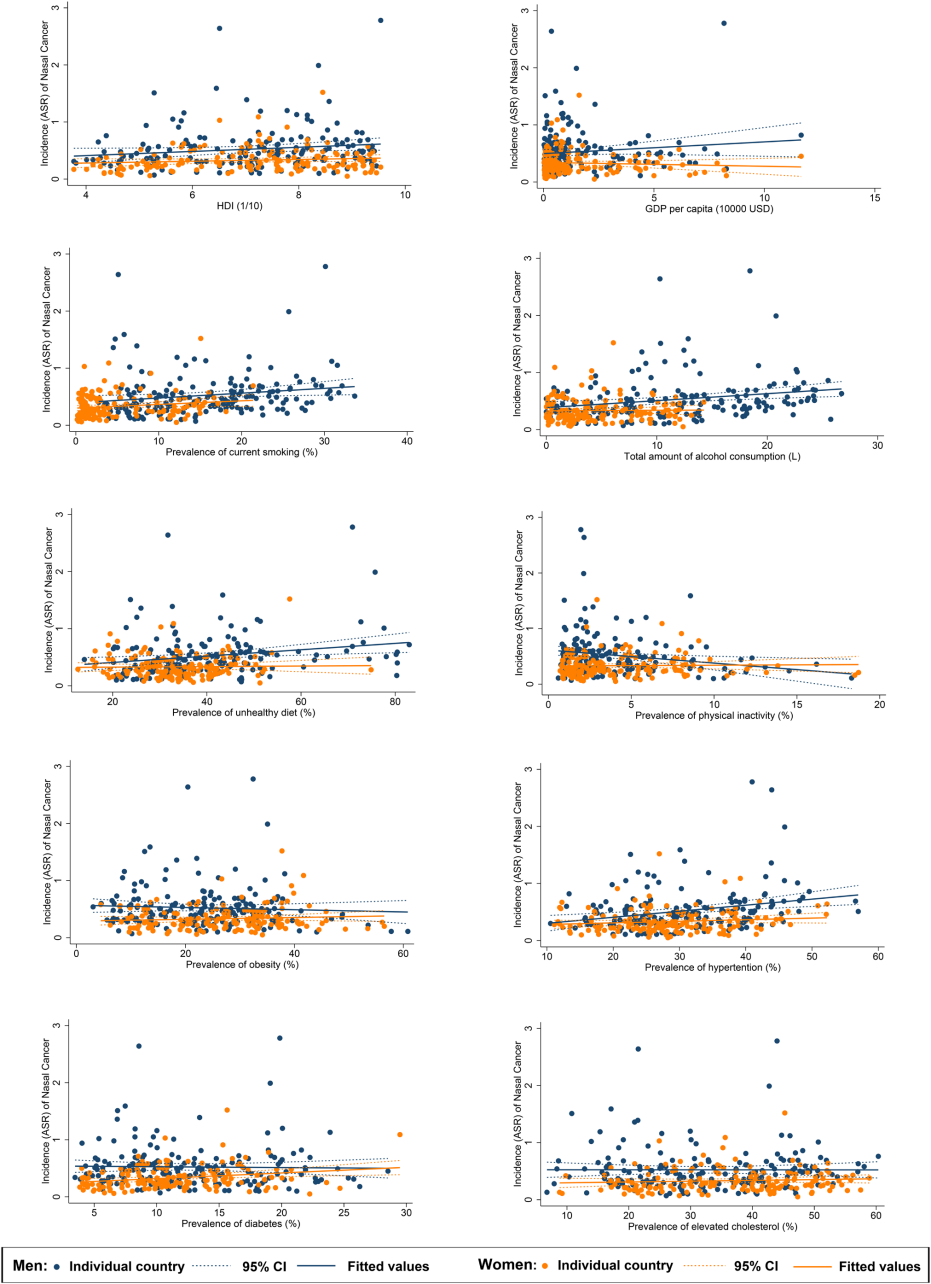
Associations between risk factors and nasal cancer by sex.

**FIGURE 4:**
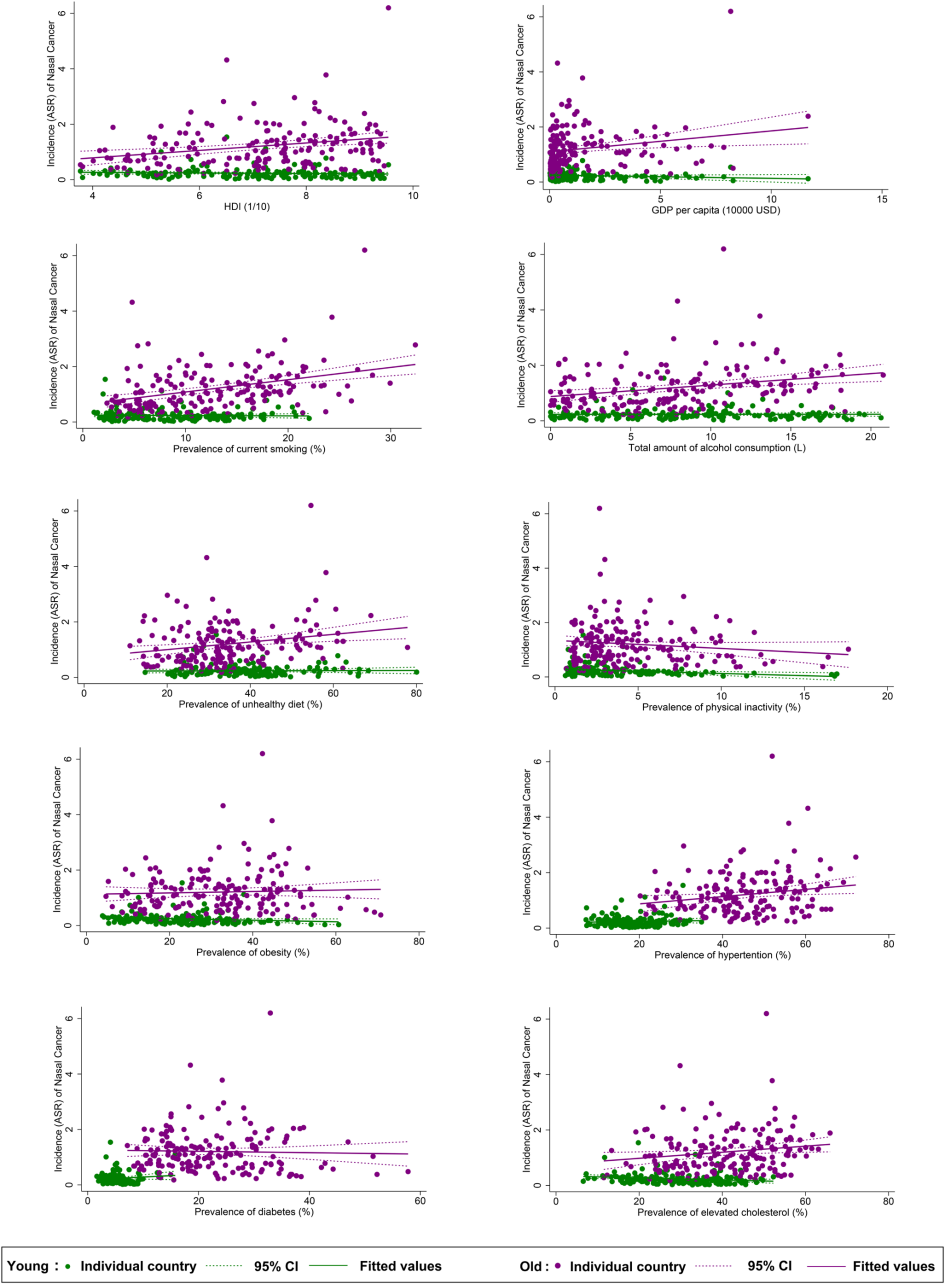
Associations between risk factors and nasal cancer by age.

The nasal cancer incidence for the male population was linked to the higher prevalence of smoking (*β* = 0.085, CI 0.013 to 0.157, *p* = 0.022), alcohol drinking (*β* = 0.122, CI 0.046–0.199, *p* = 0.002), unhealthy dietary (*β* = 0.056, CI 0.017–0.095, *p* = 0.005), and hypertension (*β* = 0.106, CI 0.046–0.166, *p* = 0.001, Figure 3). A reverse association was also found between nasal cancer and physical inactivity (*β* = −0.235, CI −0.414 to −0.055, *p* = 0.011). Among all risk factors examined, hypertension (*β* = 0.009, CI 0.001 to 0.016, *p* = 0.022) and lipid disorder (*β* = −0.012, CI −0.020 to −0.004, *p* = 0.005) were significantly related to nasal cancer incidence. As for females, only the higher prevalence of smoking (*β* = 0.064, CI 0.002–0.125, *p* = 0.042) and diabetes (*β* = 0.099, CI 0.032–0.165, *p* = 0.004) were linked to the nasal cancer incidence. The multivariate analysis revealed a negative relation between GDP per capital (*β* = −0.032, CI −0.056 to 0.007, *p* = 0.012) and the incidence while smoking (*β* = 0.011, CI 0.000–0.022, *p* = 0.041) and diabetes (*β* = 0.009, CI 0.001–0.018, *p* = 0.034) were positively significant. A negative association was found between nasal cancer incidence for the younger population and physical inactivity (*β* = −0.162, CI −0.261 to −0.064, *p* = 0.001), obesity (*β* = −0.028, CI −0.055 to −0.0005, *p* = 0.046), and lipid disorder (*β* = −0.037, CI −0.067 to −0.008, *p* = 0.013, Figure 4). Through multivariate analysis, diabetes (*β* = 0.023, CI 0.007–0.040, *p* = 0.007) and lipid disorder (*β* = −0.005, CI −0.010 to −0.001, *p* = 0.026) were respectively emerged as positive and negative risk factors. Turning to the older population, it was linked to higher HDI (*β* = 1.342, CI 0.602–2.082, *p* < 0.001), GDP per capita (*β* = 0.755, CI 0.182–1.329, *p* = 0.010), higher prevalence of smoking (*β* = 0.443, CI 0.278–0.609, *p* < 0.001), alcohol drinking (*β* = 0.413, CI 0.191–0.636, *p* < 0.001), unhealthy dietary (*β* = 0.138, CI 0.048–0.228, *p* = 0.003), hypertension (*β* = 0.131, CI 0.025–0.237, *p* = 0.016), lipid disorder (*β* = 0.110, CI 0.012–0.207, *p* = 0.028). And for multivariate analysis, the incidence was associated with high smoking (*β* = 0.044, CI 0.019–0.069, *p* = 0.001) as well as hypertension (*β* = 0.011, CI 0.000–0.022, *p* = 0.041).

### 3.5 Trend analysis of nasal cancer incidence

Globally, a declining trend was observed in nasal cancer, with 2 countries presented rising trends and 4 countries presented declining trends (Table S3, Figures S1 and S2). The greatest increases were found in Japan (AAPC: 2.45, 95% CI 1.05–3.88, *p* = 0.004), and the United Kingdom (AAPC: 2.37, 95% CI 1.06–3.69, *p* = 0.003). Meanwhile, the most significant declines were found in Bahrain (AAPC: −10.14, 95% CI −16.76 to −3.00, *p* = 0.012), followed by Colombia (AAPC: −7.94, 95% CI −12.79 to −2.83, *p* = 0.008), Thailand (AAPC: −6.13, 95% CI −11.70 to −0.22, *p* = 0.044) and China (AAPC: −4.06, 95% CI −7.53 to −0.45, *p* = 0.032).

### 3.6 Age‐ and sex‐specific trend analysis by subgroup

**FIGURE 5:**
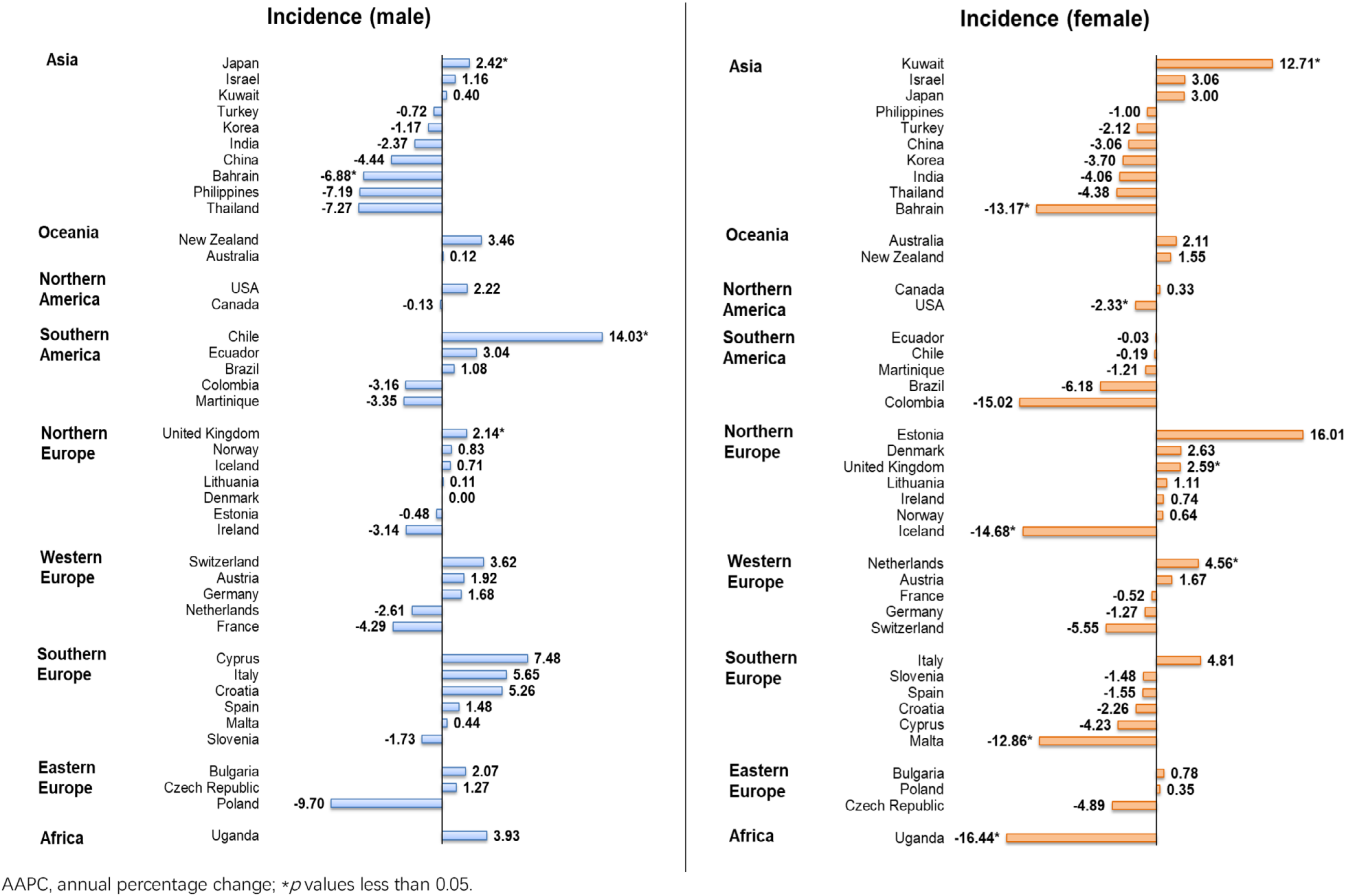
AAPC of nasal cancer incidence by sex, all ages.

An increasing trend was found among the male population, in which 3 countries reported increasing trends and only one country reported decreasing trend. A significant increasing trend was showed in Chile (AAPC: 14.03, 95% CI 9.91–18.30, *p* < 0.001), followed by Japan (AAPC: 2.42, 95% CI 0.08–4.81, *p* = 0.044) and United Kingdom (AAPC: 2.14, 95% CI 0.33–3.99, *p* = 0.026); while only Bahrain (AAPC: −6.88, 95% CI −11.95 to −1.52, *p* = 0.019) showed the decreases (Figure 5). In the female population, a decreasing trend was reported, in which three countries and five countries reported increasing and decreasing trends respectively. The increasing trends were shown in Kuwait (AAPC: 12.71, 95% CI 6.88–18.87, *p* = 0.001), followed by the Netherlands (AAPC: 4.56, 95% CI 1.26–7.98, *p* = 0.013) and the United Kingdom (AAPC: 2.59, 95% CI 1.46–3.73, *p* = 0.001). Besides, the declining trends were found in Uganda (AAPC: −16.44, 95% CI −26.12 to −5.49, *p* = 0.010), Iceland (AAPC: −14.68, 95% CI −23.50 to −4.84, *p* = 0.010), Bahrain (AAPC: −13.17, 95% CI −24.36 to −0.34, *p* = 0.045), Malta (AAPC: −12.86, 95% CI −19.78 to −5.34, *p* = 0.005) and the USA (AAPC: −2.33, 95% CI −4.26 to −0.36, *p* = 0.026).

**FIGURE 6:**
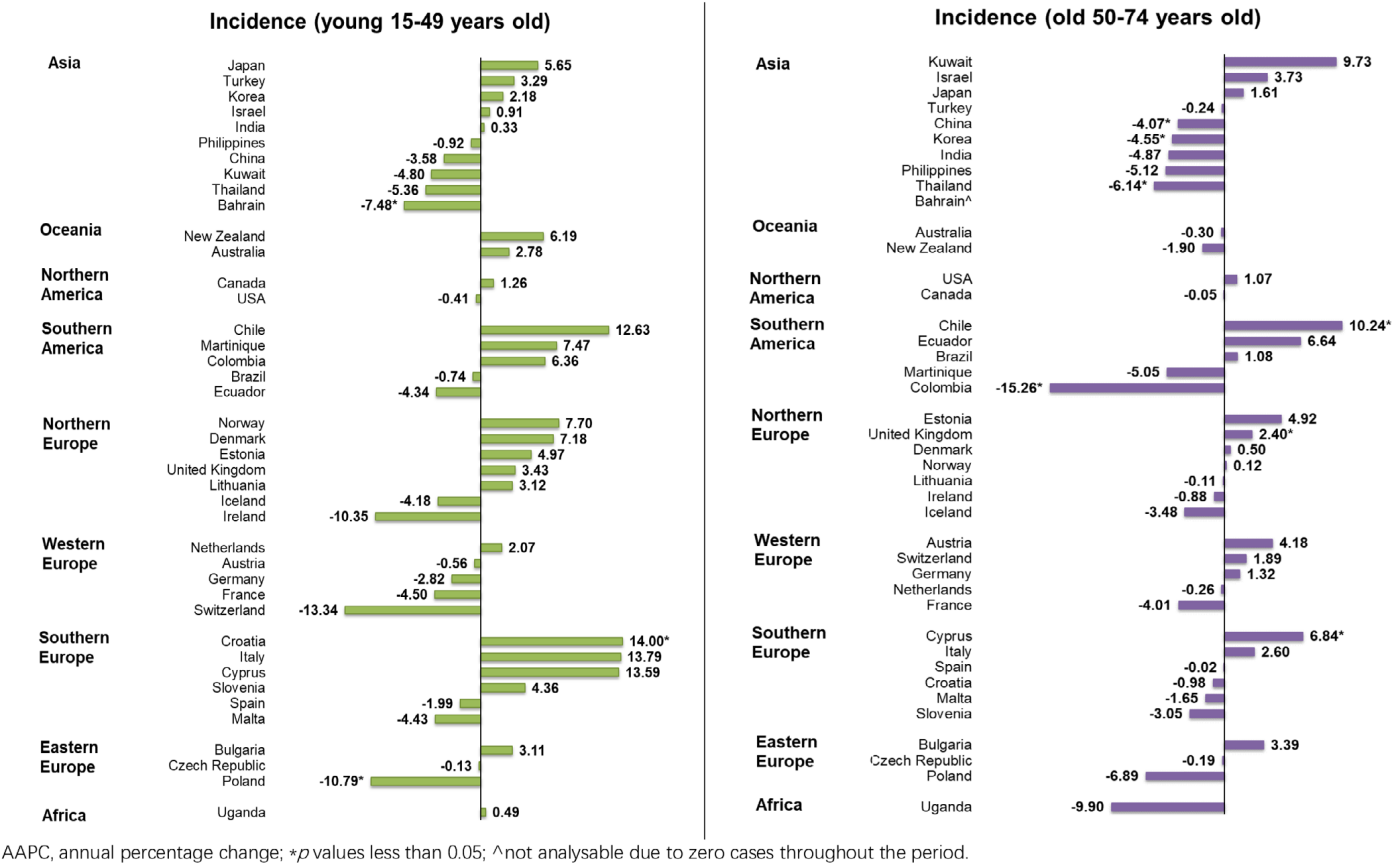
AAPC of nasal cancer incidence by ages, both sexes.

In terms of age, a mixed trend was reported in the young population, in which one country reported a rising trend and two countries reported decreasing trends (Figure 6). The decreasing trend was reported in Croatia only (AAPC: 14.00, 95% CI 2.37–26.95, *p* = 0.023); meanwhile, Poland (AAPC: −10.79, 95% CI −19.39 to −1.26, *p* = 0.032) and Bahrain (AAPC: −7.48, 95% CI −12.68 to −1.98, *p* = 0.015) reported the significant declines. Similarly, a mixed trend was reported in the old population, in which three countries showed rising trends and four countries showed diminishing trends: Chile (AAPC: 10.24, 95% CI 6.52–14.10, *p* < 0.001), Cyprus (AAPC: 6.84, 95% CI 0.60–13.47, *p* = 0.035) and United Kingdom (AAPC: 2.40, 95% CI 0.69–4.14, *p* = 0.012) reported the growing trends; Colombia (AAPC: −15.26, 95% CI −22.98 to −6.77, *p* = 0.004) reported the most significant decrease, followed by Thailand (AAPC: −6.14, 95% CI −11.05 to −0.96, *p* = 0.026), Korea (AAPC: −4.55, 95% CI −8.01 to −0.96, *p* = 0.020) and China (AAPC: −4.07, 95% CI −7.52 to −0.50, *p* = 0.031).

## 4 DISCUSSION

### 4.1 Summary of major findings

In the present study, the global incidence, associated risk factors, and the temporal trends of nasal cancer across various subgroups were concluded. We found that there is a considerable variation in the disease burden across different geographic regions, with a higher incidence reported in South‐Eastern Asia and Central and Eastern Europe. Second, several risk factors were identified, such as regions with higher HDI, higher prevalence of smoking, alcohol drinking, unhealthy dietary, and hypertension. In addition, physical inactivity was found to be a protective factor for male and young populations. Third, an overall decreasing trend was reported in the global population, but an increasing trend was discovered in males.

### 4.2 Variation in the disease burden

The current study concluded that South‐Eastern Asia and Central and Eastern Europe reported the highest incidence globally. Genetic factors may serve as one of the possible reasons for the findings. Several studies have suggested that a gene associated with an increased susceptibility to nasopharyngeal cancer is inherited among the Bai‐yue population, a group of Southern Natives in China.^16^, ^17^, ^18^ This ethnic minority group, which has a higher risk of developing the disease, has been residing in the South‐eastern part of Asia, such as Borneo (Bidayuh) and Hainan island (Tanka boat people).^19^ Besides, environmental exposure may contribute to the higher disease burden in Central and Eastern Europe. In this region, the household usage of coal for heating and wood for cooking is common,^20^ this has resulted in the region becoming the most polluted area in Europe.^21^ From a previous study in Central and Eastern Europe, indoor air pollution has been found to increase the cancer risk in the upper aerodigestive tract, including the oral cavity, larynx, pharynx, and esophagus.^22^ The risk is 4.05 times higher of developing pharyngeal cancer (95% CI: 1.30–12.68), and 2.71 times higher of developing esophageal cancer (95% CI: 1.21–6.10) in people who consistently used wood. Likewise, those who consistently used coal had a 5.37 times higher risk of developing laryngeal cancer (95% CI: 2.39–12.04) and a 4.13 times higher risk of developing head & neck cancers (95% CI: 1.99–8.55).^22^ In relation to nasopharyngeal cancer, a positive association was drawn between nasopharyngeal cancer and indoor pollution including the use of wood (OR 1.34, 95% CI: 1.03–1.75) and coal (OR 1.70, 95% CI: 1.17–2.47).^23^ The common use of coal and wood for cooking and heating is thought to be a possible contributing factor to the high incidence of nasal cancer cases reported in the Central and Eastern European regions.

### 4.3 Risk factors associated with nasal cancer

Smoking has been a well‐confirmed risk factor for nasal cancer. Compared with the nonsmokers, the risk was found to increase by 60% among individuals who had ever smoked (OR: 1.60, 95% CI 1.24–2.07).^24^ This risk was even higher in male smokers, with a more than two times risk that of nonsmokers (OR 2.4, 95% CI 1.1–5.2, *p* < 0.01).^3^ The current study also proved this correlation in all subgroups, except the younger population. There is an inconsistent finding of alcohol consumption and nasal cancer in the previous studies. More nasal cancer cases than controls reported a history of alcohol consumption, particularly in the highest alcohol‐intake group (⩾300 ounces per year: OR 2.0, 95% CI 1.0–3.9).^3^ However, a lower risk was reported in the middle alcohol‐intake group in the same study (75 to 149.9 ounces per year: OR 0.7, 95% CI 0.3–1.7; 150–299.9 ounces per year: OR 0.9, 95% CI 0.4–2.0). The prior studies have also shown that unhealthy dietary are related to an increased risk for nasal cancer, including inflammatory diet pattern (OR: 1.64, 95% CI: 1.06–2.55; *p* = 0.04),^25^ and meat‐heavy diet (OR 2.26, 95% CI: 1.85–2.77).^23^

Our study revealed an interesting finding that physical inactivity is related to a lower risk of nasal cancer. This could be due to people with high physical activity in outdoor environment could have more exposure to air pollutants and chemicals, especially Epstein–Barr virus (EBV). In early studies, it is proven that EBV increases the risk of nasopharyngeal cancers in Asian countries,^23^, ^26^ and some countries in North America.^23^, ^27^, ^28^ Regarding nasopharyngeal cancer, those exposed to the highest concentration of air pollutants had a significantly higher risk of developing nasopharyngeal cancers, with an incidence rate of 1.88 for NO_2_ (95% CI 1.09–3.22, compared to the lowest concentration group) and 2.08 for PM2.5 (95% CI 1.21–3.58, compared to the lowest concentration group).^29^ However, the relationship between exercise and health outcomes is complex. Despite the potential negative effects of exercising in a polluted environment, the benefits of physical activity may outweigh the risks associated with exposure to air pollution.

### 4.4 Incidence trends of nasal cancer

Different countries and regions have reported a declining trend in nasal cancer incidence. A significant decreasing incidence rates for nasopharyngeal (APC: −7.89%, 95% CI = −9.43% to −6.31%, *p* < 0.001) and sinonasal cancer (APC: −10.08%, 95% CI = −16.66% to −2.99%, *p* = 0.012) was reported in Taiwan during 2010–2018.^30^ Between 1993 and 2015 in Japan, there was a declining trend of nasopharyngeal cancer in males (nasopharynx: APC −2.7%, 95% CI: −4.6% to −0.7%), but no significant trend was identified for nasal and paranasal cavity cancer.^31^ A review paper on the international patterns in sinonasal cancer incidence found that 11 countries and 8 countries showed declining trends in 14 selected countries or regions for male and female populations, respectively.^32^ This is partially related to HDI indicating a country's access to more multidisciplinary teams (MDT), involving medical specialties' participation in treatment and health systems as well as cooperation and coordination among teams for better head and neck cancer care and management services.^33^ Also, there's possibility that countries with comparably high HDIs have more comprehensive treatment information and strategies for better nasal care services.^34^ There is limited research on the rising trend of nasal cancer in males, but the possible reason could be the increasing prevalence of risk factors.^24^, ^35^

## 5 STRENGTH AND LIMITATIONS

This study utilized high‐quality data from cancer registries in 186 countries to conduct a comprehensive analysis of the incidence, risk factors, and temporal trends of nasal cancer on a global scale. The study has several limitations related to its data source, study design, and statistical analysis. Firstly, We used ICD‐10 C30 to identify malignant neoplasm of nasal cavity to ensure the consistency of different registries. Therefore, we did not include malignant neoplasm of nasopharynx (C11) in the analysis. Secondly, the issue of underreported or misclassified cancer cases is a concern. Inadequate cancer data quality, registry coverage, and analytical capacity in low‐ and low‐middle‐income countries (LMICs) may lead to under‐documentation and misclassification. Thirdly, the confounding variables may influence the accuracy of the risk factor analysis. Since the present study relied mainly on univariate analysis, which limits the ability to determine non‐linear associations between risk factors and nasal cancer. Additionally, the direct comparisons of country‐ and site‐specific outcomes may be difficult or unreliable due to the changes in cancer registries over time, such as alterations to the methodology of data collection, registration criteria, or population coverage. In spite of that, it is relatively dependable to compare the country‐ and site‐specific outcomes during the same time period.

## 6 CONCLUSION

The highest burden of nasal cancer was found in South‐Eastern Asia and Central and Eastern Europe, potentially due to regional genetic factors and pollution issues, highlighting the need for targeted interventions in high‐risk regions. The impact of smoking, unhealthy dietary pattern, and outdoor air pollution of developing nasal cancer has been identified, this informs the development of preventive strategies and health promotion campaigns. Education should be provided to individuals in high‐risk regions about the health risk associated with smoking, unhealthy diets, and exercising in polluted environments. Further studies are needed to investigate factors contributing to the increasing temporal trend of nasal cancer among the male population.

## AUTHOR CONTRIBUTIONS

**Junjie Huang**: conceptualisation, supervision, and writing–original draft. **Wing Sze Pang**: data curation, formal analysis, and writing–original draft. **Fung Yu Mak**: writing–original draft. **Sze Chai Chan**: data curation and formal analysis. **Veeleah Lok**: writing–review and editing. **Lin Zhang**: writing–review and editing. **Xu Lin**: writing–review and editing. **Don Eliseo Lucero**‐**Prisno III**: writing–review and editing. **Wanghong Xu**: writing‐review and editing. **Zhi‐Jie Zheng**: writing–review and editing. **Edmar Elcarte**: writing–review and editing. **Mellissa Withers**: writing–review and editing. **Martin CS Wong**: conceptualisation, supervision, and writing–review and editing.

## FUNDING INFORMATION

This research received no grant from any funding agency in the public, commercial, or not‐for profit sectors.

## CONFLICT OF INTEREST STATEMENT

The authors have declared no conflicts of interest.

## ETHICS STATEMENT

This study was approved by the Survey and Behavioral Research Ethics Committee, The Chinese University of Hong Kong (No. SBRE‐20‐332).

## Supporting information


Table S1.

## Data Availability

The data that supports findings of this study are available from the corresponding author, upon reasonable request.
