# Supplementary material for: Global incidence, risk factors, and temporal trends of nasal cancer: A population‐based analysis
Source: Cancer Med. 2025 Apr 17;14(8):e70163. doi: 10.1002/cam4.70163 (PMC12004393; doi:10.1002/cam4.70163)
Supplement: Supplementary file 1 — Table S1. [file CAM4-14-e70163-s001.docx]

**Supplementary Legends**

**Supplementary Table 1a.** Global incidence of nasal cancer by sex **Supplementary Table 1b.** Global incidence of nasal cancer by age **Supplementary Table 2.** Results of risk factors associations with nasal cancer **Supplementary Table 3.** Results of Joinpoint regression for trend analysis

**Supplementary Table 4**. Multivariate Analysis Results for risk factors of nasal cancer

**Supplementary Figure 1.** Incidence trends for individual countries **Supplementary Figure 2.** Plots of Joinpoint regression for trend analysis

**Supplementary Table 1a.** Global incidence of nasal cancer by sex

|  | Both sexes |  | Males |  | Females |  |
| --- | --- | --- | --- | --- | --- | --- |
| Region |  |  |  |  |  |  |
|  | **New cases** | **ASR** | **New cases** | **ASR** | **New cases** | **ASR*** |
| **World** | 37,674 | 4.2 | 21,891 | 5.2 | 15,783 | 3.6 |
| **Asia** | 23,104 | 4.3 | 13,455 | 5.3 | 9,649 | 3.8 |
| **Eastern Asia** | 10,223 | 3.8 | 5,992 | 4.8 | 4,231 | 3.7 |
| China | 7,881 | 3.2 | 4,534 | 4.0 | 3,347 | 2.8 |
| Japan | 1,791 | 4.8 | 1,139 | 6.7 | 652 | 3.7 |
| Korea, Democratic Republic of | 214 | 4.6 | 96 | 6.0 | 118 | 5.8 |
| Korea, Republic of | 324 | 3.1 | 217 | 4.5 | 107 | 1.8 |
| Mongolia | 13 | 3.8 | 6 | 5.4 | 7 | 5.1 |
| **South-Eastern Asia** | 3,586 | 5.3 | 2,217 | 6.9 | 1,369 | 3.6 |
| Brunei Darussalam | 2 | 3.2 | 1 | 3.3 | 1 | 4.8 |
| Cambodia | 53 | 4.3 | 32 | 6.0 | 21 | 2.8 |
| Indonesia | 1,056 | 4.3 | 763 | 5.8 | 293 | 2.0 |
| Lao People's Democratic Republic | 29 | 4.9 | 15 | 5.8 | 14 | 5.1 |
| Malaysia | 153 | 4.6 | 125 | 7.4 | 28 | 1.6 |
| Myanmar | 453 | 8.6 | 273 | 11.6 | 180 | 6.2 |
| Philippines | 606 | 6.9 | 392 | 9.8 | 214 | 4.3 |
| Singapore | 43 | 4.3 | 29 | 5.4 | 14 | 2.3 |
| Thailand | 570 | 4.5 | 336 | 6.0 | 234 | 3.4 |
| Timor-Leste | 1 | 2.2 | 1 | 3.1 |  | 0.0 |
| Viet Nam | 620 | 3.9 | 250 | 4.7 | 370 | 5.5 |
| **South-Central Asia** | 8,502 | 4.5 | 4,809 | 5.3 | 3,693 | 4.1 |
| Afghanistan | 50 | 2.4 | 24 | 2.7 | 26 | 2.6 |
| Bangladesh | 917 | 6.2 | 528 | 7.3 | 389 | 5.6 |
| Bhutan | 2 | 2.7 | 1 | 3.0 | 1 | 2.2 |
| India | 6,135 | 4.5 | 3,550 | 5.3 | 2,585 | 3.8 |
| Iran, Islamic Republic of | 177 | 2.0 | 78 | 1.8 | 99 | 2.4 |
| Kazakhstan | 80 | 4.0 | 53 | 5.9 | 27 | 2.3 |
| Kyrgyzstan | 10 | 2.0 | 6 | 2.8 | 4 | 1.3 |
| Maldives | 1 | 2.5 | 1 | 3.5 |  | 0.0 |

| Nepal | 111 | 2.9 | 34 | 3.1 | 77 | 5.6 |
| --- | --- | --- | --- | --- | --- | --- |
| Pakistan | 814 | 4.9 | 424 | 5.6 | 390 | 5.0 |
| Sri Lanka | 138 | 4.5 | 84 | 6.1 | 54 | 3.1 |
| Tajikistan | 10 | 0.84 | 2 | 0.70 | 8 | 2.4 |
| Turkmenistan | 14 | 2.4 | 7 | 3.1 | 7 | 2.4 |
| Uzbekistan | 43 | 1.1 | 17 | 1.4 | 26 | 1.6 |
| **Western Asia** | 793 | 3.0 | 437 | 3.9 | 356 | 2.7 |
| Armenia | 14 | 3.3 | 9 | 5.1 | 5 | 1.8 |
| Azerbaijan | 45 | 3.0 | 21 | 3.9 | 24 | 3.7 |
| Bahrain | 1 | 0.57 | 1 | 1.0 |  | 0.0 |
| Gaza Strip and West Bank | 8 | 2.9 | 5 | 3.7 | 3 | 2.1 |
| Georgia | 24 | 4.4 | 19 | 6.9 | 5 | 0.86 |
| Iraq | 79 | 2.5 | 27 | 2.9 | 52 | 4.6 |
| Israel | 34 | 3.0 | 20 | 3.7 | 14 | 2.5 |
| Jordan | 19 | 2.5 | 11 | 3.1 | 8 | 2.0 |
| Kuwait | 8 | 2.3 | 5 | 2.2 | 3 | 2.7 |
| Lebanon | 29 | 2.2 | 8 | 2.1 | 21 | 6.1 |
| Oman | 5 | 1.3 | 3 | 1.2 | 2 | 1.4 |
| Qatar | 1 | 1.2 | 1 | 1.1 |  | 0.0 |
| Saudi Arabia | 69 | 2.7 | 53 | 3.6 | 16 | 1.6 |
| Syrian Arab Republic | 51 | 2.5 | 18 | 2.9 | 33 | 4.7 |
| Turkey | 369 | 3.3 | 210 | 4.6 | 159 | 2.9 |
| United Arab Emirates | 5 | 1.3 | 3 | 1.1 | 2 | 2.0 |
| Yemen | 32 | 2.4 | 23 | 3.2 | 9 | 0.76 |
| **Oceania** | 256 | 4.0 | 157 | 5.2 | 99 | 3.1 |
| Australia | 166 | 3.6 | 110 | 4.9 | 56 | 2.4 |
| Fiji | 7 | 4.9 | 2 | 4.5 | 5 | 10.9 |
| France, New Caledonia | 3 | 9.4 | 2 | 14.3 | 1 | 3.1 |
| French Polynesia | 3 | 7.6 | 2 | 10.2 | 1 | 3.3 |
| Guam | 0 | 2.7 | 0 | 4.1 |  | 0.0 |
| New Zealand | 31 | 3.5 | 20 | 4.8 | 11 | 2.3 |
| Papua New Guinea | 44 | 6.4 | 21 | 8.2 | 23 | 6.7 |
| Samoa | 0 | 1.6 | 0 | 3.0 |  | 0.0 |

| Solomon Islands | 2 | 3.0 | 0 | 3.0 | 2 | 5.8 |
| --- | --- | --- | --- | --- | --- | --- |
| Vanuatu | 0 | 4.0 | 0 | 5.7 |  | 0.0 |
| **Northern America** | 2,456 | 3.6 | 1,477 | 4.5 | 979 | 2.9 |
| Canada | 233 | 3.1 | 136 | 3.6 | 97 | 2.4 |
| United States of America | 2,223 | 3.7 | 1,341 | 4.7 | 882 | 3.0 |
| **Latin America and the Caribbean** | 2,230 | 3.8 | 1,260 | 4.6 | 970 | 3.5 |
| **Central America & Caribbean** | 495 | 2.8 | 198 | 3.2 | 297 | 3.3 |
| Bahamas | 1 | 3.1 | 1 | 4.4 |  | 0.0 |
| Barbados | 1 | 1.7 | 1 | 2.4 |  | 0.0 |
| Belize | 0 | 1.4 | 0 | 1.7 |  | 0.0 |
| Costa Rica | 17 | 2.3 | 9 | 2.7 | 8 | 2.0 |
| Cuba | 8 | 4.2 | 5 | 5.1 | 3 | 2.5 |
| Dominican Republic | 69 | 4.5 | 25 | 4.7 | 44 | 7.1 |
| El Salvador | 10 | 0.90 | 4 | 1.1 | 6 | 1.0 |
| France, Guadeloupe | 3 | 3.5 | 2 | 4.9 | 1 | 0.64 |
| France, Martinique | 2 | 2.4 | 1 | 2.9 | 1 | 1.2 |
| Guatemala | 23 | 1.1 | 7 | 1.1 | 16 | 2.4 |
| Haiti | 31 | 1.5 | 5 | 1.2 | 26 | 5.2 |
| Honduras | 46 | 6.1 | 24 | 7.2 | 22 | 5.9 |
| Jamaica | 20 | 4.6 | 2 | 1.0 | 18 | 7.8 |
| Mexico | 220 | 1.3 | 91 | 1.4 | 129 | 1.6 |
| Nicaragua | 16 | 1.9 | 5 | 2.2 | 11 | 2.9 |
| Panama | 10 | 1.5 | 4 | 1.6 | 6 | 2.2 |
| Puerto Rico | 15 | 2.4 | 9 | 3.3 | 6 | 1.5 |
| Saint Lucia | 0 | 1.9 | 0 | 2.6 |  | 0.0 |
| Trinidad and Tobago | 3 | 2.3 | 3 | 3.1 |  | 0.0 |
| **South America** | 1,735 | 4.2 | 1,062 | 5.3 | 673 | 3.5 |
| Argentina | 153 | 2.7 | 91 | 3.5 | 62 | 1.8 |
| Bolivia, Plurinational State of | 29 | 2.0 | 12 | 2.2 | 17 | 2.9 |
| Brazil | 872 | 3.0 | 549 | 4.4 | 323 | 2.1 |
| Chile | 89 | 2.8 | 51 | 3.8 | 38 | 1.8 |
| Colombia | 236 | 3.9 | 173 | 6.1 | 63 | 1.8 |
| Ecuador | 53 | 2.7 | 25 | 2.8 | 28 | 2.5 |

| French Guiana | 2 | 8.0 | 2 | 13.3 |  | 0.0 |
| --- | --- | --- | --- | --- | --- | --- |
| Guyana | 2 | 2.7 | 2 | 3.9 |  | 0.0 |
| Paraguay | 19 | 3.1 | 13 | 4.1 | 6 | 1.4 |
| Peru | 122 | 2.4 | 46 | 2.4 | 76 | 4.0 |
| Suriname | 1 | 1.6 | 1 | 2.7 |  | 0.0 |
| Uruguay | 21 | 3.6 | 13 | 5.0 | 8 | 2.6 |
| Venezuela, Bolivarian Republic of | 136 | 4.4 | 84 | 5.7 | 52 | 3.0 |
| **Europe** | 6,727 | 4.4 | 3,828 | 6.1 | 2,899 | 3.9 |
| **Northern Europe** | 830 | 3.9 | 480 | 6.2 | 350 | 3.1 |
| Denmark | 74 | 6.1 | 39 | 7.0 | 35 | 5.7 |
| Estonia | 11 | 4.3 | 7 | 6.3 | 4 | 2.6 |
| Finland | 28 | 1.9 | 20 | 3.3 | 8 | 1.1 |
| Iceland | 3 | 4.0 | 2 | 5.8 | 1 | 1.7 |
| Ireland | 33 | 4.1 | 19 | 4.9 | 14 | 3.3 |
| Latvia | 27 | 6.7 | 13 | 8.6 | 14 | 6.4 |
| Lithuania | 30 | 4.8 | 16 | 6.9 | 14 | 4.6 |
| Norway | 42 | 3.7 | 22 | 4.0 | 20 | 4.0 |
| Sweden | 37 | 1.5 | 26 | 2.3 | 11 | 1.1 |
| United Kingdom | 545 | 4.1 | 316 | 4.9 | 229 | 3.4 |
| **Western Europe** | 2,035 | 4.7 | 1,149 | 6.0 | 886 | 4.4 |
| Austria | 71 | 3.8 | 42 | 4.9 | 29 | 3.4 |
| Belgium | 125 | 5.6 | 89 | 8.1 | 36 | 3.5 |
| France | 697 | 5.0 | 415 | 6.9 | 282 | 4.5 |
| Germany | 902 | 4.2 | 470 | 5.1 | 432 | 4.8 |
| Luxembourg | 6 | 6.2 | 4 | 8.2 | 2 | 4.5 |
| Switzerland | 61 | 3.1 | 35 | 4.2 | 26 | 2.5 |
| The Netherlands | 173 | 4.5 | 94 | 5.2 | 79 | 4.3 |
| **Southern Europe** | 1,428 | 3.5 | 839 | 4.7 | 589 | 2.8 |
| Albania | 21 | 3.6 | 11 | 4.7 | 10 | 3.8 |
| Bosnia and Herzegovina | 37 | 5.2 | 20 | 6.9 | 17 | 4.8 |
| Croatia | 230 | 9.0 | 120 | 12.0 | 110 | 9.1 |
| Cyprus | 42 | 1.6 | 26 | 2.6 | 16 | 1.3 |
| Greece | 96 | 4.2 | 64 | 6.0 | 32 | 2.4 |

| Italy | 447 | 3.0 | 261 | 4.0 | 186 | 2.5 |
| --- | --- | --- | --- | --- | --- | --- |
| Malta | 2 | 3.1 | 1 | 2.8 | 1 | 3.3 |
| Montenegro | 10 | 8.4 | 6 | 11.2 | 4 | 6.9 |
| North Macedonia | 49 | 14.5 | 45 | 27.8 | 4 | 2.1 |
| Portugal | 61 | 3.0 | 42 | 4.4 | 19 | 1.6 |
| Serbia | 95 | 5.6 | 57 | 7.6 | 38 | 3.8 |
| Slovenia | 18 | 4.2 | 10 | 5.1 | 8 | 3.7 |
| Spain | 320 | 2.9 | 176 | 3.9 | 144 | 3.2 |
| **Central and Eastern Europe** | 2,434 | 4.8 | 1,360 | 6.7 | 1,074 | 4.5 |
| Belarus | 75 | 4.7 | 47 | 7.0 | 28 | 2.8 |
| Bulgaria | 55 | 3.7 | 36 | 5.5 | 19 | 2.9 |
| Czechia | 14 | 1.3 | 11 | 1.8 | 3 | 0.48 |
| Hungary | 196 | 6.1 | 59 | 7.2 | 137 | 15.2 |
| Poland | 304 | 3.9 | 165 | 5.1 | 139 | 3.6 |
| Republic of Moldova | 47 | 7.5 | 29 | 10.5 | 18 | 6.1 |
| Romania | 260 | 7.5 | 159 | 10.1 | 101 | 5.1 |
| Russian Federation | 993 | 3.8 | 575 | 5.7 | 418 | 2.9 |
| Slovakia | 38 | 4.3 | 26 | 5.9 | 12 | 2.4 |
| Ukraine | 452 | 5.5 | 253 | 8.0 | 199 | 4.8 |
| **Africa** | 2,901 | 3.9 | 1,714 | 4.9 | 1,187 | 2.8 |
| **Northern Africa** | 886 | 4.3 | 468 | 4.8 | 418 | 3.7 |
| Algeria | 153 | 4.3 | 90 | 4.4 | 63 | 2.9 |
| Egypt | 356 | 3.9 | 156 | 4.1 | 200 | 4.6 |
| Libya | 32 | 6.2 | 17 | 6.7 | 15 | 5.6 |
| Morocco | 215 | 6.2 | 130 | 6.7 | 85 | 4.0 |
| Sudan | 60 | 2.3 | 30 | 2.4 | 30 | 2.1 |
| Tunisia | 70 | 5.8 | 45 | 6.5 | 25 | 3.3 |
| **Sub-Saharan Africa** | 2,015 | 3.7 | 1,246 | 5.1 | 769 | 2.5 |
| Angola | 75 | 6.1 | 55 | 9.1 | 20 | 2.6 |
| Benin | 9 | 1.4 | 6 | 2.3 | 3 | 0.56 |
| Botswana | 9 | 7.2 | 7 | 11.9 | 2 | 2.7 |
| Burkina Faso | 18 | 1.9 | 12 | 2.8 | 6 | 1.1 |
| Burundi | 26 | 4.9 | 14 | 6.5 | 12 | 3.4 |

| Cabo Verde | 7 | 17.1 | 5 | 26.4 | 2 | 10.4 |
| --- | --- | --- | --- | --- | --- | --- |
| Cameroon | 18 | 1.2 | 9 | 1.3 | 9 | 1.3 |
| Central African Republic | 3 | 1.7 | 3 | 2.8 |  | 0.0 |
| Chad | 17 | 2.7 | 11 | 3.4 | 6 | 1.7 |
| Comoros | 0 | 0.0 |  | 0.0 |  | 0.0 |
| Congo, Democratic Republic of | 102 | 2.3 | 61 | 3.1 | 41 | 1.6 |
| Congo, Republic of | 4 | 1.3 | 2 | 1.7 | 2 | 1.0 |
| Côte d'Ivoire | 94 | 13.3 | 75 | 19.9 | 19 | 4.0 |
| Djibouti | 2 | 3.0 | 2 | 4.5 |  | 0.0 |
| Equatorial Guinea | 2 | 4.6 | 2 | 6.8 |  | 0.0 |
| Eritrea | 7 | 3.3 | 5 | 4.8 | 2 | 1.0 |
| Eswatini | 0 | 0.68 | 0 | 1.2 |  | 0.0 |
| Ethiopia | 189 | 3.0 | 127 | 4.0 | 62 | 1.7 |
| France, La Réunion | 11 | 9.9 | 10 | 14.8 | 1 | 1.1 |
| Gabon | 13 | 10.6 | 9 | 13.9 | 4 | 6.2 |
| Ghana | 79 | 3.6 | 36 | 3.7 | 43 | 4.9 |
| Guinea | 27 | 3.1 | 10 | 3.4 | 17 | 4.4 |
| Guinea-Bissau | 2 | 2.1 | 1 | 2.8 | 1 | 1.7 |
| Kenya | 155 | 6.8 | 111 | 10.2 | 44 | 3.3 |
| Lesotho | 3 | 3.0 | 3 | 5.4 |  | 0.0 |
| Liberia | 9 | 3.5 | 6 | 4.8 | 3 | 2.2 |
| Madagascar | 42 | 2.8 | 24 | 3.7 | 18 | 2.2 |
| Malawi | 24 | 2.8 | 14 | 4.0 | 10 | 2.2 |
| Mali | 26 | 2.8 | 14 | 3.7 | 12 | 2.2 |
| Mauritania | 4 | 1.9 | 4 | 3.1 |  | 0.0 |
| Mauritius | 14 | 7.6 | 11 | 11.4 | 3 | 2.6 |
| Mozambique | 29 | 1.6 | 15 | 2.3 | 14 | 1.2 |
| Namibia | 11 | 9.5 | 9 | 15.9 | 2 | 3.0 |
| Niger | 24 | 2.5 | 16 | 3.1 | 8 | 1.7 |
| Nigeria | 304 | 3.0 | 182 | 3.7 | 122 | 2.2 |
| Rwanda | 41 | 4.9 | 21 | 6.8 | 20 | 4.2 |
| Sao Tome and Principe | 1 | 8.0 | 1 | 13.6 |  | 0.0 |
| Senegal | 19 | 2.3 | 12 | 3.4 | 7 | 1.4 |

| Sierra Leone | 8 | 2.4 | 6 | 3.4 | 2 | 1.2 |
| --- | --- | --- | --- | --- | --- | --- |
| Somalia | 29 | 3.8 | 20 | 5.4 | 9 | 2.2 |
| South Africa | 145 | 2.6 | 73 | 3.4 | 72 | 2.5 |
| South Sudan | 23 | 4.2 | 16 | 5.7 | 7 | 2.2 |
| Tanzania, United Republic of | 105 | 3.8 | 56 | 5.1 | 49 | 3.2 |
| The Republic of the Gambia | 2 | 1.5 | 1 | 2.0 | 1 | 0.72 |
| Togo | 22 | 6.4 | 16 | 9.4 | 6 | 2.4 |
| Uganda | 165 | 9.1 | 104 | 15.1 | 61 | 4.3 |
| Zambia | 38 | 4.6 | 21 | 6.2 | 17 | 3.3 |
| Zimbabwe | 58 | 6.4 | 28 | 10.5 | 30 | 6.3 |

# *ASR: per 1,000,000 persons

**Table 1b.** Global incidence of nasal cancer by age

|  | **Young** |  | **Old** |  |
| --- | --- | --- | --- | --- |
| **Region** |  |  |  |  |
|  | **New cases** | **ASR** | **New cases** | **ASR** |
| **World** | 8,847 | 2.4 | 20,113 | 13.4 |
| **Asia** | 6,006 | 2.6 | 12,589 | 13.6 |
| **Eastern Asia** | 2,017 | 2.5 | 5,671 | 12.9 |
| China | 1,754 | 1.9 | 4,494 | 10.8 |
| Japan | 161 | 2.2 | 888 | 18.4 |
| Korea, Democratic Republic of | 55 | 3.5 | 98 | 15.9 |
| Korea, Republic of | 46 | 1.3 | 185 | 10.9 |
| Mongolia | 1 | 0.43 | 6 | 14.3 |
| **South-Eastern Asia** | 1,315 | 3.9 | 1,885 | 15.6 |
| Brunei Darussalam | 1 | 1.9 | 1 | 15.5 |
| Cambodia | 21 | 3.0 | 27 | 11.6 |
| Indonesia | 539 | 3.5 | 674 | 13.8 |
| Lao People's Democratic Republic | 17 | 5.2 | 13 | 14.4 |
| Malaysia | 45 | 2.4 | 62 | 10.6 |
| Myanmar | 217 | 7.3 | 236 | 24.4 |
| Philippines | 170 | 3.3 | 335 | 20.8 |
| Singapore | 4 | 0.89 | 27 | 13.6 |
| Thailand | 137 | 3.1 | 266 | 13.1 |
| Timor-Leste |  | 0.0 | 1 | 6.8 |
| Viet Nam | 164 | 3.0 | 243 | 12.3 |
| **South-Central Asia** | 2,473 | 2.5 | 4,636 | 14.6 |
| Afghanistan | 20 | 1.5 | 23 | 7.3 |
| Bangladesh | 412 | 4.9 | 468 | 20.3 |
| Bhutan | 1 | 3.0 | 1 | 6.4 |
| India | 1,588 | 2.3 | 3,459 | 14.7 |
| Iran, Islamic Republic of | 55 | 1.2 | 66 | 4.5 |
| Kazakhstan | 22 | 2.3 | 52 | 13.8 |
| Kyrgyzstan | 1 | 0.49 | 7 | 7.7 |
| Maldives | 0 | 1.4 | 1 | 9.9 |
| Nepal | 38 | 3.1 | 36 | 8.4 |

| Pakistan | 286 | 3.2 | 414 | 15.9 |
| --- | --- | --- | --- | --- |
| Sri Lanka | 26 | 2.3 | 83 | 15.4 |
| Tajikistan | 3 | 0.85 | 2 | 2.3 |
| Turkmenistan | 5 | 1.6 | 7 | 7.7 |
| Uzbekistan | 16 | 0.94 | 17 | 3.4 |
| **Western Asia** | 201 | 1.4 | 397 | 10.0 |
| Armenia | 3 | 1.8 | 9 | 11.0 |
| Azerbaijan | 9 | 1.6 | 21 | 9.7 |
| Bahrain | 1 | 0.67 | 1 | 3.8 |
| Gaza Strip and West Bank | 4 | 1.9 | 4 | 8.9 |
| Georgia | 6 | 2.7 | 17 | 14.4 |
| Iraq | 12 | 0.72 | 28 | 7.8 |
| Israel | 8 | 2.0 | 19 | 9.8 |
| Jordan | 8 | 1.8 | 7 | 6.2 |
| Kuwait | 2 | 0.44 | 3 | 5.7 |
| Lebanon | 3 | 0.76 | 10 | 7.6 |
| Oman | 4 | 1.2 | 2 | 3.7 |
| Qatar | 1 | 0.33 | 1 | 4.8 |
| Saudi Arabia | 27 | 1.1 | 43 | 10.2 |
| Syrian Arab Republic | 10 | 1.3 | 18 | 7.8 |
| Turkey | 83 | 1.8 | 193 | 11.4 |
| United Arab Emirates | 2 | 0.25 | 3 | 3.8 |
| Yemen | 18 | 1.8 | 18 | 7.0 |
| **Oceania** | 57 | 2.7 | 128 | 12.9 |
| Australia | 25 | 1.9 | 87 | 12.0 |
| Fiji | 5 | 11.2 | 2 | 10.3 |
| France, New Caledonia |  | 0.0 | 2 | 28.6 |
| French Polynesia | 1 | 6.0 | 1 | 23.7 |
| Guam |  | 0.0 | 0 | 11.3 |
| New Zealand | 6 | 2.3 | 15 | 10.8 |
| Papua New Guinea | 18 | 4.4 | 21 | 20.4 |
| Samoa |  | 0.0 |  | 0.0 |
| Solomon Islands | 2 | 7.3 | 0 | 8.2 |

| Vanuatu |  | 0.0 | 0 | 16.7 |
| --- | --- | --- | --- | --- |
| **Northern America** | 334 | 1.8 | 1,387 | 12.3 |
| Canada | 34 | 1.7 | 121 | 9.6 |
| United States of America | 300 | 1.8 | 1,266 | 12.7 |
| **Latin America and the Caribbean** | 413 | 1.6 | 1,014 | 11.8 |
| **Central America & Caribbean** | 78 | 1.2 | 193 | 8.9 |
| Bahamas | 0 | 1.9 | 1 | 6.4 |
| Barbados |  | 0.0 | 0 | 4.3 |
| Belize |  | 0.0 | 0 | 4.2 |
| Costa Rica | 5 | 1.7 | 7 | 6.0 |
| Cuba | 1 | 1.3 | 5 | 15.0 |
| Dominican Republic | 16 | 3.0 | 26 | 14.2 |
| El Salvador | 1 | 0.30 | 3 | 2.3 |
| France, Guadeloupe | 0 | 1.9 | 1 | 10.1 |
| France, Martinique | 0 | 0.48 | 1 | 8.0 |
| Guatemala | 4 | 0.51 | 6 | 3.1 |
| Haiti | 6 | 1.1 | 6 | 4.1 |
| Honduras | 7 | 1.7 | 23 | 19.6 |
| Jamaica | 2 | 1.1 | 11 | 17.7 |
| Mexico | 29 | 0.43 | 85 | 3.8 |
| Nicaragua | 3 | 0.90 | 5 | 5.5 |
| Panama | 0 | 0.21 | 4 | 4.7 |
| Puerto Rico | 3 | 2.1 | 7 | 7.6 |
| Saint Lucia |  | 0.0 | 0 | 6.4 |
| Trinidad and Tobago | 1 | 1.5 | 2 | 5.7 |
| **South America** | 335 | 1.9 | 821 | 13.1 |
| Argentina | 22 | 1.0 | 93 | 9.8 |
| Bolivia, Plurinational State of | 10 | 1.7 | 9 | 5.0 |
| Brazil | 160 | 1.4 | 380 | 8.2 |
| Chile | 6 | 0.61 | 54 | 10.9 |
| Colombia | 61 | 2.3 | 120 | 11.5 |
| Ecuador | 7 | 0.78 | 19 | 6.6 |
| French Guiana | 0 | 2.0 | 2 | 38.1 |

| Guyana | 1 | 3.6 | 1 | 7.8 |
| --- | --- | --- | --- | --- |
| Paraguay | 6 | 1.9 | 10 | 9.5 |
| Peru | 30 | 1.7 | 45 | 7.0 |
| Suriname |  | 0.0 | 0 | 3.0 |
| Uruguay | 1 | 0.60 | 12 | 14.1 |
| Venezuela, Bolivarian Republic of | 31 | 2.1 | 76 | 13.6 |
| **Europe** | 902 | 2.1 | 3,558 | 15.1 |
| **Northern Europe** | 108 | 2.0 | 432 | 13.5 |
| Denmark | 9 | 2.9 | 37 | 19.7 |
| Estonia | 1 | 1.1 | 7 | 18.3 |
| Finland | 1 | 0.32 | 15 | 7.1 |
| Iceland |  | 0.0 | 1 | 13.1 |
| Ireland | 6 | 1.9 | 17 | 12.6 |
| Latvia | 3 | 2.6 | 13 | 21.4 |
| Lithuania | 3 | 2.2 | 15 | 16.5 |
| Norway | 6 | 1.8 | 23 | 13.7 |
| Sweden | 2 | 0.45 | 17 | 5.0 |
| United Kingdom | 77 | 2.2 | 287 | 13.8 |
| **Western Europe** | 131 | 2.0 | 1,066 | 17.1 |
| Austria | 9 | 1.9 | 39 | 13.3 |
| Belgium | 11 | 1.8 | 74 | 20.0 |
| France | 72 | 2.1 | 339 | 16.7 |
| Germany | 21 | 0.49 | 475 | 16.5 |
| Luxembourg | 0 | 1.2 | 4 | 23.9 |
| Switzerland | 4 | 0.88 | 31 | 11.0 |
| The Netherlands | 14 | 1.5 | 104 | 17.3 |
| **Southern Europe** | 202 | 1.7 | 702 | 11.9 |
| Albania | 4 | 2.9 | 9 | 10.8 |
| Bosnia and Herzegovina | 6 | 3.6 | 19 | 16.9 |
| Croatia | 37 | 5.5 | 105 | 29.6 |
| Cyprus | 12 | 1.7 | 13 | 3.7 |
| Greece | 14 | 2.2 | 48 | 14.0 |
| Italy | 66 | 1.8 | 215 | 10.2 |

| Malta |  | 0.0 | 3 | 16.4 |
| --- | --- | --- | --- | --- |
| Montenegro | 2 | 5.4 | 5 | 27.8 |
| North Macedonia | 7 | 5.4 | 38 | 62.0 |
| Portugal | 9 | 1.4 | 36 | 10.8 |
| Serbia | 15 | 3.1 | 54 | 18.9 |
| Slovenia | 2 | 1.8 | 9 | 13.0 |
| Spain | 28 | 0.90 | 148 | 10.0 |
| **Central and Eastern Europe** | 461 | 2.6 | 1,358 | 16.2 |
| Belarus | 15 | 2.9 | 47 | 15.9 |
| Bulgaria | 6 | 1.3 | 32 | 13.0 |
| Czechia | 5 | 0.46 | 7 | 3.4 |
| Hungary | 15 | 2.5 | 69 | 22.3 |
| Poland | 46 | 2.1 | 159 | 13.1 |
| Republic of Moldova | 10 | 4.7 | 30 | 25.6 |
| Romania | 65 | 5.5 | 150 | 24.6 |
| Russian Federation | 220 | 2.7 | 572 | 12.8 |
| Slovakia | 10 | 2.9 | 22 | 13.0 |
| Ukraine | 69 | 2.7 | 270 | 19.8 |
| **Africa** | 1,135 | 2.2 | 1,437 | 11.0 |
| **Northern Africa** | 140 | 1.3 | 556 | 15.4 |
| Algeria | 23 | 1.0 | 104 | 15.1 |
| Egypt | 55 | 1.2 | 192 | 13.2 |
| Libya | 5 | 1.2 | 18 | 20.7 |
| Morocco | 34 | 1.9 | 158 | 22.2 |
| Sudan | 14 | 0.90 | 33 | 7.3 |
| Tunisia | 9 | 1.4 | 51 | 20.2 |
| **Sub-Saharan Africa** | 995 | 2.5 | 881 | 9.3 |
| Angola | 28 | 2.6 | 38 | 16.8 |
| Benin | 4 | 0.72 | 2 | 1.8 |
| Botswana | 3 | 3.4 | 5 | 17.7 |
| Burkina Faso | 27 | 4.2 | 5 | 3.7 |
| Burundi | 13 | 3.2 | 9 | 10.7 |
| Cabo Verde | 4 | 15.4 | 3 | 43.2 |

| Cameroon 13 1.5 6 2.8 | | | |
| --- | --- | --- | --- |
| Central African Republic 1 | 0.82 | 2 | 4.7 |
| Chad 10 | 2.3 | 8 | 6.8 |
| Comoros | 0.0 |  | 0.0 |
| Congo, Democratic Republic of 38 | 1.2 | 42 | 5.8 |
| Congo, Republic of | 0.0 | 2 | 4.2 |
| Côte d'Ivoire 17 | 7.8 | 53 | 37.8 |
| Djibouti 1 | 2.4 | 1 | 8.9 |
| Equatorial Guinea 2 | 4.5 | 1 | 13.3 |
| Eritrea 3 | 2.7 | 3 | 7.7 |
| Eswatini | 0.0 | 0 | 2.3 |
| Ethiopia 150 | 3.5 | 71 | 6.8 |
| France, La Réunion 2 | 4.3 | 7 | 28.9 |
| Gabon 6 | 6.0 | 6 | 27.5 |
| Ghana 45 | 3.4 | 30 | 9.3 |
| Guinea 11 | 2.8 | 10 | 8.9 |
| Guinea-Bissau | 0.0 | 1 | 7.2 |
| Kenya 29 | 1.4 | 87 | 20.1 |
| Lesotho 2 | 2.5 | 2 | 7.9 |
| Liberia 4 | 2.2 | 4 | 7.9 |
| Madagascar 24 | 1.8 | 14 | 5.8 |
| Malawi 16 | 2.3 | 9 | 6.8 |
| Mali 12 | 1.6 | 11 | 7.9 |
| Mauritania 3 | 1.6 | 2 | 5.6 |
| Mauritius 3 | 4.3 | 7 | 20.3 |
| Mozambique 23 | 1.4 | 11 | 4.4 |
| Namibia 1 | 1.1 | 7 | 28.2 |
| Niger 22 | 3.1 | 9 | 5.4 |
| Nigeria 108 | 1.5 | 140 | 7.9 |
| Rwanda 13 | 2.6 | 16 | 12.6 |
| Sao Tome and Principe | 0.0 | 0 | 14.9 |
| Senegal 11 | 1.8 | 9 | 6.5 |
| Sierra Leone 4 | 1.4 | 4 | 6.8 |

| Somalia | 15 | 3.1 | 14 | 10.5 |
| --- | --- | --- | --- | --- |
| South Africa | 61 | 2.1 | 92 | 10.5 |
| South Sudan | 10 | 2.8 | 11 | 10.7 |
| Tanzania, United Republic of | 57 | 2.4 | 31 | 6.8 |
| The Republic of the Gambia | 2 | 2.4 | 0 | 2.6 |
| Togo | 8 | 2.4 | 9 | 13.1 |
| Uganda | 151 | 10.1 | 54 | 19.3 |
| Zambia | 20 | 3.0 | 13 | 11.2 |
| Zimbabwe | 18 | 3.7 | 20 | 16.9 |

# *ASR: per 1,000,000 persons

**Supplementary Table 2.** Results of risk factors associations with nasal cancer

|  |  |  | **Overall** |  |  |
| --- | --- | --- | --- | --- | --- |
| **Outcome** | **Risk factor** |  |  |  |  |
|  |  | ***β*** | ***95% CI*** |  | ***P*** |
|  | HDI | 0.254 | 0.022 | 0.487 | 0.032* |
|  | GDP per capita | 0.098 | -0.082 | 0.278 | 0.284 |
|  | Smoking | 0.086 | 0.024 | 0.149 | 0.007* |
|  | Alcohol drinking | 0.091 | 0.024 | 0.159 | 0.008* |
| **All Sexes and ages** | Dietary | 0.042 | 0.011 | 0.074 | 0.009* |
|  | Physical inactivity | -0.133 | -0.246 | -0.021 | 0.021* |
|  | Obesity | -0.010 | -0.042 | 0.023 | 0.552 |
|  | Hypertension | 0.071 | 0.031 | 0.111 | 0.001* |
|  | Diabetes | 0.001 | -0.066 | 0.068 | 0.974 |
|  | Lipid | 0.008 | -0.022 | 0.039 | 0.582 |
|  |  |  |  |  |  |
|  | HDI | 0.365 | -0.010 | 0.740 | 0.056 |
|  | GDP per capita | 0.205 | -0.082 | 0.493 | 0.160 |
|  | Smoking | 0.085 | 0.013 | 0.157 | 0.022* |
|  | Alcohol drinking | 0.122 | 0.046 | 0.199 | 0.002* |
| **Male** | Dietary | 0.056 | 0.017 | 0.095 | 0.005* |
|  | Physical inactivity | -0.235 | -0.414 | -0.055 | 0.011* |
|  | Obesity | -0.020 | -0.072 | 0.032 | 0.458 |
|  | Hypertension | 0.106 | 0.046 | 0.166 | 0.001* |
|  | Diabetes | -0.015 | -0.115 | 0.086 | 0.776 |
|  | Lipid | -0.0004 | -0.047 | 0.047 | 0.988 |
|  |  |  |  |  |  |
|  | HDI | 0.155 | -0.057 | 0.367 | 0.151 |
|  | GDP per capita | -0.068 | -0.228 | 0.091 | 0.399 |
|  | Smoking | 0.064 | 0.002 | 0.125 | 0.042* |
|  | Alcohol drinking | 0.009 | -0.078 | 0.095 | 0.844 |

| **Female** | Dietary | 0.005 | -0.031 | 0.040 | 0.789 |
| --- | --- | --- | --- | --- | --- |
|  | Physical inactivity | 0.013 | -0.085 | 0.112 | 0.789 |
|  | Obesity | 0.016 | -0.014 | 0.046 | 0.305 |
|  | Hypertension | 0.028 | -0.009 | 0.064 | 0.133 |
|  | Diabetes | 0.099 | 0.032 | 0.165 | 0.004* |
|  | Lipid | 0.014 | -0.014 | 0.042 | 0.325 |
|  |  |  |  |  |  |
|  | HDI | -0.109 | -0.307 | 0.088 | 0.277 |
|  | GDP per capita | -0.118 | -0.270 | 0.034 | 0.128 |
|  | Smoking | -0.013 | -0.073 | 0.047 | 0.673 |
|  | Alcohol drinking | 0.002 | -0.054 | 0.057 | 0.952 |
| **Young** | Dietary | 0.003 | -0.025 | 0.030 | 0.833 |
|  | Physical inactivity | -0.162 | -0.261 | -0.064 | 0.001* |
|  | Obesity | -0.028 | -0.055 | -0.0005 | 0.046* |
|  | Hypertension | 0.020 | -0.032 | 0.071 | 0.450 |
|  | Diabetes | 0.098 | -0.042 | 0.239 | 0.169 |
|  | Lipid | -0.037 | -0.067 | -0.008 | 0.013* |
|  |  |  |  |  |  |
|  | HDI | 1.342 | 0.602 | 2.082 | <0.001* |
|  | GDP per capita | 0.755 | 0.182 | 1.329 | 0.010* |
|  | Smoking | 0.443 | 0.278 | 0.609 | <0.001* |
|  | Alcohol drinking | 0.413 | 0.191 | 0.636 | <0.001* |
| **Old** | Dietary | 0.138 | 0.048 | 0.228 | 0.003* |
|  | Physical inactivity | -0.296 | -0.648 | 0.056 | 0.099 |
|  | Obesity | 0.025 | -0.059 | 0.110 | 0.553 |
|  | Hypertension | 0.131 | 0.025 | 0.237 | 0.016* |
|  | Diabetes | -0.025 | -0.145 | 0.095 | 0.683 |
|  | Lipid | 0.110 | 0.012 | 0.207 | 0.028* |

The analysis was conducted using univariable linear regression model at a country level.

*β*, beta coefficient. The beta coefficient can be interpreted as the change in incidence or mortality associated

with one percent increase of a certain risk factor.

CI, confidence interval; ASR, age-standardized rate; HDI, human development index; GDP, gross domestic products.

* *p* values less than 0.05.

**Supplementary Table 3.** Results of Joinpoint regression for trend analysis

1. Male

| **Region** | **AAPC** | **Lower CI** | **Upper CI** | **p-value** | **Significant** |
| --- | --- | --- | --- | --- | --- |
| ***Asia*** |  |  |  |  |  |
| Bahrain | -6.88 | -11.95 | -1.52 | 0.019 | * |
| China | -4.44 | -9.33 | 0.72 | 0.081 |  |
| India | -2.37 | -12.54 | 8.98 | 0.629 |  |
| Israel | 1.16 | -2.45 | 4.90 | 0.486 |  |
| Japan | 2.42 | 0.08 | 4.81 | 0.044 | * |
| Korea | -1.17 | -4.68 | 2.47 | 0.476 |  |
| Kuwait | 0.40 | -12.18 | 14.78 | 0.947 |  |
| Philippines | -7.19 | -15.13 | 1.48 | 0.090 |  |
| Thailand | -7.27 | -15.29 | 1.51 | 0.090 |  |
| Turkey | -0.72 | -10.91 | 10.64 | 0.881 |  |
| ***Oceania*** |  |  |  |  |  |
| Australia | 0.12 | -2.85 | 3.19 | 0.928 |  |
| New Zealand | 3.46 | -1.98 | 9.21 | 0.184 |  |
| ***Northern America*** |  |  |  |  |  |
| Canada | -0.13 | -2.69 | 2.51 | 0.915 |  |
| USA | 2.22 | -0.95 | 5.48 | 0.147 |  |
| ***Southern America*** |  |  |  |  |  |
| Brazil | 1.08 | -13.71 | 18.40 | 0.880 |  |
| Chile | 14.03 | 9.91 | 18.30 | <0.001 | * |
| Colombia | -3.16 | -12.14 | 6.73 | 0.468 |  |
| Ecuador | 3.04 | -12.09 | 20.79 | 0.675 |  |
| Martinique | -3.35 | -12.97 | 7.35 | 0.476 |  |
| ***Northern Europe*** |  |  |  |  |  |

| Denmark | 0.00 | -3.53 | 3.66 | 0.998 |  |
| --- | --- | --- | --- | --- | --- |
| Estonia | -0.48 | -13.55 | 14.57 | 0.939 |  |
| Iceland | 0.71 | -12.76 | 16.27 | 0.912 |  |
| Ireland | -3.14 | -8.65 | 2.70 | 0.244 |  |
| Lithuania | 0.11 | -6.55 | 7.23 | 0.973 |  |
| Norway | 0.83 | -4.97 | 6.99 | 0.756 |  |
| United Kingdom | 2.14 | 0.33 | 3.99 | 0.026 | * |
| ***Western Europe*** |  |  |  |  |  |
| Austria | 1.92 | -4.81 | 9.14 | 0.539 |  |
| France | -4.29 | -8.48 | 0.10 | 0.054 |  |
| Germany | 1.68 | -4.42 | 8.17 | 0.552 |  |
| Netherlands | -2.61 | -5.54 | 0.42 | 0.082 |  |
| Switzerland | 3.62 | -7.89 | 16.56 | 0.506 |  |
| ***Southern Europe*** |  |  |  |  |  |
| Croatia | 5.26 | -3.00 | 14.21 | 0.219 |  |
| Cyprus | 7.48 | -8.85 | 26.75 | 0.342 |  |
| Italy | 5.65 | -3.21 | 15.33 | 0.186 |  |
| Malta | 0.44 | -11.73 | 14.29 | 0.939 |  |
| Slovenia | -1.73 | -9.03 | 6.14 | 0.615 |  |
| Spain | 1.48 | -3.67 | 6.90 | 0.533 |  |
| ***Eastern Europe*** |  |  |  |  |  |
| Bulgaria | 2.07 | -2.11 | 6.42 | 0.292 |  |
| Czech Republic | 1.27 | -2.21 | 4.88 | 0.430 |  |
| Poland | -9.70 | -19.72 | 1.56 | 0.080 |  |
| ***Africa*** |  |  |  |  |  |
| Uganda | 3.93 | -20.38 | 35.65 | 0.747 |  |

AAPC, annual percentage change; CI, confidence interval; * p values less than 0·05.

1. Female

| **Region** | **AAPC** | **Lower CI** | **Upper CI** | **p-value** | **Significant** |
| --- | --- | --- | --- | --- | --- |
| ***Asia*** |  |  |  |  |  |
| Bahrain | -13.17 | -24.36 | -0.34 | 0.045 | * |
| China | -3.06 | -6.42 | 0.43 | 0.077 |  |
| India | -4.06 | -16.00 | 9.58 | 0.493 |  |
| Israel | 3.06 | -5.81 | 12.76 | 0.463 |  |
| Japan | 3.00 | -1.45 | 7.66 | 0.161 |  |
| Korea | -3.70 | -11.59 | 4.90 | 0.388 |  |
| Kuwait | 12.71 | 6.88 | 18.87 | 0.001 | * |
| Philippines | -1.00 | -9.90 | 8.78 | 0.812 |  |
| Thailand | -4.38 | -13.93 | 6.23 | 0.355 |  |
| Turkey | -2.12 | -10.27 | 6.76 | 0.584 |  |
| ***Oceania*** |  |  |  |  |  |
| Australia | 2.11 | -1.50 | 5.85 | 0.218 |  |
| New Zealand | 1.55 | -12.54 | 17.91 | 0.818 |  |
| ***Northern America*** |  |  |  |  |  |
| Canada | 0.33 | -2.76 | 3.53 | 0.812 |  |
| USA | -2.33 | -4.26 | -0.36 | 0.026 | * |
| ***Southern America*** |  |  |  |  |  |
| Brazil | -6.18 | -20.38 | 10.56 | 0.397 |  |
| Chile | -0.19 | -6.92 | 7.02 | 0.951 |  |
| Colombia | -15.02 | -31.38 | 5.23 | 0.136 |  |
| Ecuador | -0.03 | -15.44 | 18.19 | 0.997 |  |
| Martinique | -1.21 | -14.18 | 13.72 | 0.847 |  |
| ***Northern Europe*** |  |  |  |  |  |
| Denmark | 2.63 | -2.49 | 8.01 | 0.275 |  |

| Estonia | 16.01 | -6.69 | 44.24 | 0.154 |  |
| --- | --- | --- | --- | --- | --- |
| Iceland | -14.68 | -23.50 | -4.84 | 0.010 | * |
| Ireland | 0.74 | -6.40 | 8.43 | 0.823 |  |
| Lithuania | 1.11 | -9.80 | 13.34 | 0.829 |  |
| Norway | 0.64 | -6.63 | 8.46 | 0.850 |  |
| United Kingdom | 2.59 | 1.46 | 3.73 | 0.001 | * |
| ***Western Europe*** |  |  |  |  |  |
| Austria | 1.67 | -3.99 | 7.66 | 0.524 |  |
| France | -0.52 | -4.63 | 3.77 | 0.783 |  |
| Germany | -1.27 | -8.13 | 6.10 | 0.693 |  |
| Netherlands | 4.56 | 1.26 | 7.98 | 0.013 | * |
| Switzerland | -5.55 | -15.93 | 6.11 | 0.291 |  |
| ***Southern Europe*** |  |  |  |  |  |
| Croatia | -2.26 | -9.59 | 5.67 | 0.518 |  |
| Cyprus | -4.23 | -12.55 | 4.88 | 0.351 |  |
| Italy | 4.81 | -4.30 | 14.78 | 0.268 |  |
| Malta | -12.86 | -19.78 | -5.34 | 0.005 | * |
| Slovenia | -1.48 | -13.21 | 11.84 | 0.793 |  |
| Spain | -1.55 | -9.25 | 6.81 | 0.670 |  |
| ***Eastern Europe*** |  |  |  |  |  |
| Bulgaria | 0.78 | -7.56 | 9.87 | 0.841 |  |
| Czech Republic | -4.89 | -10.09 | 0.61 | 0.080 |  |
| Poland | 0.35 | -13.49 | 16.40 | 0.958 |  |
| ***Africa*** |  |  |  |  |  |
| Uganda | -16.44 | -26.12 | -5.49 | 0.010 | * |

AAPC, annual percentage change; CI, confidence interval; * p values less than 0·05.

1. Both

| **Region** | **AAPC** | **Lower CI** | **Upper CI** | **p-value** | **Significant** |
| --- | --- | --- | --- | --- | --- |
| ***Asia*** |  |  |  |  |  |
| Bahrain | -10.14 | -16.76 | -3.00 | 0.012 | * |
| China | -4.06 | -7.53 | -0.45 | 0.032 | * |
| India | -3.23 | -11.62 | 5.95 | 0.427 |  |
| Israel | 1.81 | -3.01 | 6.88 | 0.418 |  |
| Japan | 2.45 | 1.05 | 3.88 | 0.004 | * |
| Korea | -1.99 | -4.91 | 1.01 | 0.163 |  |
| Kuwait | 10.57 | -2.84 | 25.83 | 0.111 |  |
| Philippines | -4.58 | -11.90 | 3.34 | 0.212 |  |
| Thailand | -6.13 | -11.70 | -0.22 | 0.044 | * |
| Turkey | -1.16 | -8.89 | 7.23 | 0.750 |  |
| ***Oceania*** |  |  |  |  |  |
| Australia | 0.87 | -1.62 | 3.42 | 0.447 |  |
| New Zealand | 1.77 | -4.86 | 8.86 | 0.565 |  |
| ***Northern America*** |  |  |  |  |  |
| Canada | 0.18 | -1.89 | 2.29 | 0.851 |  |
| USA | 0.46 | -1.89 | 2.86 | 0.666 |  |
| ***Southern America*** |  |  |  |  |  |
| Brazil | -2.36 | -12.74 | 9.26 | 0.638 |  |
| Chile | 3.29 | -5.66 | 13.08 | 0.434 |  |
| Colombia | -7.94 | -12.79 | -2.83 | 0.008 | * |
| Ecuador | 6.07 | -11.82 | 27.59 | 0.483 |  |
| Martinique | -3.44 | -14.49 | 9.04 | 0.526 |  |
| ***Northern Europe*** |  |  |  |  |  |
| Denmark | 1.21 | -2.53 | 5.09 | 0.482 |  |

| Estonia | 3.49 | -7.85 | 16.23 | 0.515 |  |
| --- | --- | --- | --- | --- | --- |
| Iceland | -6.56 | -15.58 | 3.43 | 0.162 |  |
| Ireland | -1.36 | -5.52 | 2.99 | 0.486 |  |
| Lithuania | 0.47 | -5.22 | 6.51 | 0.856 |  |
| Norway | 0.82 | -4.32 | 6.23 | 0.729 |  |
| United Kingdom | 2.37 | 1.06 | 3.69 | 0.003 | * |
| ***Western Europe*** |  |  |  |  |  |
| Austria | 1.76 | -3.37 | 7.17 | 0.458 |  |
| France | -3.23 | -7.03 | 0.73 | 0.095 |  |
| Germany | 0.59 | -5.09 | 6.61 | 0.822 |  |
| Netherlands | 0.10 | -1.83 | 2.07 | 0.908 |  |
| Switzerland | -0.52 | -8.67 | 8.35 | 0.891 |  |
| ***Southern Europe*** |  |  |  |  |  |
| Croatia | 2.77 | -0.97 | 6.65 | 0.127 |  |
| Cyprus | 9.65 | -9.10 | 32.25 | 0.290 |  |
| Italy | 5.33 | -2.75 | 14.08 | 0.172 |  |
| Malta | -6.65 | -21.54 | 11.06 | 0.387 |  |
| Slovenia | -1.51 | -8.78 | 6.33 | 0.658 |  |
| Spain | 0.01 | -3.69 | 3.85 | 0.995 |  |
| ***Eastern Europe*** |  |  |  |  |  |
| Bulgaria | 1.70 | -2.35 | 5.93 | 0.367 |  |
| Czech Republic | -0.33 | -3.53 | 2.96 | 0.819 |  |
| Poland | -6.23 | -13.06 | 1.14 | 0.086 |  |
| ***Africa*** |  |  |  |  |  |
| Uganda | -6.85 | -20.07 | 8.57 | 0.317 |  |

AAPC, annual percentage change; CI, confidence interval; * p values less than 0·05.

1. Young

| **Region** | **AAPC** | **Lower CI** | **Upper CI** | **p-value** | **Significant** |
| --- | --- | --- | --- | --- | --- |
| ***Asia*** |  |  |  |  |  |
| Bahrain | -7.48 | -12.68 | -1.98 | 0.015 | * |
| China | -3.58 | -10.54 | 3.93 | 0.295 |  |
| India | 0.33 | -8.06 | 9.49 | 0.933 |  |
| Israel | 0.91 | -9.13 | 12.06 | 0.846 |  |
| Japan | 5.65 | -0.33 | 11.98 | 0.061 |  |
| Korea | 2.18 | -2.01 | 6.55 | 0.269 |  |
| Kuwait | -4.80 | -14.04 | 5.44 | 0.346 |  |
| Philippines | -0.92 | -5.02 | 3.36 | 0.628 |  |
| Thailand | -5.36 | -18.72 | 10.19 | 0.428 |  |
| Turkey | 3.29 | -13.15 | 22.84 | 0.678 |  |
| ***Oceania*** |  |  |  |  |  |
| Australia | 2.78 | -1.67 | 7.44 | 0.191 |  |
| New Zealand | 6.19 | -5.87 | 19.79 | 0.329 |  |
| ***Northern America*** |  |  |  |  |  |
| Canada | 1.26 | -4.17 | 7.00 | 0.615 |  |
| USA | -0.41 | -3.16 | 2.41 | 0.742 |  |
| ***Southern America*** |  |  |  |  |  |
| Brazil | -0.74 | -11.67 | 11.54 | 0.887 |  |
| Chile | 12.63 | -0.71 | 27.78 | 0.065 |  |
| Colombia | 6.36 | -8.55 | 23.70 | 0.374 |  |
| Ecuador | -4.34 | -19.59 | 13.80 | 0.572 |  |
| Martinique | 7.47 | -1.34 | 17.06 | 0.099 |  |
| ***Northern Europe*** |  |  |  |  |  |
| Denmark | 7.18 | -2.60 | 17.94 | 0.133 |  |

| Estonia | 4.97 | -7.84 | 19.56 | 0.415 |  |
| --- | --- | --- | --- | --- | --- |
| Iceland | -4.18 | -13.91 | 6.64 | 0.434 |  |
| Ireland | -10.35 | -24.99 | 7.14 | 0.195 |  |
| Lithuania | 3.12 | -14.59 | 24.50 | 0.749 |  |
| Norway | 7.70 | -4.79 | 21.82 | 0.203 |  |
| United Kingdom | 3.43 | -0.36 | 7.37 | 0.071 |  |
| ***Western Europe*** |  |  |  |  |  |
| Austria | -0.56 | -11.13 | 11.26 | 0.911 |  |
| France | -4.50 | -11.66 | 3.24 | 0.210 |  |
| Germany | -2.82 | -11.98 | 7.29 | 0.524 |  |
| Netherlands | 2.07 | -6.10 | 10.95 | 0.588 |  |
| Switzerland | -13.34 | -29.92 | 7.16 | 0.159 |  |
| ***Southern Europe*** |  |  |  |  |  |
| Croatia | 14.00 | 2.37 | 26.95 | 0.023 | * |
| Cyprus | 13.59 | -3.82 | 34.15 | 0.133 |  |
| Italy | 13.79 | -11.39 | 46.13 | 0.311 |  |
| Malta | -4.43 | -14.23 | 6.49 | 0.411 |  |
| Slovenia | 4.36 | -10.41 | 21.57 | 0.537 |  |
| Spain | -1.99 | -9.25 | 5.85 | 0.564 |  |
| ***Eastern Europe*** |  |  |  |  |  |
| Bulgaria | 3.11 | -5.35 | 12.33 | 0.433 |  |
| Czech Republic | -0.13 | -5.59 | 5.64 | 0.958 |  |
| Poland | -10.79 | -19.39 | -1.26 | 0.032 | * |
| ***Africa*** |  |  |  |  |  |
| Uganda | 0.49 | -15.95 | 20.14 | 0.951 |  |

AAPC, annual percentage change; CI, confidence interval; * p values less than 0·05.

1. Old

| **Region** | **AAPC** | **Lower CI** | **Upper CI** | **p-value** | **Significant** |
| --- | --- | --- | --- | --- | --- |
| ***Asia*** |  |  |  |  |  |
| Bahrain | NA | NA | NA | NA | NA |
| China | -4.07 | -7.52 | -0.50 | 0.031 | * |
| India | -4.87 | -14.74 | 6.15 | 0.324 |  |
| Israel | 3.73 | -3.15 | 11.10 | 0.253 |  |
| Japan | 1.61 | -0.69 | 3.97 | 0.147 |  |
| Korea | -4.55 | -8.01 | -0.96 | 0.020 | * |
| Kuwait | 9.73 | -0.41 | 20.91 | 0.058 |  |
| Philippines | -5.12 | -13.70 | 4.31 | 0.237 |  |
| Thailand | -6.14 | -11.05 | -0.96 | 0.026 | * |
| Turkey | -0.24 | -10.24 | 10.88 | 0.959 |  |
| ***Oceania*** |  |  |  |  |  |
| Australia | -0.30 | -2.56 | 2.01 | 0.771 |  |
| New Zealand | -1.90 | -9.36 | 6.17 | 0.590 |  |
| ***Northern America*** |  |  |  |  |  |
| Canada | -0.05 | -3.28 | 3.29 | 0.975 |  |
| USA | 1.07 | -1.45 | 3.65 | 0.360 |  |
| ***Southern America*** |  |  |  |  |  |
| Brazil | 1.08 | -9.97 | 13.49 | 0.836 |  |
| Chile | 10.24 | 6.52 | 14.10 | <0.001 | * |
| Colombia | -15.26 | -22.98 | -6.77 | 0.004 | * |
| Ecuador | 6.64 | -8.54 | 24.35 | 0.362 |  |
| Martinique | -5.05 | -15.82 | 7.09 | 0.349 |  |
| ***Northern Europe*** |  |  |  |  |  |
| Denmark | 0.50 | -3.47 | 4.64 | 0.781 |  |

| Estonia | 4.92 | -6.46 | 17.69 | 0.363 |  |
| --- | --- | --- | --- | --- | --- |
| Iceland | -3.48 | -13.10 | 7.21 | 0.459 |  |
| Ireland | -0.88 | -6.92 | 5.57 | 0.756 |  |
| Lithuania | -0.11 | -5.36 | 5.42 | 0.963 |  |
| Norway | 0.12 | -4.54 | 5.00 | 0.956 |  |
| United Kingdom | 2.40 | 0.69 | 4.14 | 0.012 | * |
| ***Western Europe*** |  |  |  |  |  |
| Austria | 4.18 | -0.63 | 9.22 | 0.081 |  |
| France | -4.01 | -8.65 | 0.85 | 0.093 |  |
| Germany | 1.32 | -5.17 | 8.26 | 0.660 |  |
| Netherlands | -0.26 | -1.98 | 1.49 | 0.738 |  |
| Switzerland | 1.89 | -8.61 | 13.60 | 0.702 |  |
| ***Southern Europe*** |  |  |  |  |  |
| Croatia | -0.98 | -9.15 | 7.92 | 0.822 |  |
| Cyprus | 6.84 | 0.60 | 13.47 | 0.035 | * |
| Italy | 2.60 | -3.91 | 9.56 | 0.393 |  |
| Malta | -1.65 | -13.64 | 12.01 | 0.776 |  |
| Slovenia | -3.05 | -10.88 | 5.46 | 0.420 |  |
| Spain | -0.02 | -4.45 | 4.61 | 0.991 |  |
| ***Eastern Europe*** |  |  |  |  |  |
| Bulgaria | 3.39 | -3.17 | 10.40 | 0.274 |  |
| Czech Republic | -0.19 | -4.22 | 4.01 | 0.917 |  |
| Poland | -6.89 | -23.86 | 13.86 | 0.437 |  |
| ***Africa*** |  |  |  |  |  |
| Uganda | -9.90 | -22.13 | 4.25 | 0.138 |  |

AAPC, annual percentage change; CI, confidence interval; * p values less than 0·05.

NA, not available as it reported zero cases during the period and joinpoint regression could not be performed in such circumstances.

**Supplementary Table 4.** Multivariate analysis results for risk factors of nasal cancer

| **Outcome** | | | **Risk factor** | | | | **Overall** | | |
| --- | --- | --- | --- | --- | --- | --- | --- | --- | --- |
| ***β*** | | | ***95% CI*** | | | | ***P*** | | |
| HDI | | 0.030 | | -0.011 | | 0.071 | | 0.144 | |
| GDP per capital | | -0.003 | | -0.029 | | 0.024 | | 0.843 | |
| Smoking | | 0.008 | | -0.002 | | 0.018 | | 0.116 | |
| Alcohol drinking | | 0.002 | | -0.009 | | 0.012 | | 0.739 | |
| **Both Sexes**  **and**  **All Ages** | Dietary | | 0.001 | | -0.003 | | 0.005 | | 0.484 |
| Physical inactivity | | -0.008 | | -0.024 | | 0.009 | | 0.351 | |
| Obesity | | -0.001 | | -0.006 | | 0.003 | | 0.604 | |
| Hypertension | | 0.006 | | 0.001 | | 0.011 | | 0.015* | |
| Diabetes | | 0.000 | | -0.008 | | 0.009 | | 0.907 | |
| Lipid | | -0.006 | | -0.011 | | -0.000 | | 0.036* | |
| HDI | | 0.029 | | -0.037 | | 0.095 | | 0.385 | |
| GDP per capital | | 0.014 | | -0.029 | | 0.056 | | 0.523 | |
| Smoking | | 0.007 | | -0.003 | | 0.016 | | 0.183 | |
| Alcohol drinking | | 0.008 | | -0.004 | | 0.020 | | 0.203 | |
| **Male** | Dietary | | 0.003 | | -0.002 | | 0.008 | | 0.226 |
| Physical inactivity | | -0.002 | | -0.027 | | 0.023 | | 0.871 | |
| Obesity | | -0.002 | | -0.010 | | 0.005 | | 0.579 | |
| Hypertension | | 0.009 | | 0.001 | | 0.016 | | 0.022* | |
| Diabetes | | -0.000 | | -0.013 | | 0.012 | | 0.981 | |
| Lipid | | -0.012 | | -0.020 | | -0.004 | | 0.005* | |
| HDI | | 0.030 | | -0.007 | | 0.066 | | 0.109 | |
| GDP per capital | | -0.032 | | -0.056 | | -0.007 | | 0.012* | |
| Smoking | | 0.011 | | 0.000 | | 0.022 | | 0.041* | |
| Alcohol drinking | | -0.003 | | -0.017 | | 0.011 | | 0.693 | |
| Dietary | | -0.001 | | -0.005 | | 0.004 | | 0.739 | |
| Physical inactivity | | -0.000 | | -0.013 | | 0.013 | | 0.959 | |
| **Female** | Obesity | | -0.001 | | -0.005 | | 0.004 | | 0.791 |
| Hypertension | | 0.002 | | -0.002 | | 0.006 | | 0.414 | |

**Supplementary Figure 1.** Incidence trends for individual countries

Age-standard (World) incidence per 100 000

Age-standard (World) incidence per 100 000

Age-standard (World) incidence per 100 000

Age-standard (World) incidence per 100 000

Age-standard (World) incidence per 100 000

Age-standard (World) incidence per 100 000

|  | |  | **Asia** | |  |  | |
| --- | --- | --- | --- | --- | --- | --- | --- |
| Bahrain: Incidence | |  | China: Incidence | |  | 4  3  2  1  0 | India: Incidence  2003 2006 2009 2012  Male Female  Both Young (15-49)  Old (50-74) |
| 0.8 | |  | 2 | |  |  |  |
| 0.6 | |  | 1.5 | |  |  |  |
| 0.4 | |  | 1 | |  |  |  |
| 0.2 | |  | 0.5 | |  |  |  |
| 0 | |  | 0 | |  |  |  |
| 2003 2006 | 2009 2012 |  | 2003 2006 | 2009 2012 |  |  |  |
| Male | Female |  | Male | Female |  |  |  |
| Both | Young (15-49) |  | Both | Young (15-49) |  |  |  |
| Old (50-74) | |  | Old (50-74) | |  |  |  |
| Israel: Incidence | |  | Japan: Incidence | |  | Korea: Incidence | |
| 2 | |  | 2.5 | |  | 2.5 | |
| 1.5 | |  | 2 | |  | 2 | |
| 1 | |  | 1.5  1 | |  | 1.5  1 | |
| 0.5 | |  | 0.5 | |  | 0.5 | |
| 0 | |  | 0 | |  | 0 | |
| 2003 2006 | 2009 2012 |  | 2003 2006 | 2009 2012 |  | 2003 2006 2009 2012 | |
| Male | Female |  | Male | Female |  | Male Female | |
| Both  Old (50-74) | Young (15-49) |  | Both  Old (50-74) | Young (15-49) |  | Both Young (15-49)  Old (50-74) | |

Kuwait: Incidence

2.5

2

1.5

1

0.5

0

2003 2006

Male Both

2009 2012

Female Young (15-49)

Old (50-74)

Turkey: Incidence

2.5

2

1.5

1

0.5

0

2003 2006

Male Both

2009 2012

Female Young (15-49)

Old (50-74)

Philippines: Incidence

4

3

2

1

0

2003 2006

Male

Both

2009 2012

Female

Young (15-49)

Old (50-74)

Thailand: Incidence

2

1.5

1

0.5

0

2003

2006

2009

2012

Male

Both

Old (50-74)

Female

Young (15-49）

Age-standard (World) incidence per 100 000

Age-standard (World) incidence per 100 000

Age-standard (World) incidence per 100 000

Age-standard (World) incidence per 100 000

|  |  | **Oceania** |
| --- | --- | --- |
| Australia: Incidence  2  1.5  1  0.5  0  2003 2006 2009 2012  Male Female  Both Young (15-49)  Old (50-74) |  | New Zealand: Incidence  2.5  2  1.5  1  0.5  0  2003 2006 2009 2012  Male Female  Both Young (15-49)  Old (50-74) |
|  |  | **Northern America** |
| Canada: Incidence  2  1.5  1  0.5  0  2003 2006 2009 2012  Male Female  Both Young (15-49)  Old (50-74) |  | USA: Incidence  2  1.5  1  0.5  0  2003 2006 2009 2012  Male Female  Both Young (15-49)  Old (50-74) |

Age-standard (World) incidence per 100 000

Age-standard (World) incidence per 100 000

Age-standard (World) incidence per 100 000

Age-standard (World) incidence per 100 000

|  |  | **Southern America** |  |  |
| --- | --- | --- | --- | --- |
| Brazil: Incidence  3  2  1  0  2003 2006 2009 2012  Male Female  Both Young (15-49)  Old (50-74) |  | Chile: Incidence  1.5  1  0.5  0  2003 2006 2009 2012  Male Female  Both Young (15-49)  Old (50-74) |  | Colombia: Incidence  5  4  3  2  1  0  2003 2006 2009 2012  Male Female  Both Young (15-49)  Old (50-74) |
| Ecuador: Incidence  3  2  1  0  2003 2006 2009 2012  Male Female  Both Young (15-49)  Old (50-74) |  | Martinique: Incidence  4  3  2  1  0  2003 2006 2009 2012  Male Female  Both Young (15-49)  Old (50-74) |  |  |

Age-standard (World) incidence per 100 000

Age-standard (World) incidence per 100 000

Age-standard (World) incidence per 100 000

Age-standard (World) incidence per 100 000

Age-standard (World) incidence per 100 000

**Northern Europe**

Denmark: Incidence

4

3

2

1

0

2003

2006

Male Both

Old (50-74)

2009 2012

Female Young (15-49)

Ireland: Incidence

3

2

1

0

2003

2006

Male Both

Old (50-74)

2009 2012

Female Young (15-49)

United Kingdom: Incidence

2

1.5

1

0.5

0

2003 2006

Male

Both

2009 2012

Female

Young (15-49)

Old (50-74)

Estonia: Incidence

4

3

2

1

0

2003

2006

Male Both

Old (50-74)

2009 2012

Female Young (15-49)

Lithuania: Incidence

3

2

1

0

2003

2006

2009 2012

Female

Male

Both

Old (50-74)

Young (15-49)

Iceland: Incidence

5

4

3

2

1

0

2003 2006

Male

Both

2009 2012

Female

Young (15-49)

Old (50-74)

Norway: Incidence

3

2

1

0

2003

2006

2009 2012

Female

Male

Both

Old (50-74)

Young (15-49)

Age-standard (World) incidence per 100 000

Age-standard (World) incidence per 100 000

Age-standard (World) incidence per 100 000

Age-standard (World) incidence per 100

000

Age-standard (World) incidence per 100 000

Age-standard (World) incidence per 100 000

Age-standard (World) incidence per 100 000

|  |
| --- |
|  |
|  |
|  |

|  |
| --- |
|  |
|  |

|  |
| --- |
|  |
|  |

|  |
| --- |
|  |
|  |

|  |  | **Western Europe** |  |  |
| --- | --- | --- | --- | --- |
| Austria: Incidence  2.5  2  1.5  1  0.5  0  2003 2006 2009 2012  Male Female  Both Young (15-49)  Old (50-74) |  | France: Incidence  4  3  2  1  0  2003 2006 2009 2012  Male Female  Both Young (15-49)  Old (50-74) |  | Germany: Incidence  3  2  1  0  2003 2006 2009 2012  Male Female  Both Young (15-49)  Old (50-74) |
| Netherlands: Incidence  2.5  2  1.5  1  0.5  0  2003 2006 2009 2012  Male Female  Both Young (15-49)  Old (50-74) |  | Switzerland: Incidence  3  2  1  0  2003 2006 2009 2012  Male Female  Both Young (15-49)  Old (50-74) |  |  |

Age-standard (World) incidence per 100 000

Age-standard (World) incidence per 100 000

Age-standard (World) incidence per 100 000

Age-standard (World) incidence per 100 000

Age-standard (World) incidence per 100 000

Age-standard (World) incidence per 100 000

Age-standard (World) incidence per 100 000

Age-standard (World) incidence per 100 000

Age-standard (World) incidence per 100 000

Age-standard (World) incidence per 100 000

Age-standard (World) incidence per 100 000

|  |  | **Southern Europe** |  |  |
| --- | --- | --- | --- | --- |
| Croatia: Incidence |  | Cyprus: Incidence |  | Italy: Incidence  2  1.5  1  0.5  0  2003 2006 2009 2012  Male Female  Both Young (15-49)  Old (50-74） |
| 2.5 |  | 1 |  |  |
| 2 |  | 0.8 |  |  |
| 1.5 |  | 0.6 |  |  |
| 1 |  | 0.4 |  |  |
| 0.5 |  | 0.2 |  |  |
| 0 |  | 0 |  |  |
| 2003 2006 2009 2012 |  | 2003 2006 2009 2012 |  |  |
| Male Female |  | Male Female |  |  |
| Both Young (15-49) |  | Both Young (15-49) |  |  |
| Old (50-74) |  | Old (50-74) |  |  |
| Malta: Incidence  4  3  2  1  0  2003 2006 2009 2012  Male Female  Both Young (15-49)  Old (50-74) |  | Slovenia: Incidence  3  2  1  0  2003 2006 2009 2012  Male Female  Both Young (15-49)  Old (50-74) |  | Spain: Incidence  2  1.5  1  0.5  0  2003 2006 2009 2012  Male Female  Both Young (15-49)  Old (50-74) |

|  | | |  |  | **Eastern Europe** | |  | | | |
| --- | --- | --- | --- | --- | --- | --- | --- | --- | --- | --- |
| Bulgaria: Incidence | | |  |  | 2  1.5  1  0.5  0 | Czech Republic: Incidence  2003 2006 2009 2012  Male Female  Both Young (15-49)  Old (50-74) | Poland: Incidence | | | |
| 2.5 | | |  |  |  |  | 2.5 | | | |
| 2 | | |  |  |  |  | 2 | | | |
| 1.5 | | |  |  |  |  | 1.5 | | | |
| 1 | | |  |  |  |  | 1 | | | |
| 0.5 | | |  |  |  |  | 0.5 | | | |
| 0 | | |  |  |  |  | 0 | | | |
| 2003 | 2006 | 2009 2012 |  |  |  |  | 2003 | 2006 | 2009 | 2012 |
| Male |  | Female |  |  |  |  | Male |  |  | Female |
| Both |  | Young (15-49) |  |  |  |  | Both |  |  | Young (15-49) |
| Old (50-74) | | |  |  |  |  | Old (50-74) | | | |
|  | | |  |  | **Africa** | |  | | | |
| Uganda: Incidence | | | |  | | |  | | | |
| 6 | | | |  |  |  |  |  |  |  |
| 5 | | | |  |  |  |  |  |  |  |
| 4 | | | |  |  |  |  |  |  |  |
| 3 | | | |  |  |  |  |  |  |  |
| 2 | | | |  |  |  |  |  |  |  |
| 1 | | | |  |  |  |  |  |  |  |
| 0 | | | |  |  |  |  |  |  |  |
| 2003 | 2006 | 2009 2012 |  |  |  |  |  |  |  |  |
| Male |  | Female |  |  |  |  |  |  |  |  |
| Both |  | Young (15-49) |  |  |  |  |  |  |  |  |
| Old (50-74) | | | |  |  |  |  |  |  |  |

Age-standard (World) incidence per 100 000

Age-standard (World) incidence per 100 000

Age-standard (World) incidence per 100 000

Age-standard (World) incidence per 100 000

**Supplementary Figure 2.** Plots of Joinpoint regression for trend analysis

1. Male

| **Asia** | |
| --- | --- |
| 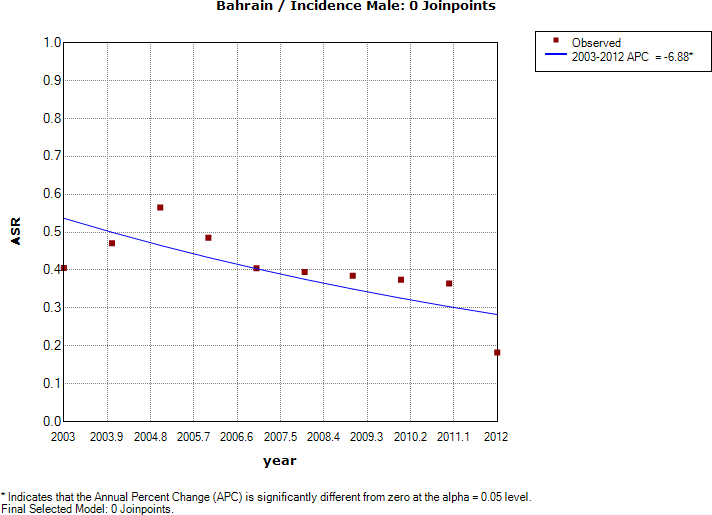 | 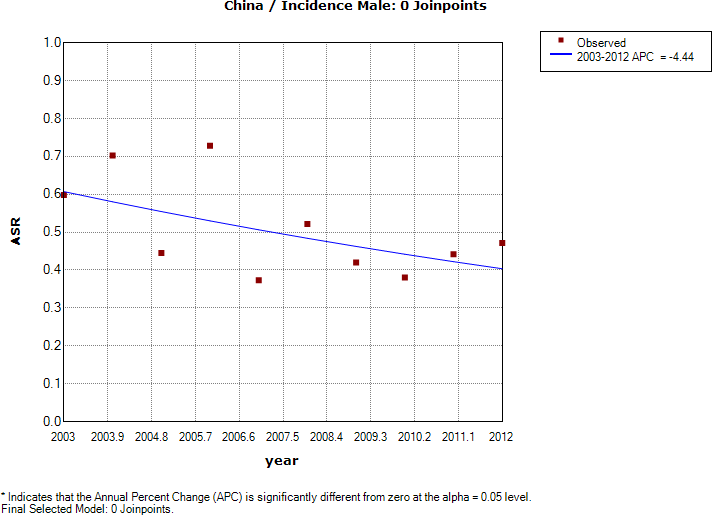 |
| 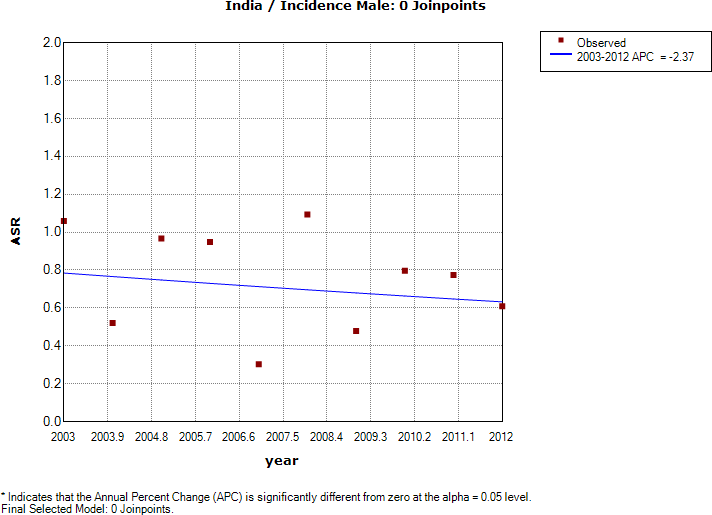 | 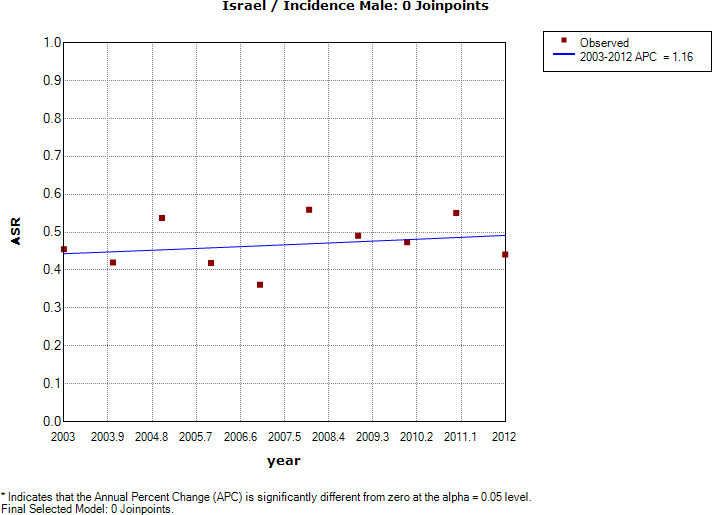 |
| 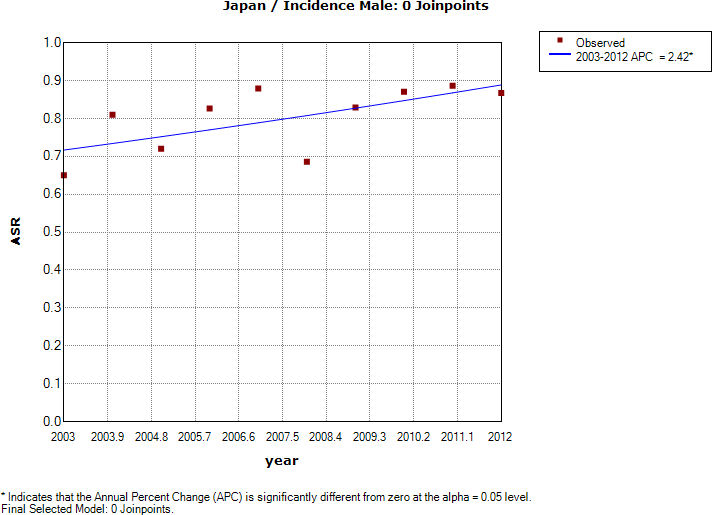 | 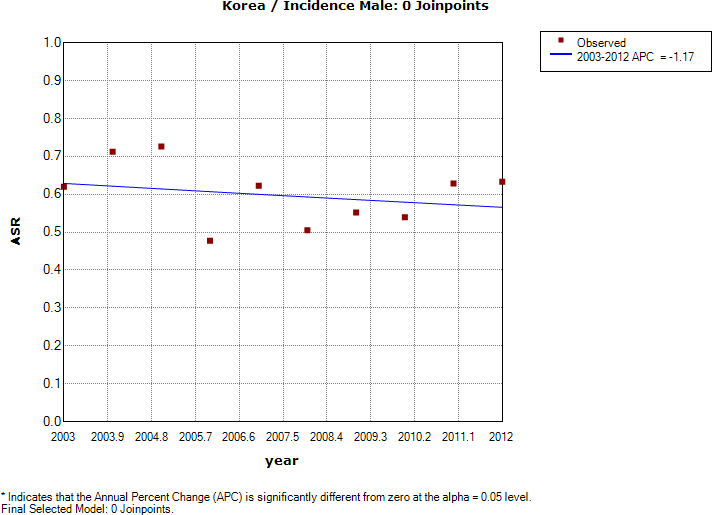 |

| 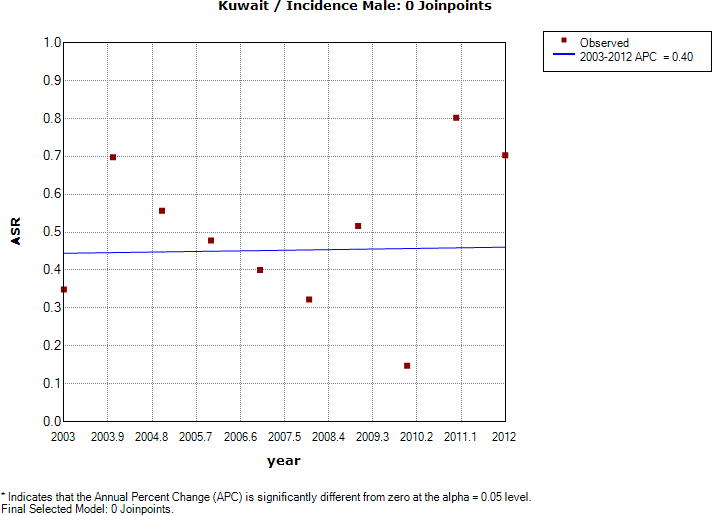 | 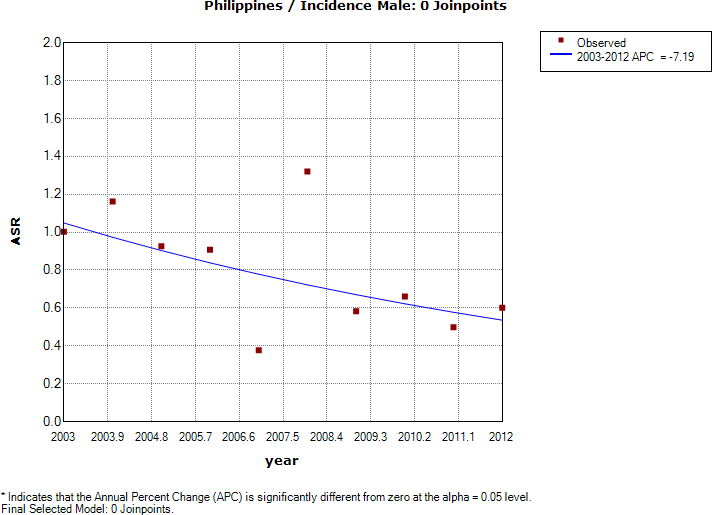 |
| --- | --- |
| 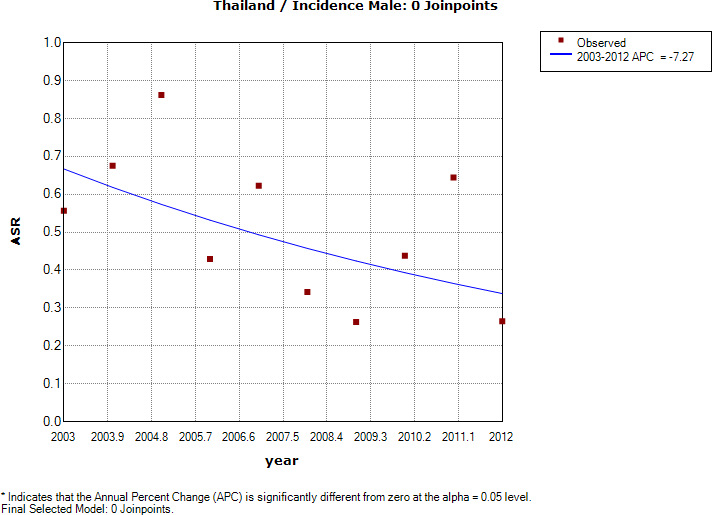 | 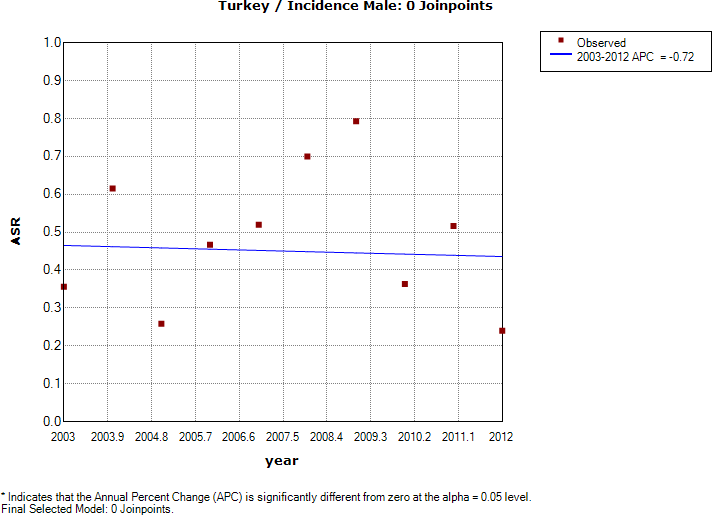 |
| **Oceania** | |
| 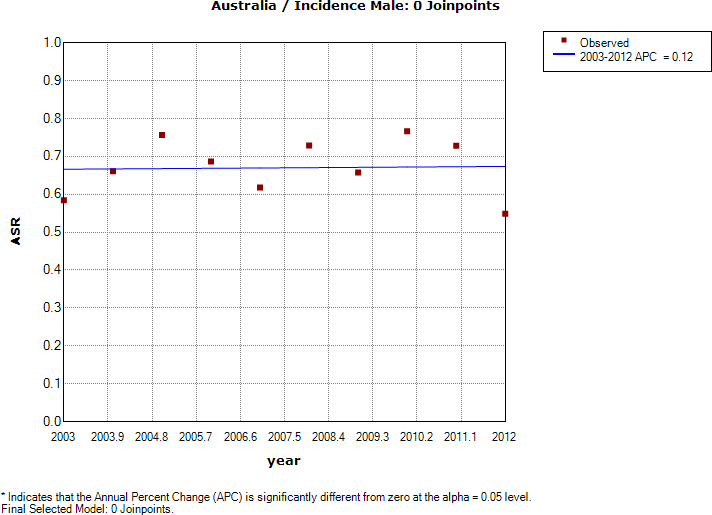 | 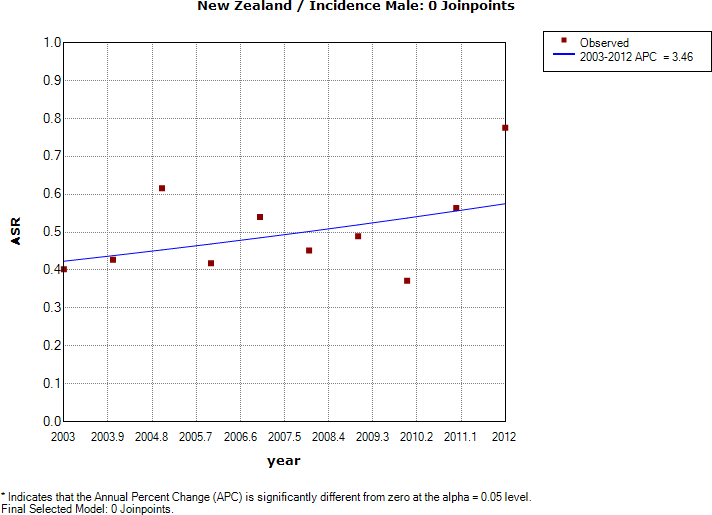 |

| **Northern America** | |
| --- | --- |
| 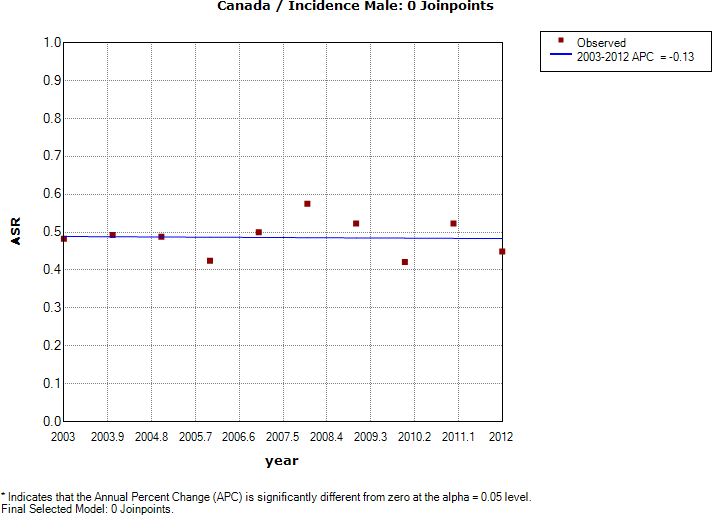 | 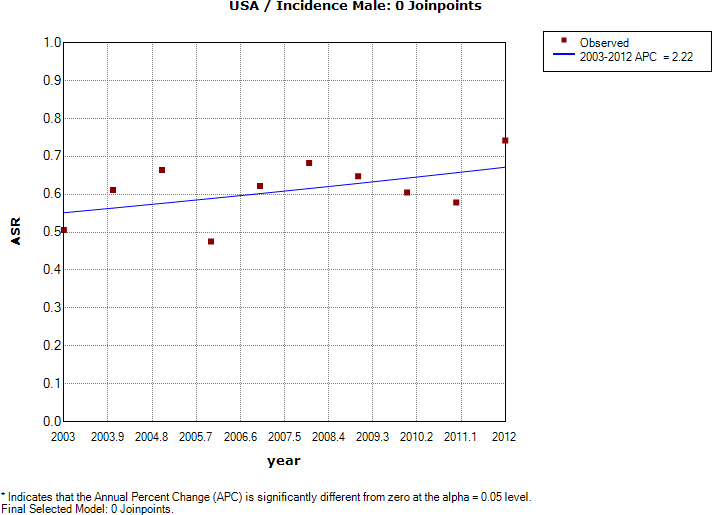 |
| **Southern America** | |
| 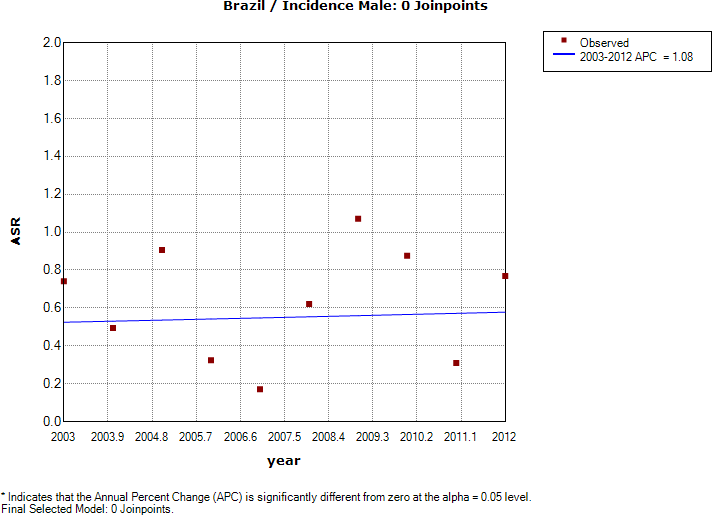 | 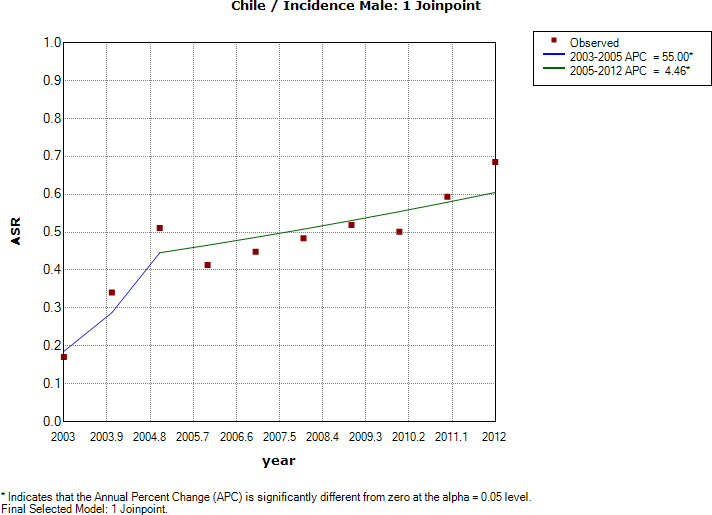 |
| 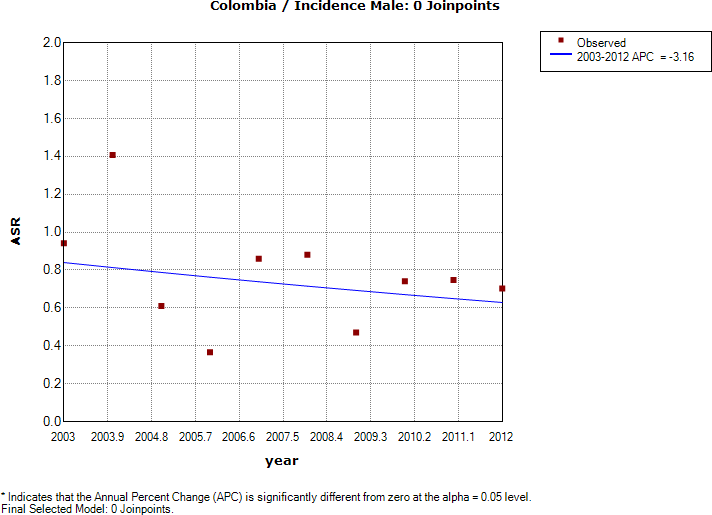 | 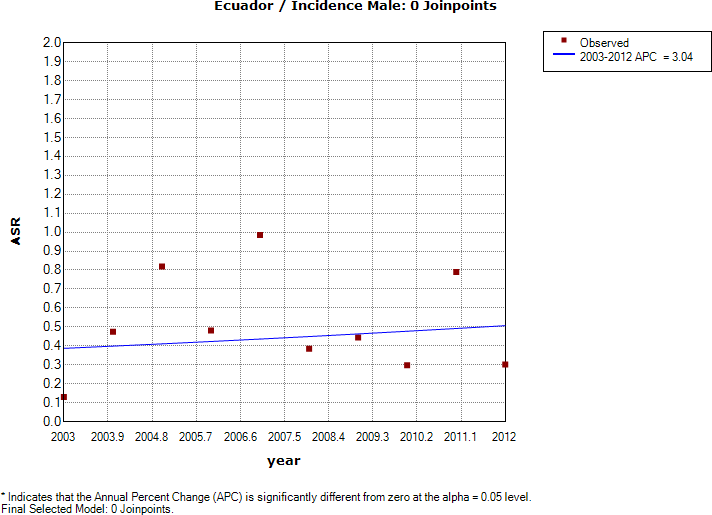 |
| 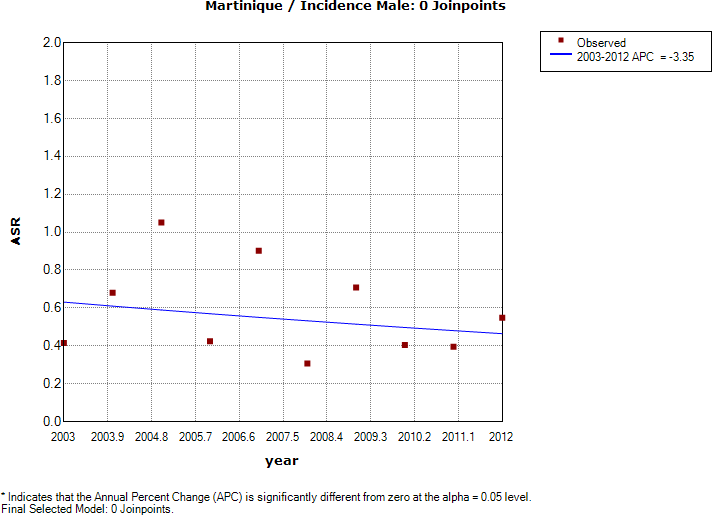 |  |

| **Northern Europe** 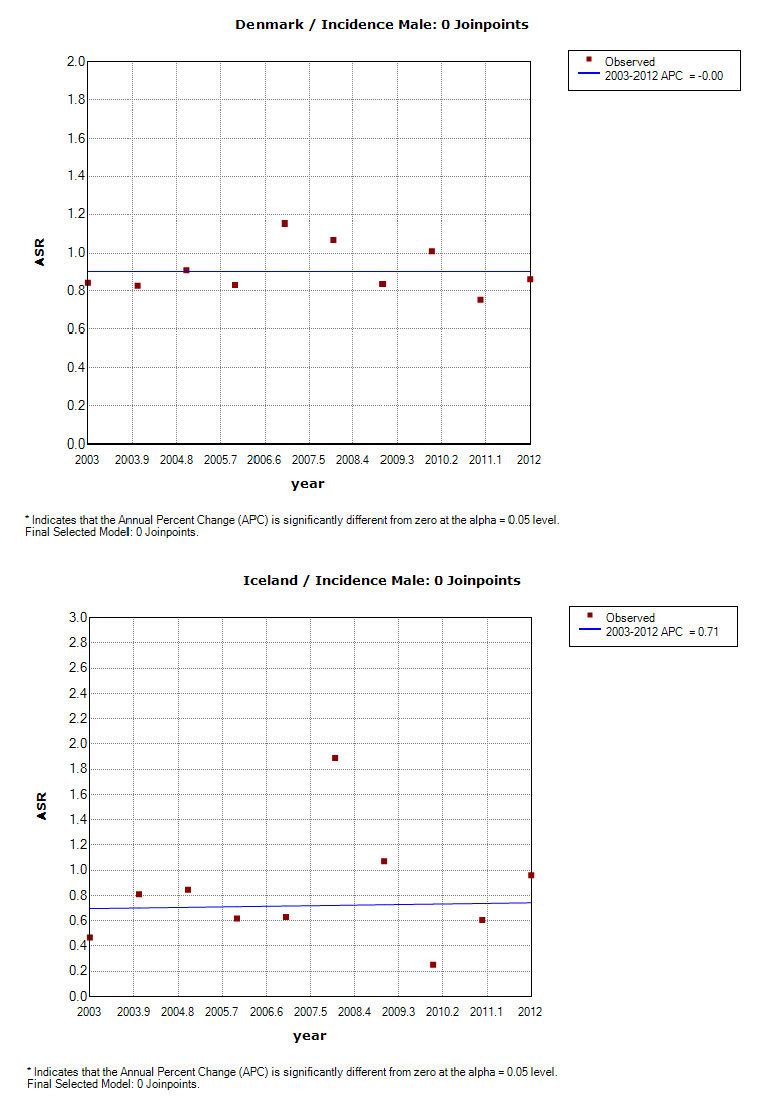 | |
| --- | --- |
|  | 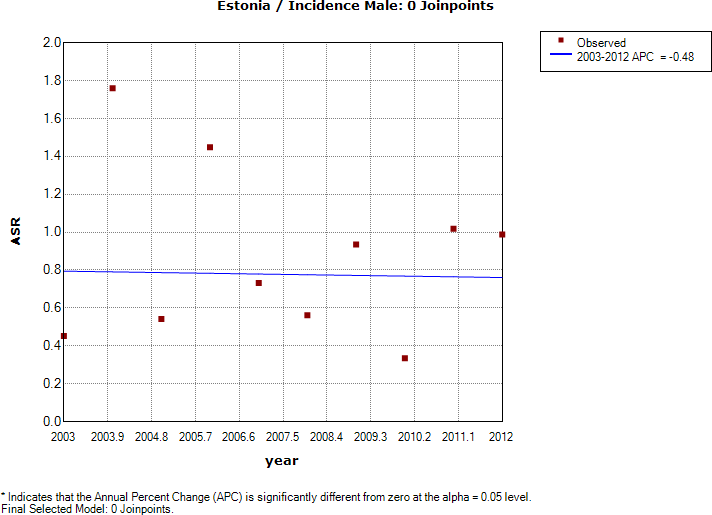 |
|  | 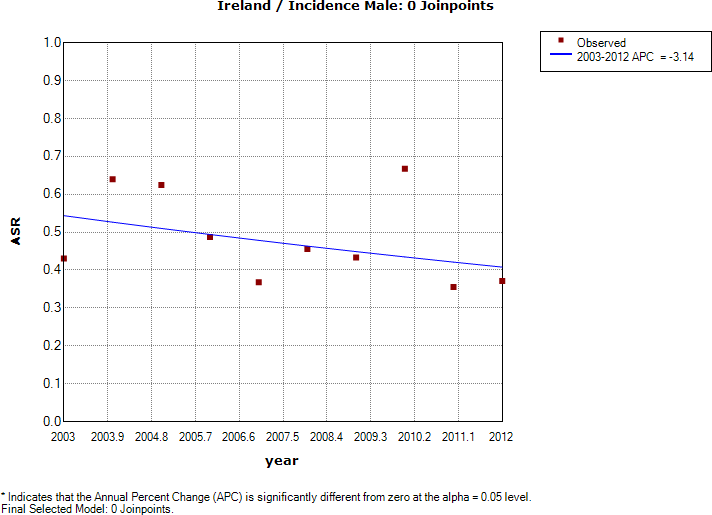 |
| 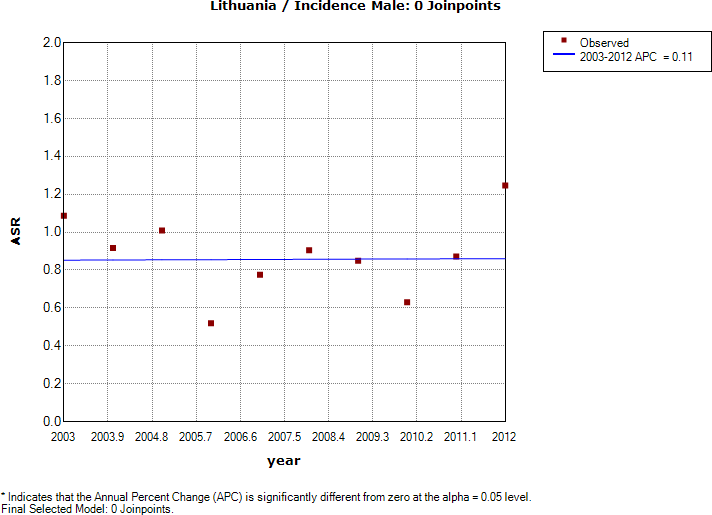 | 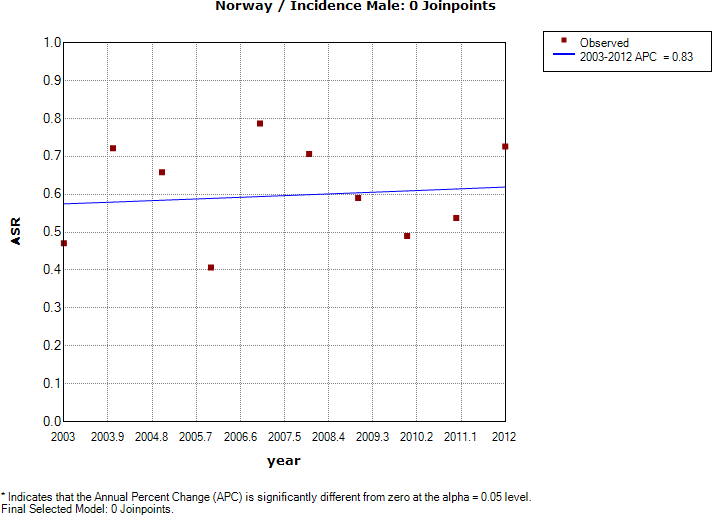 |
| 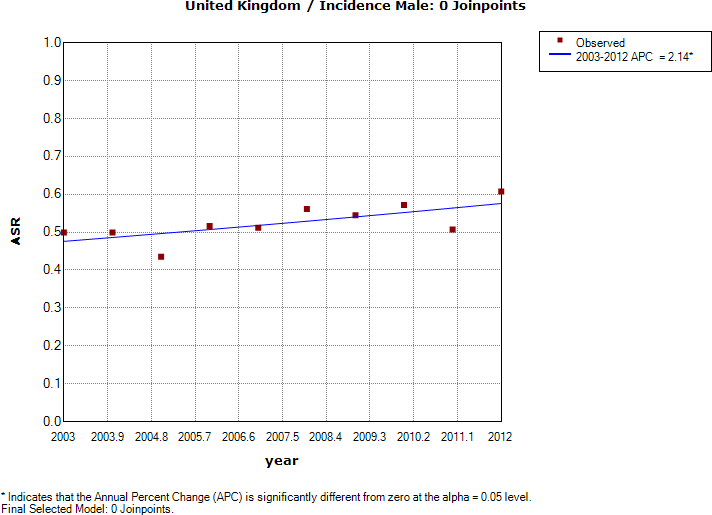 |  |

| **Western Europe** 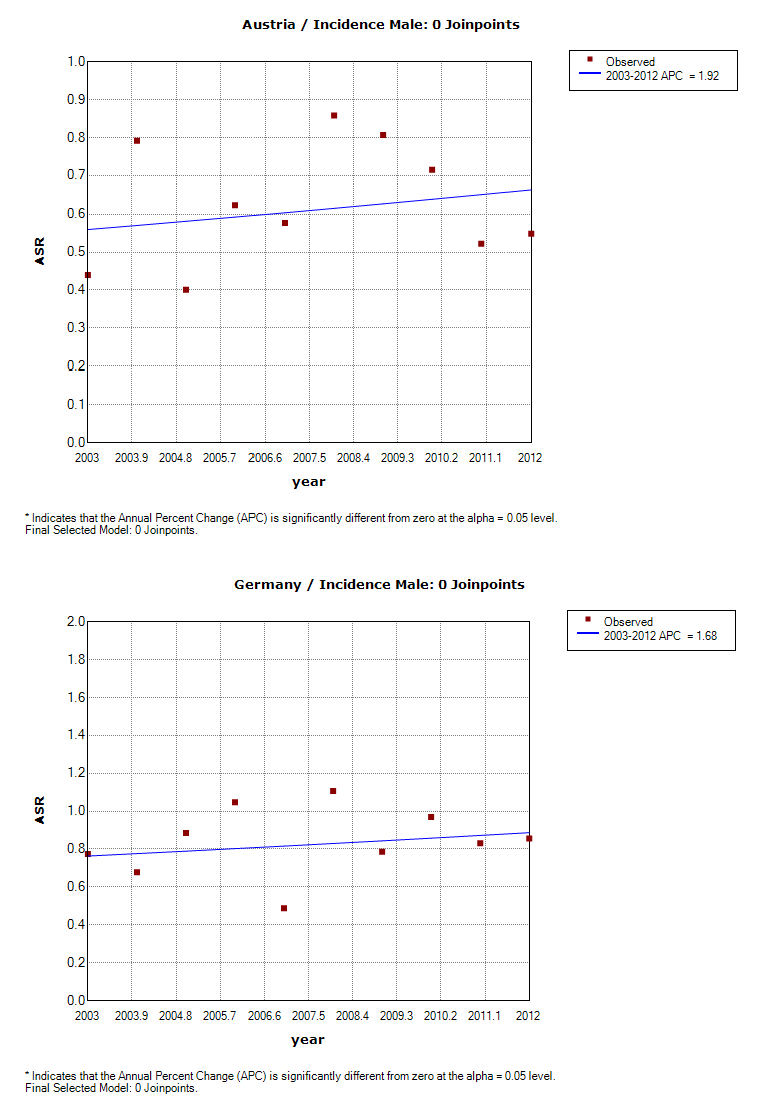 | |
| --- | --- |
|  | 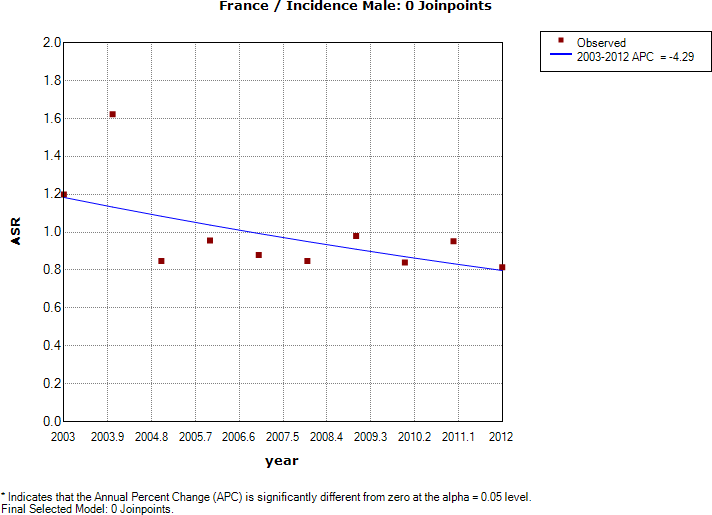 |
|  | 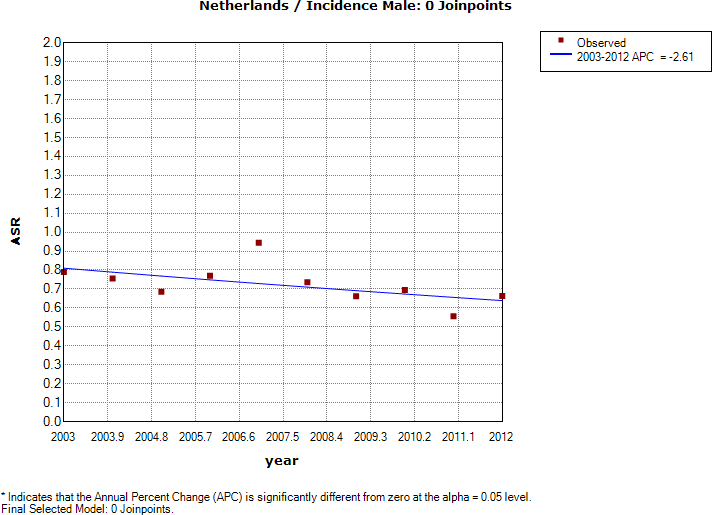 |
| 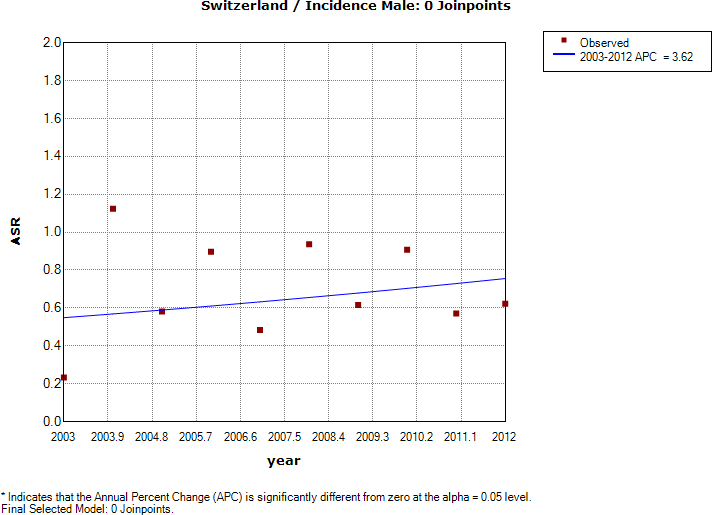 |  |

| **Southern Europe** | |
| --- | --- |
| 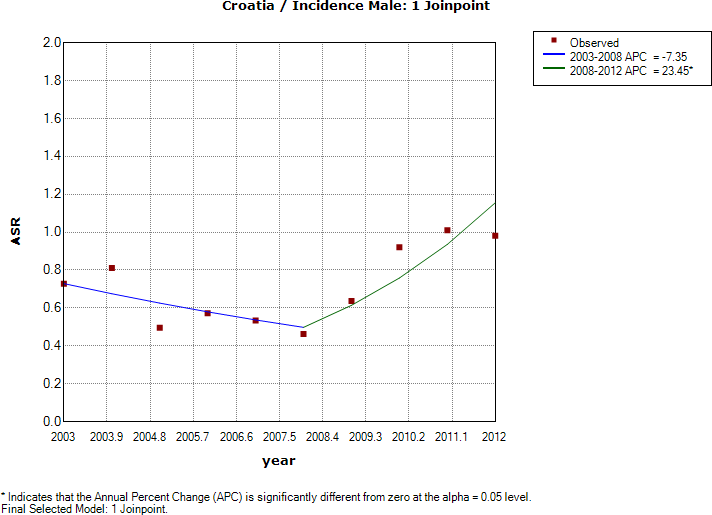 | 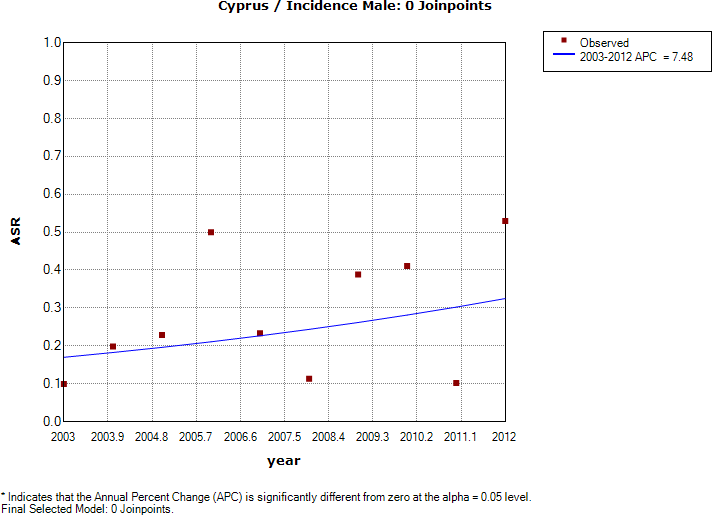 |
| 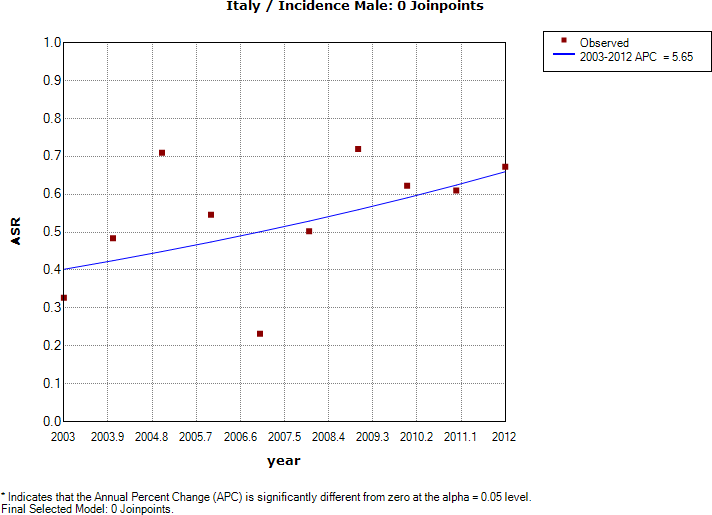 | 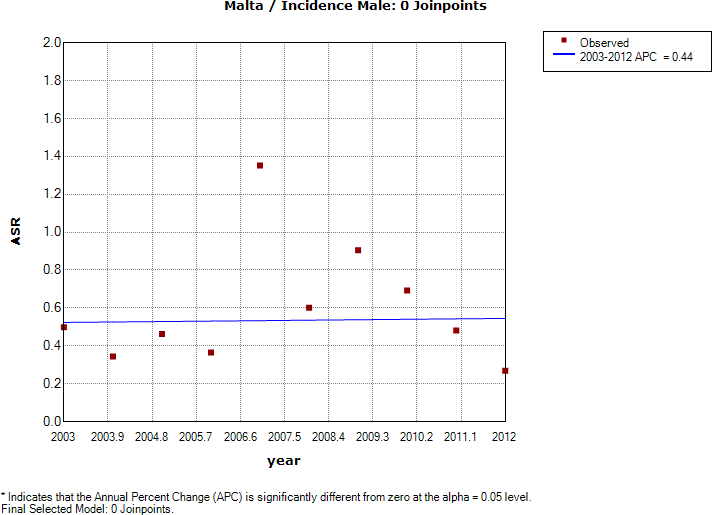 |
| 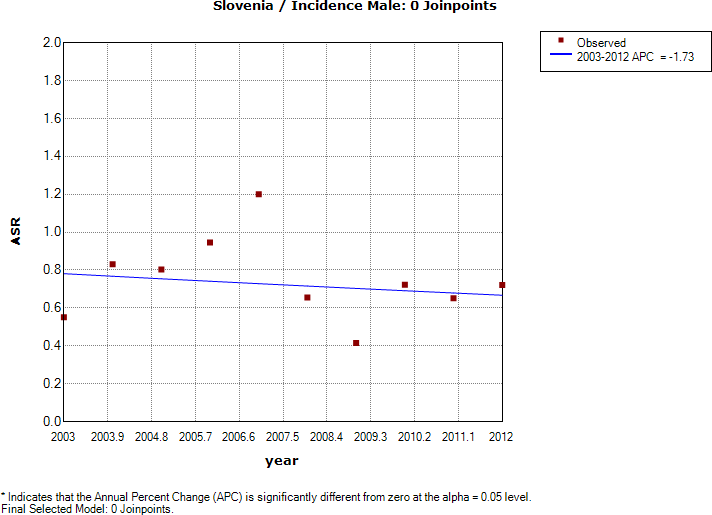 | 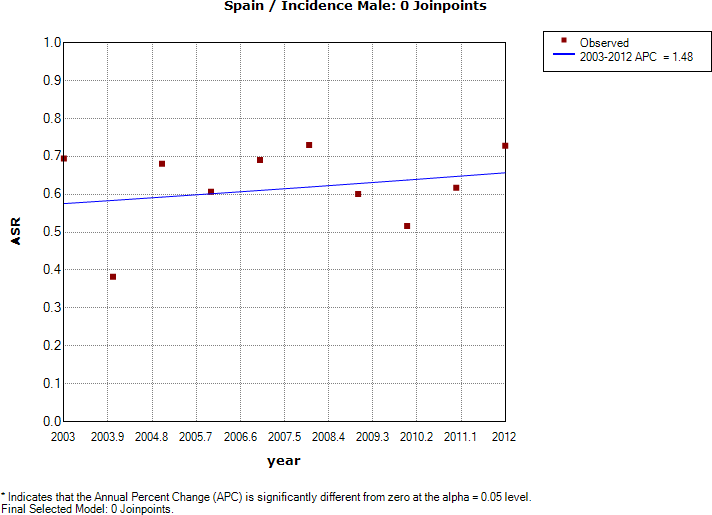 |

| **Eastern Europe** | |
| --- | --- |
| 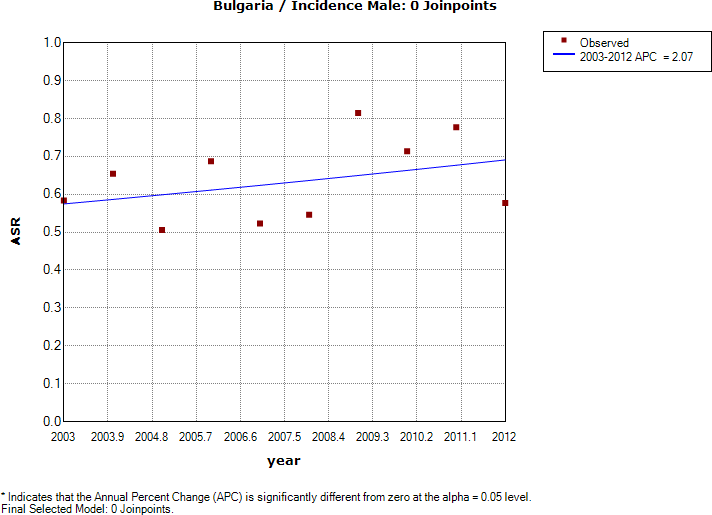 | 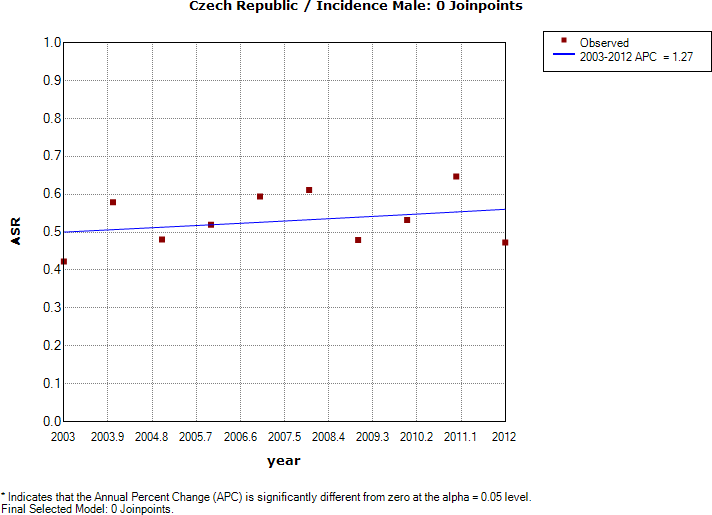 |
| 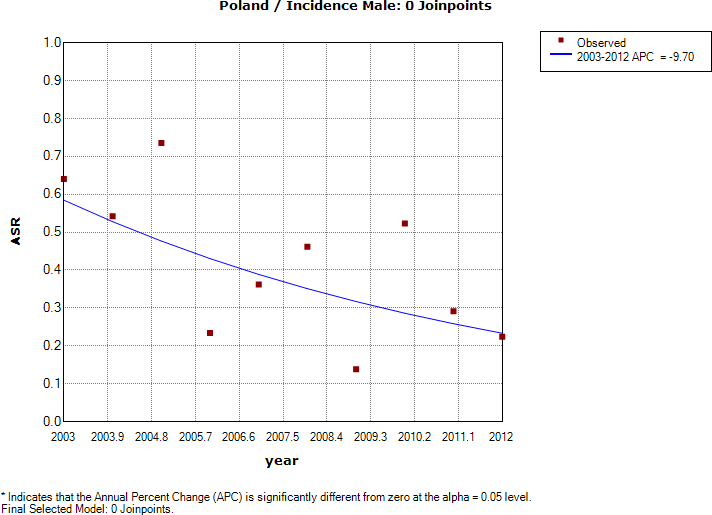 |  |
| **Africa** | |
| 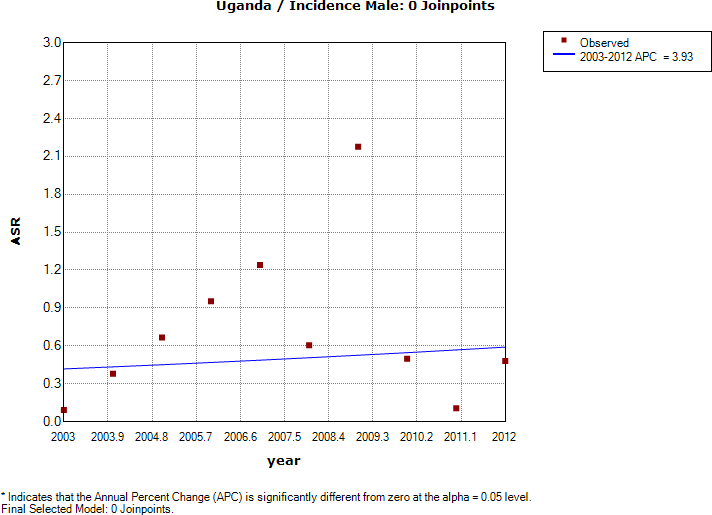 |  |

1. Female

| **Asia** 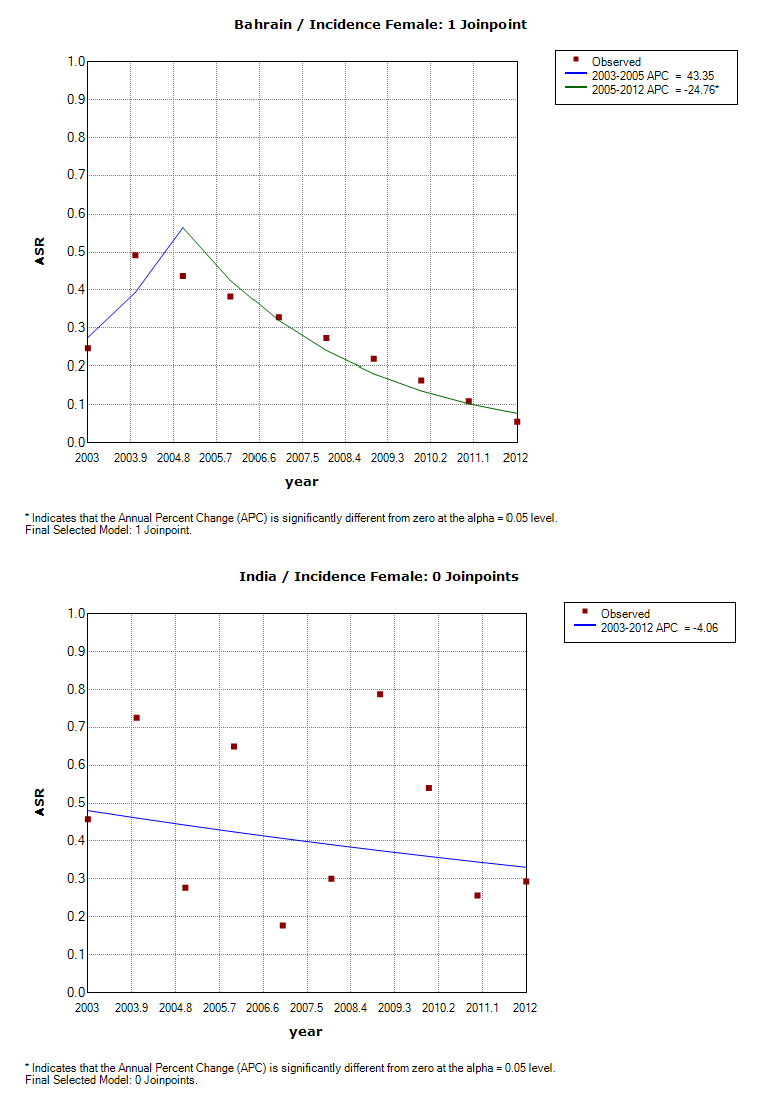 | |
| --- | --- |
|  | 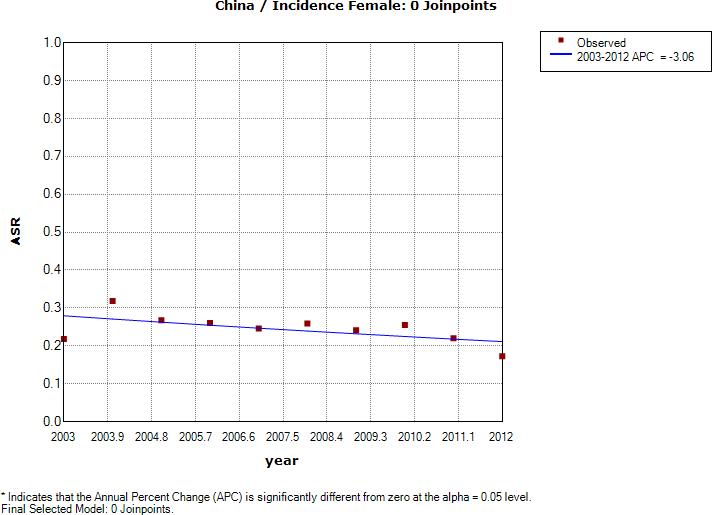 |
|  | 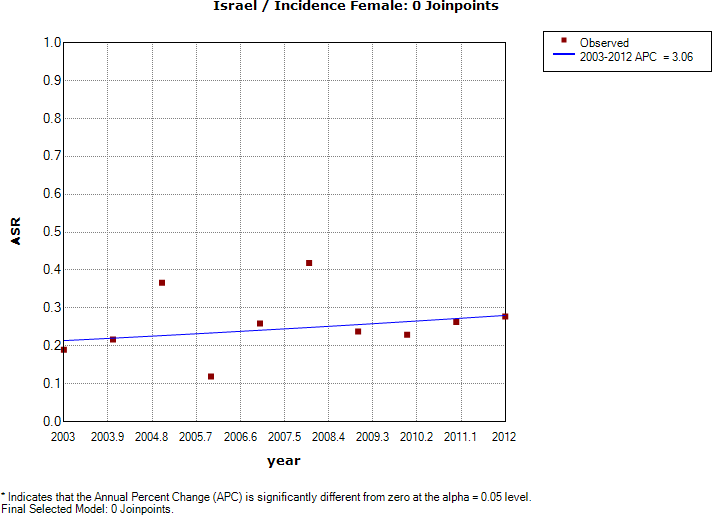 |
| 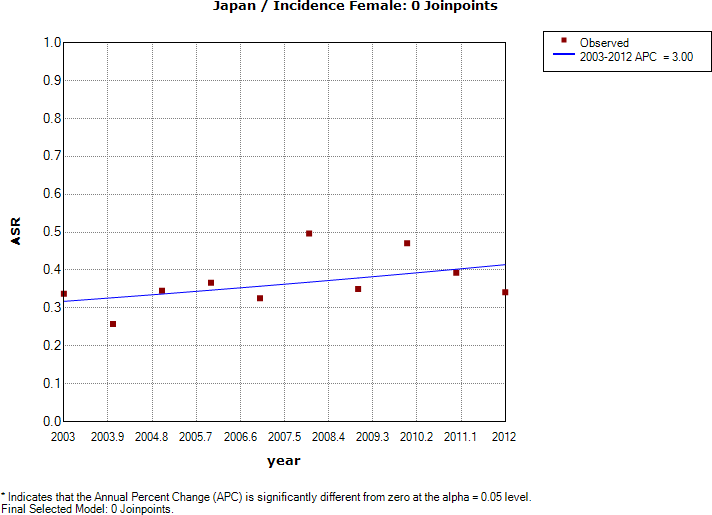 | 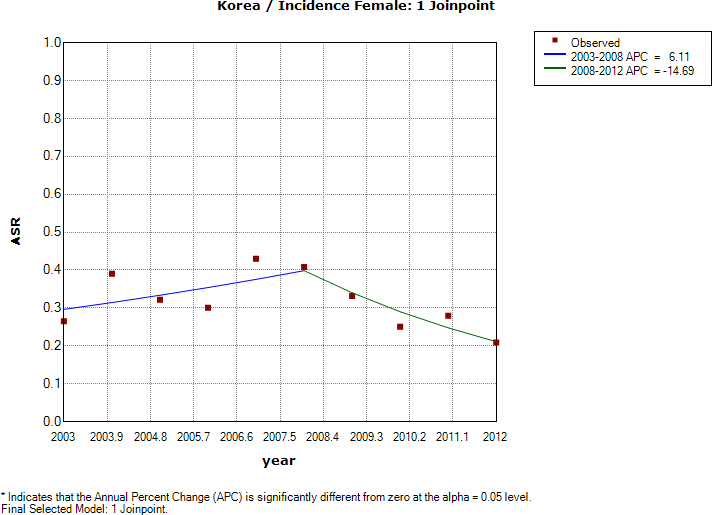 |
| 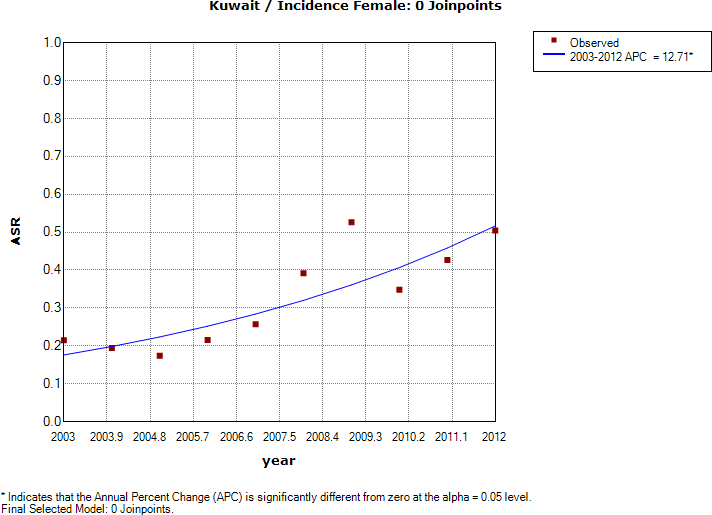 | 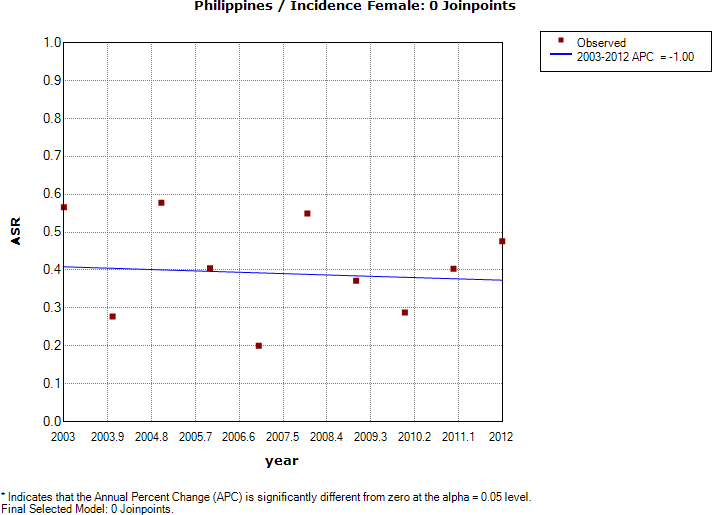 |

| 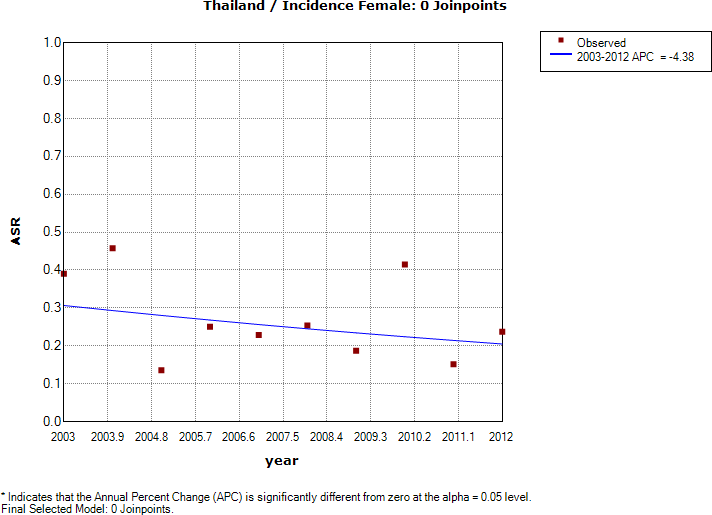 | 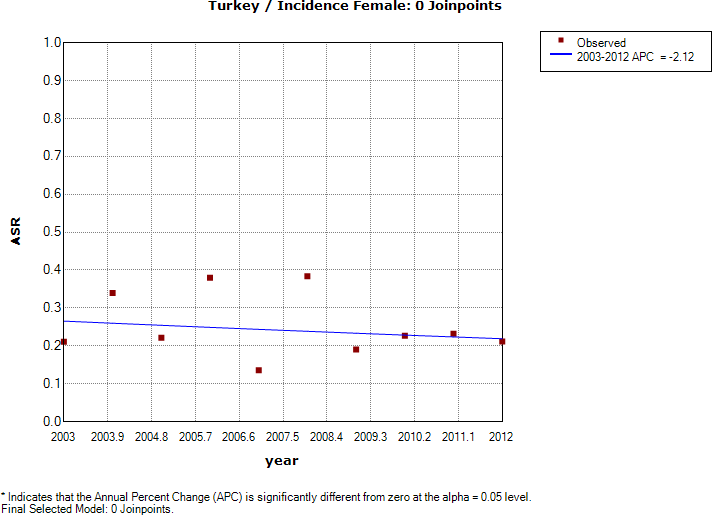 |
| --- | --- |
| **Oceania** | |
| 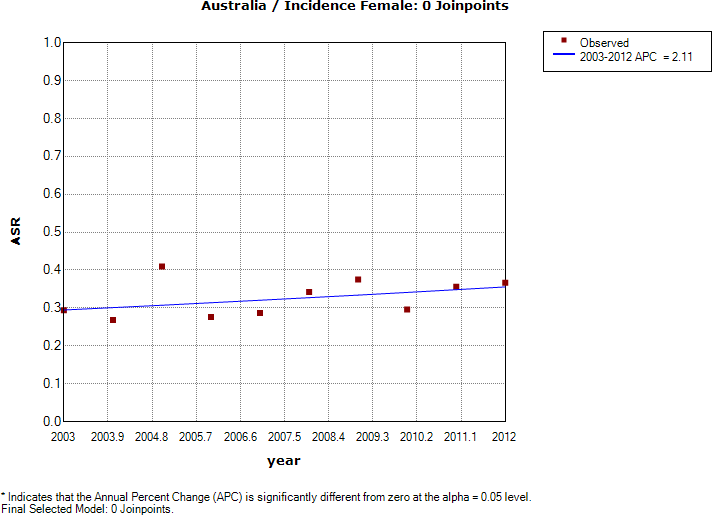 | 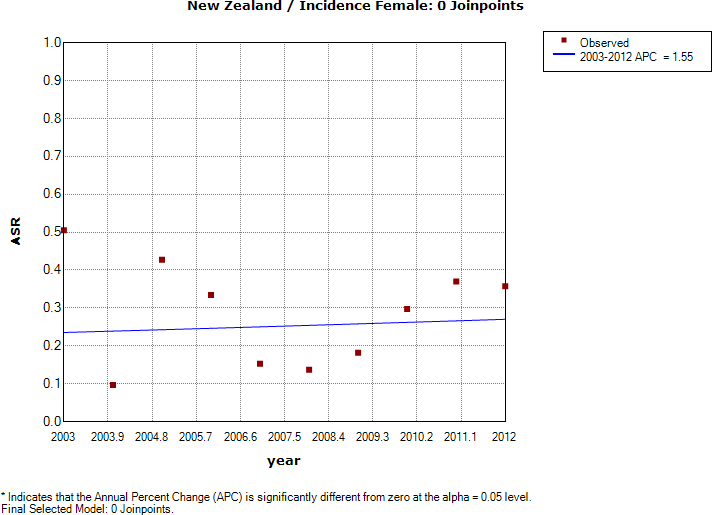 |
| **Northern America** | |
| 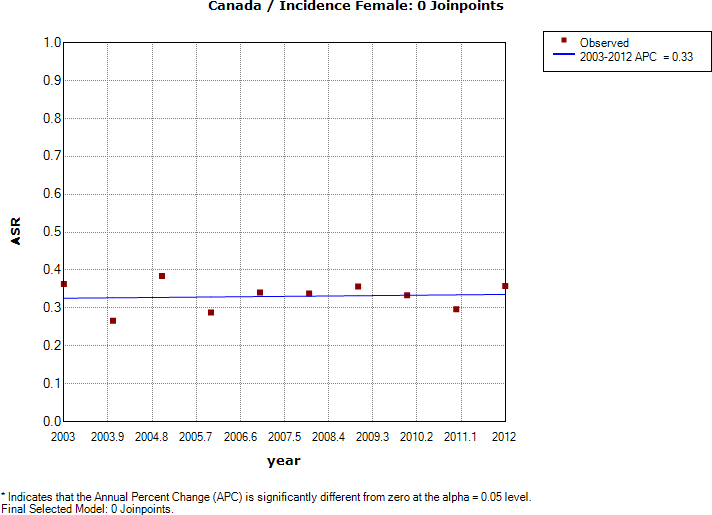 | 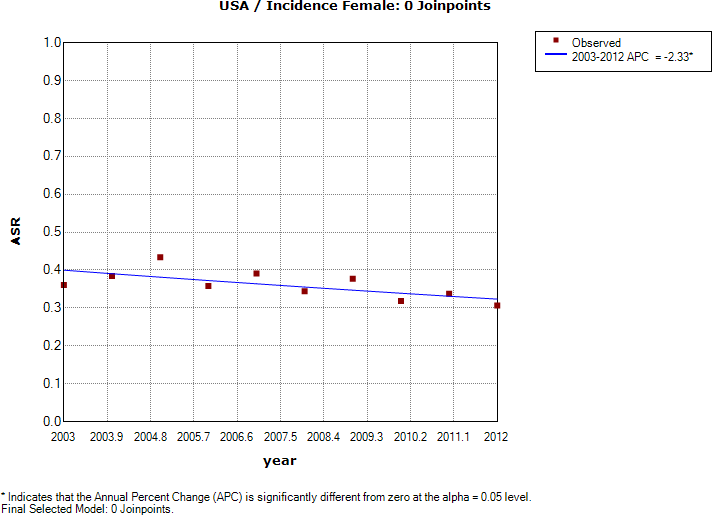 |

| **Southern America** | |
| --- | --- |
| 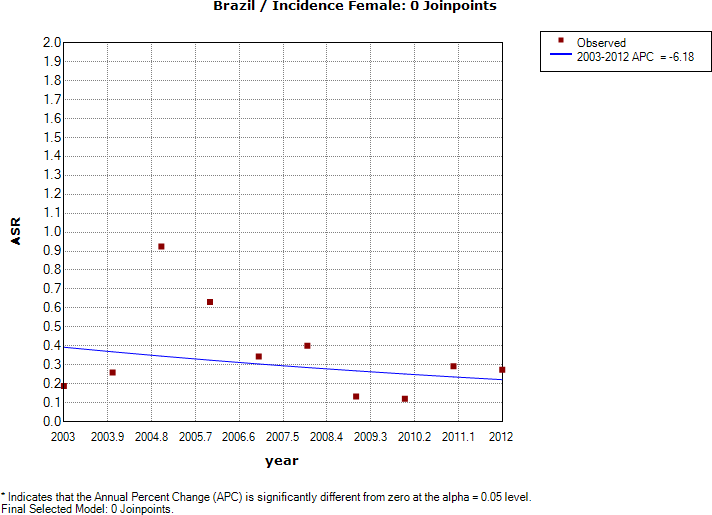 | 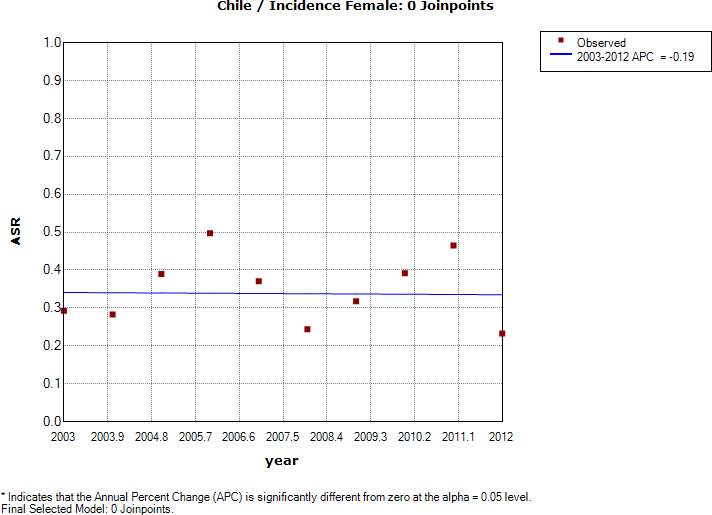 |
| 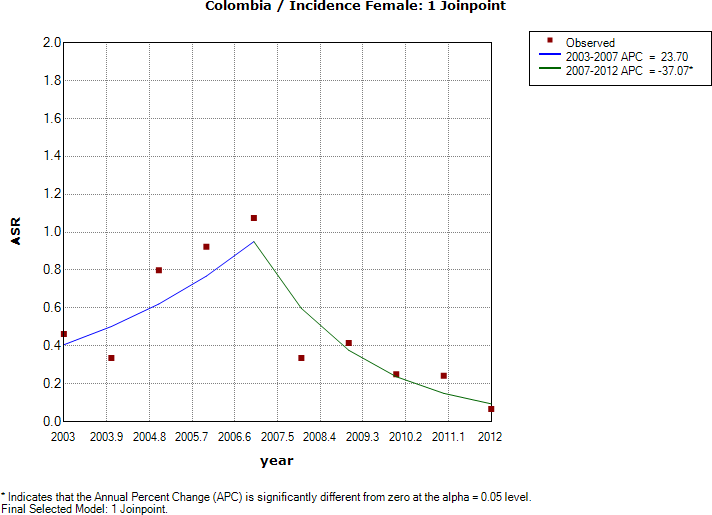 | 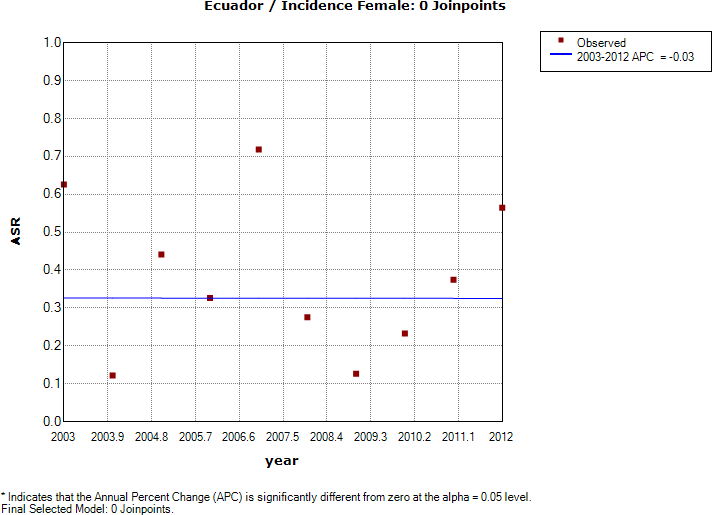 |
| 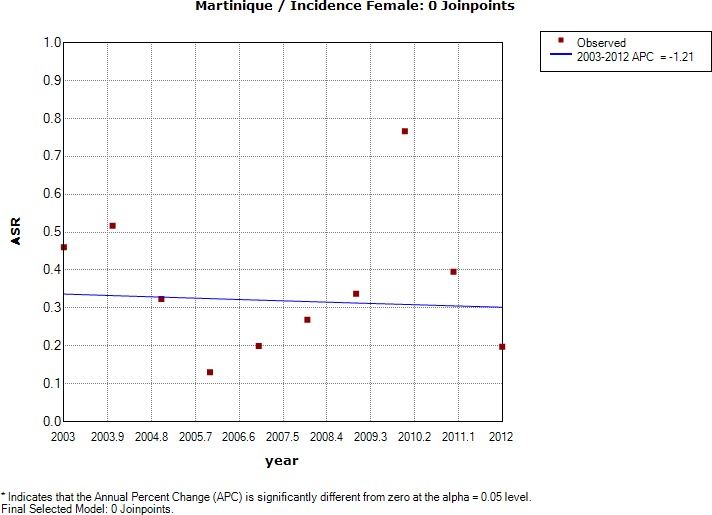 |  |


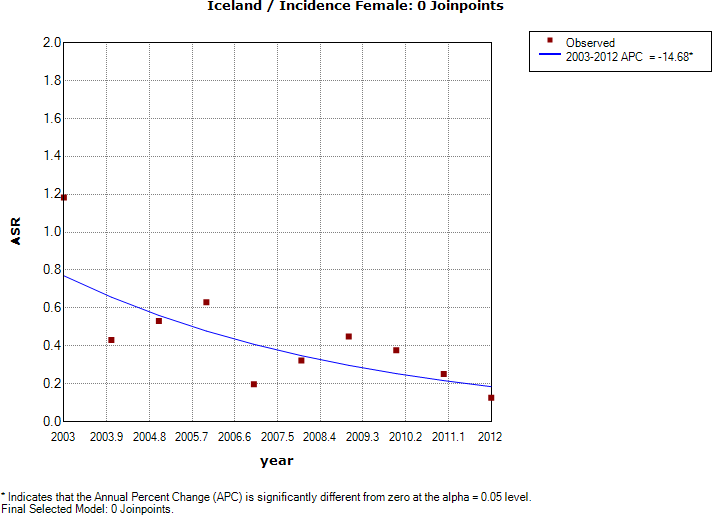

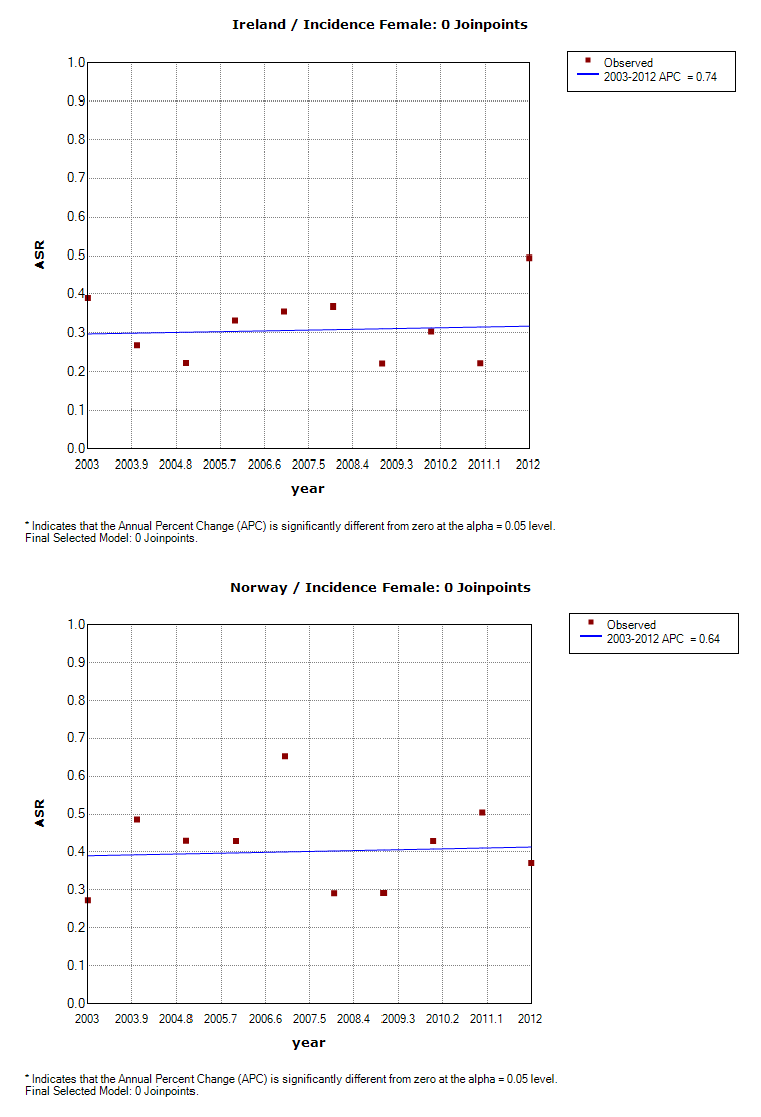

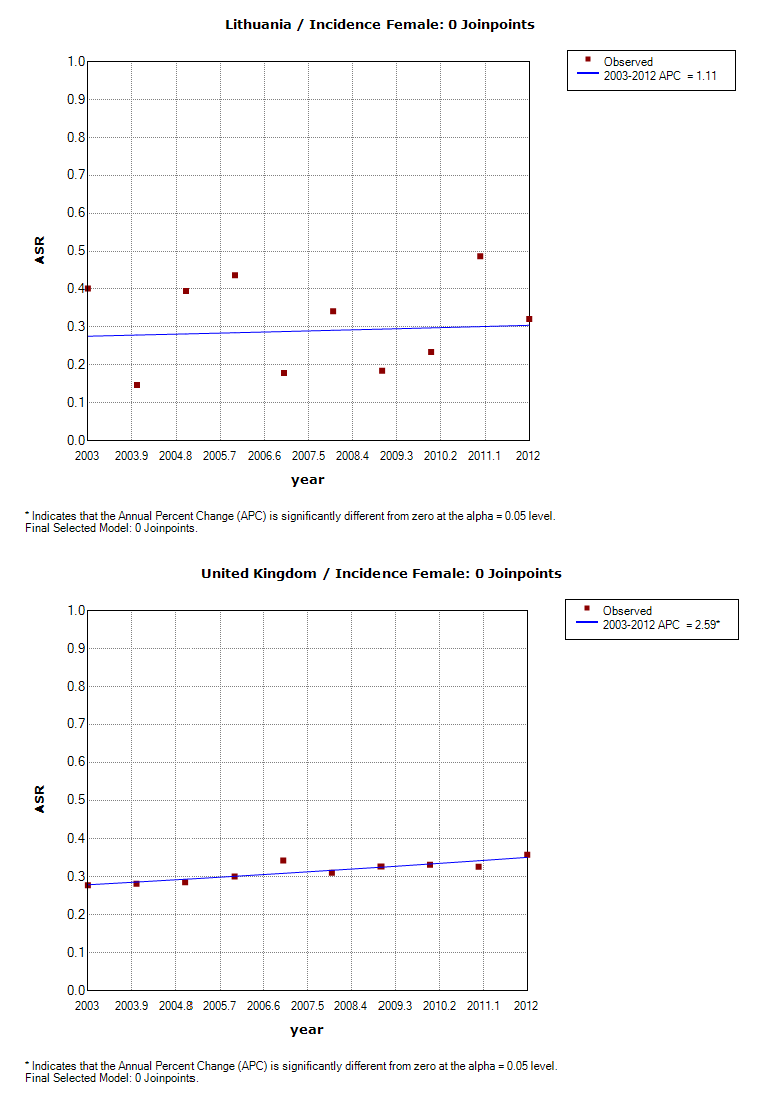
**Northern Europe**


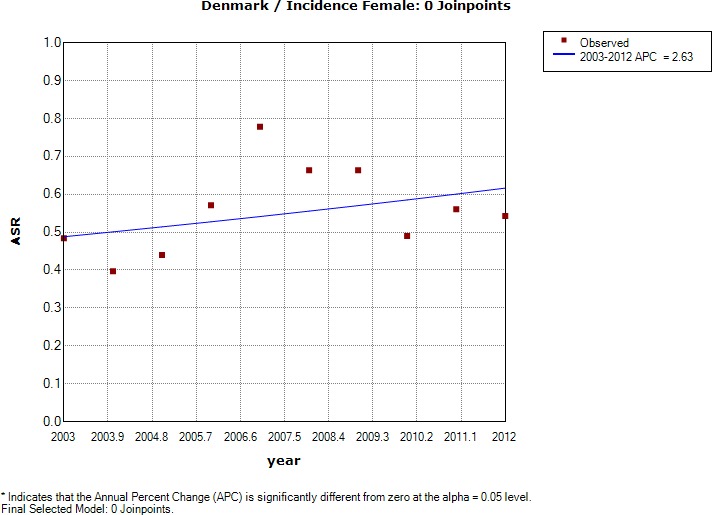

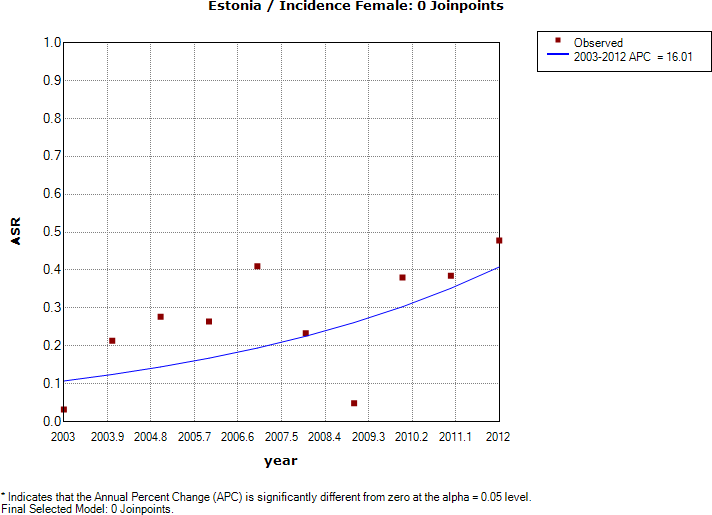


| **Western Europe** 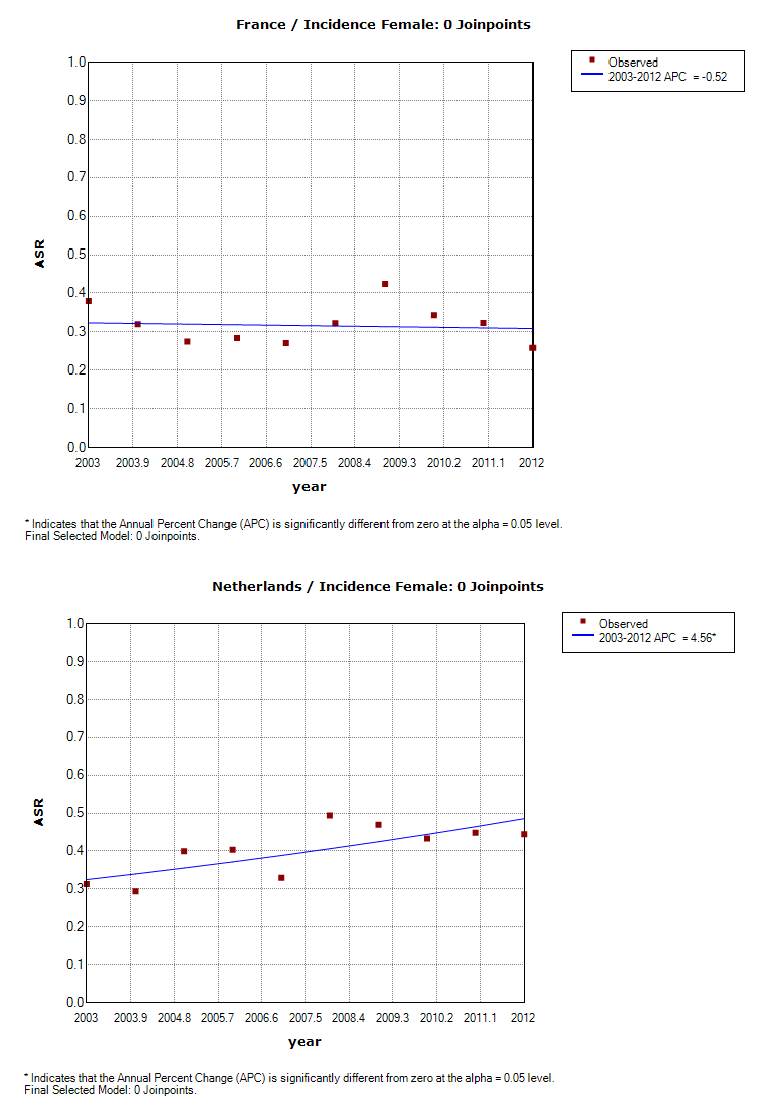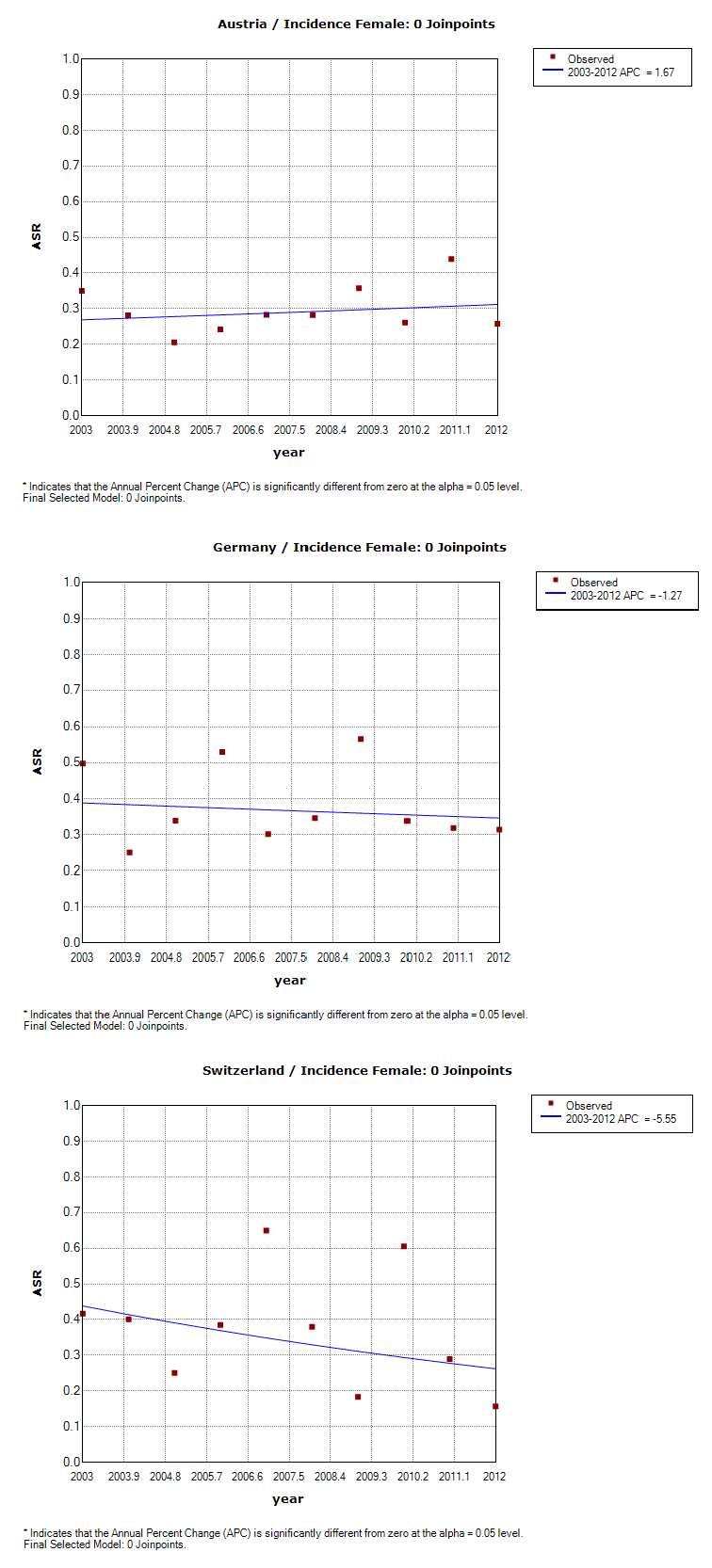 | |
| --- | --- |
|  |  |
|  |  |
|  |  |

| **Southern Europe** 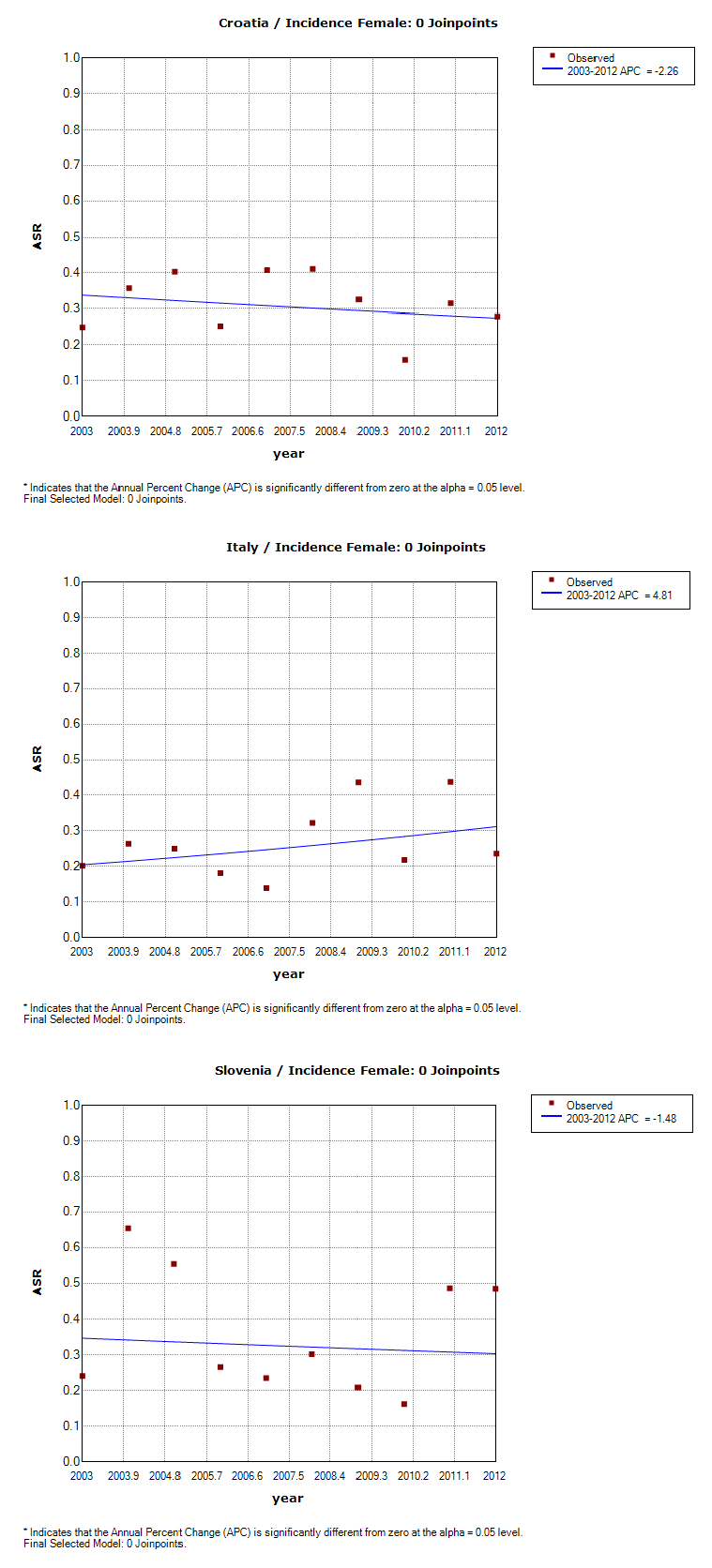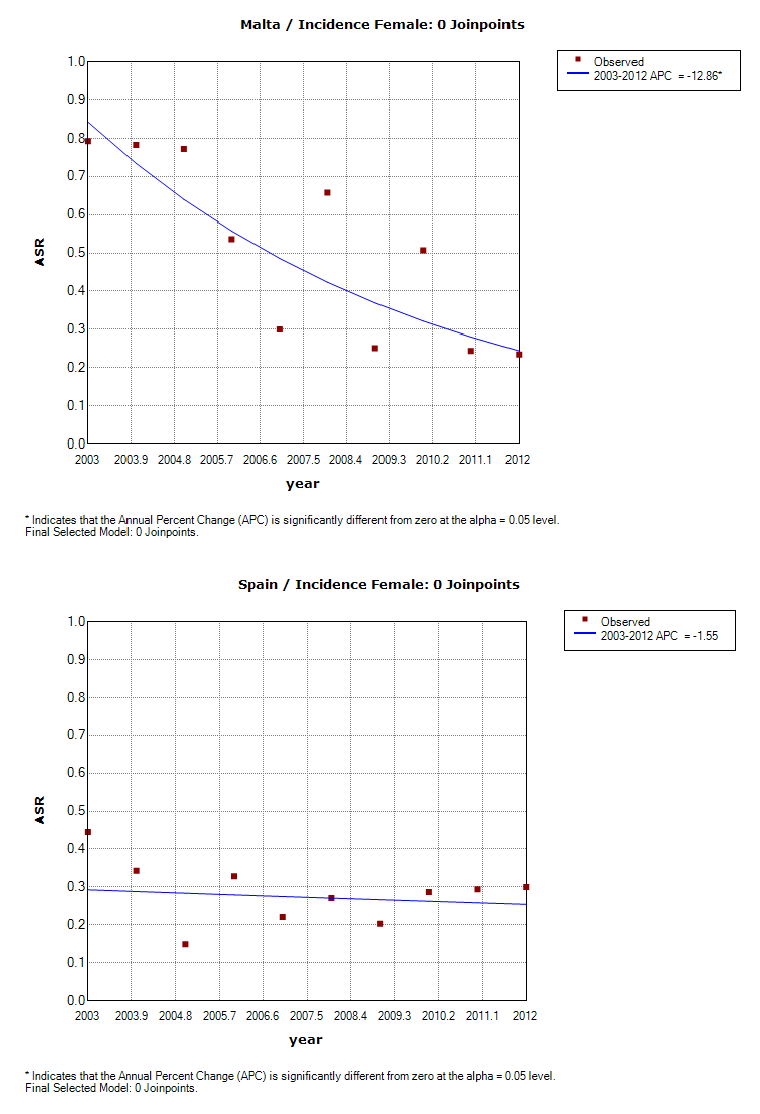 | |
| --- | --- |
|  | 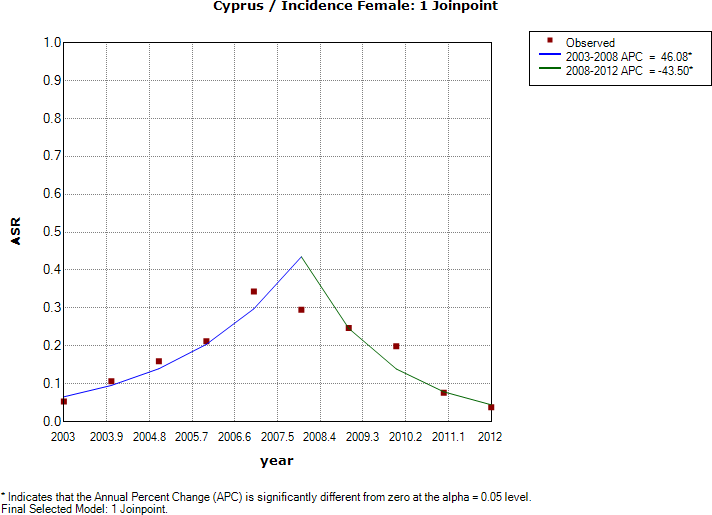 |
|  |  |
|  |  |

| **Eastern Europe** 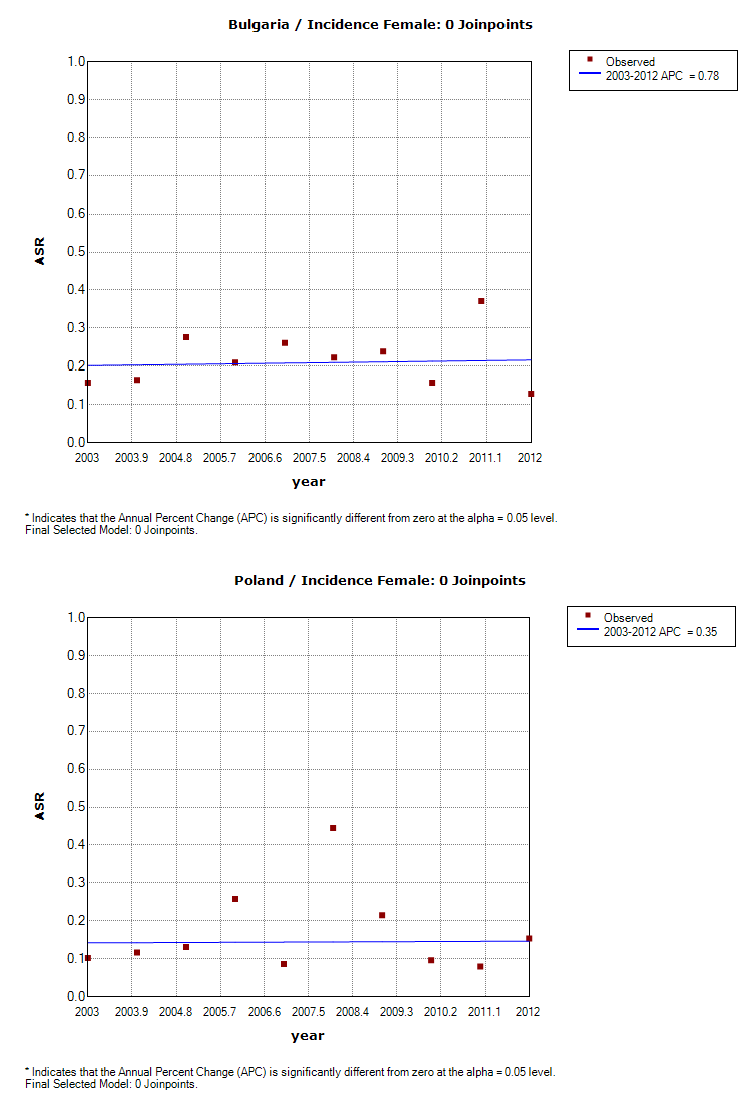 | |
| --- | --- |
|  | 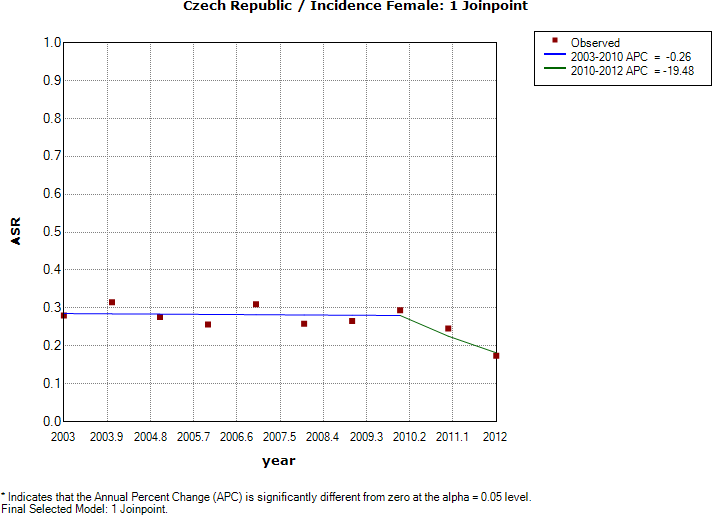 |
|  |  |
| **Africa** | |
| 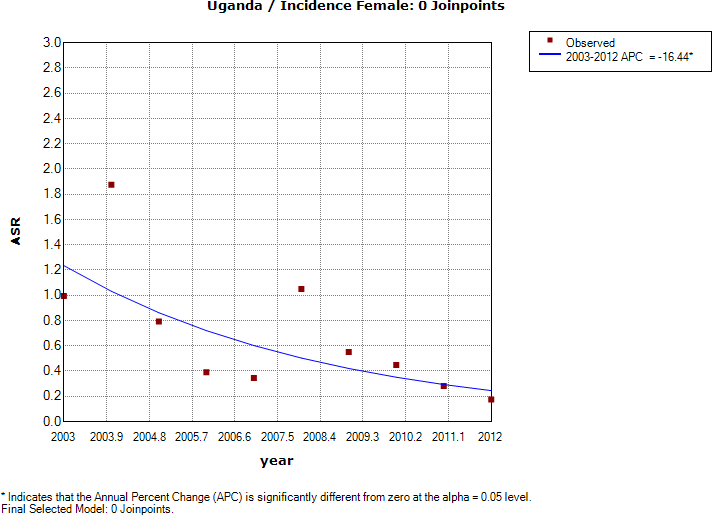 |  |

1. Both

| **Asia** 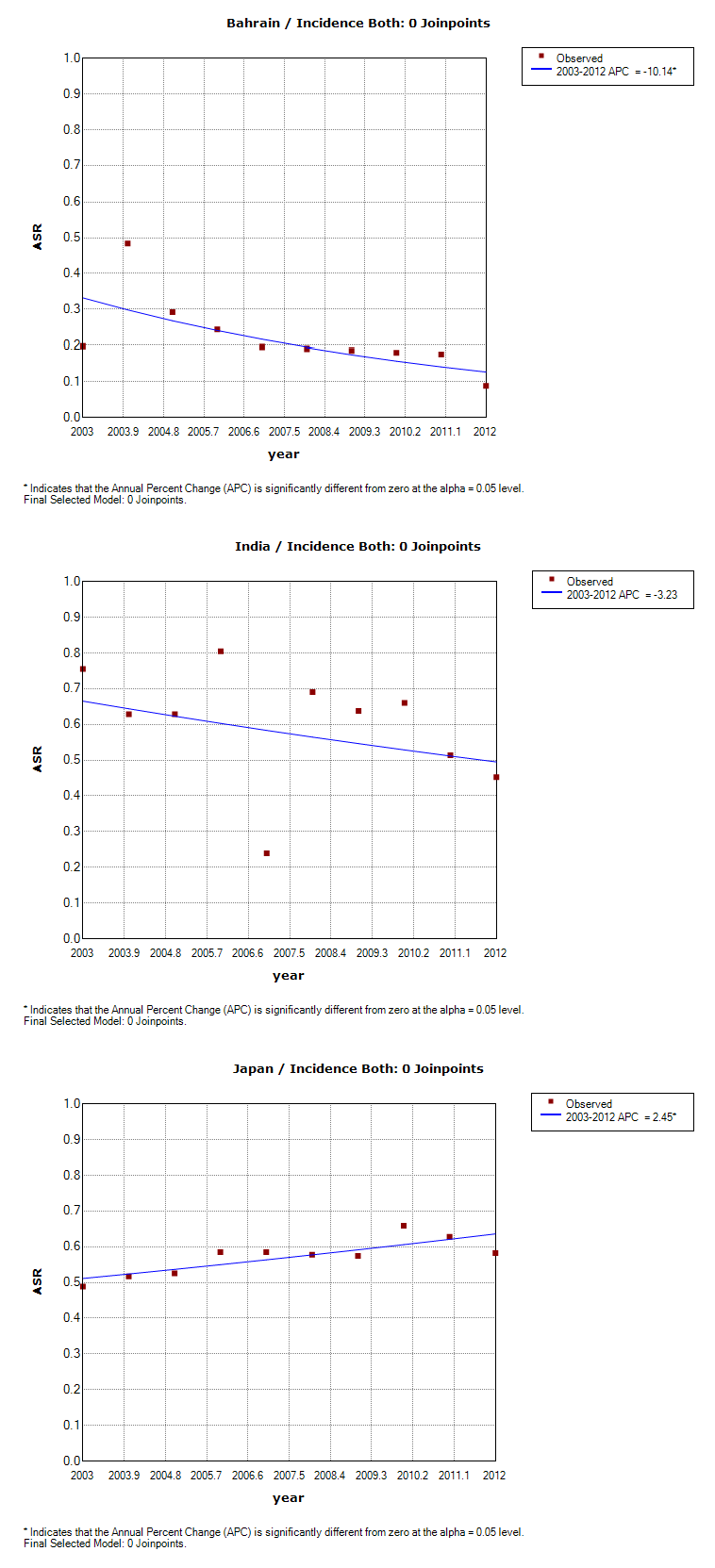 | |
| --- | --- |
|  | 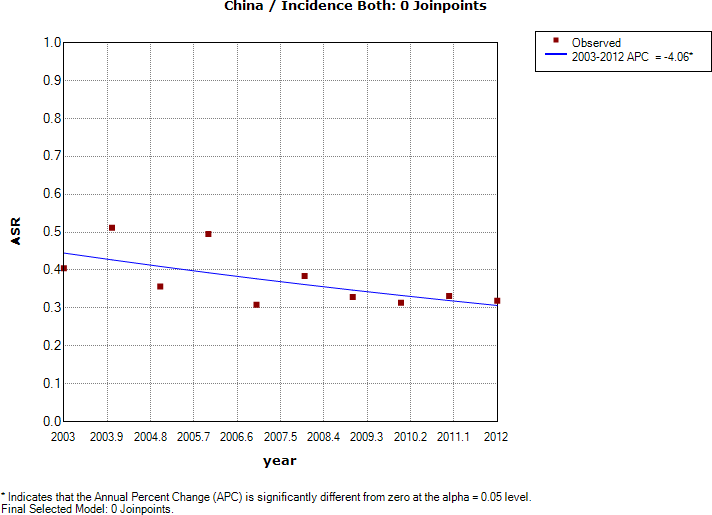 |
|  | 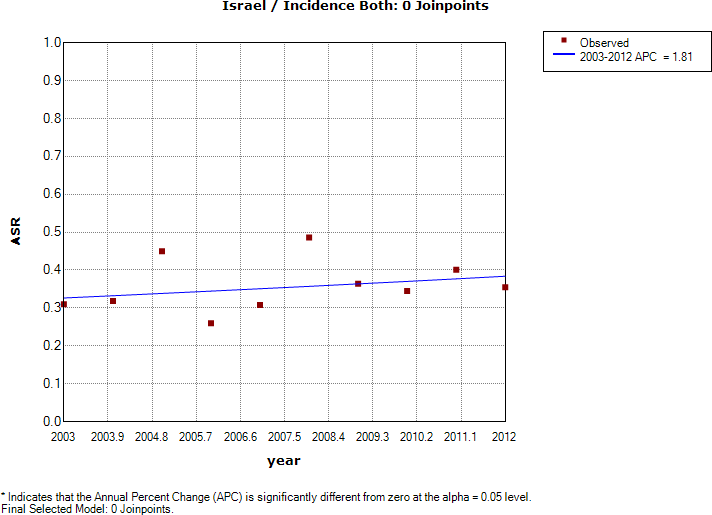 |
|  | 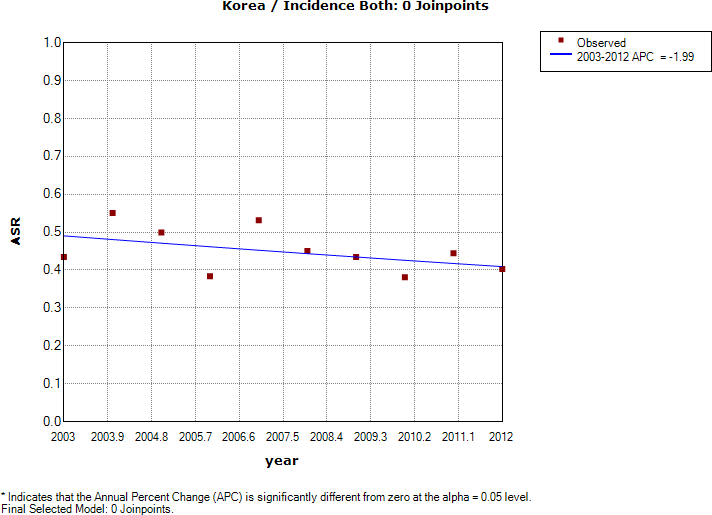 |

|  | 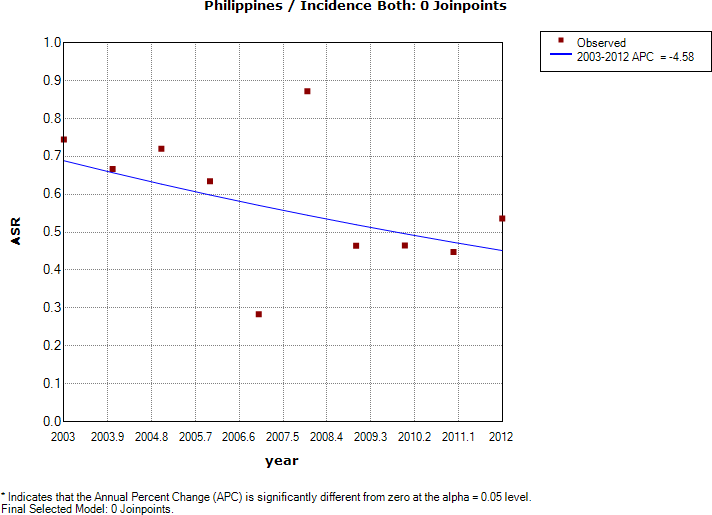 |
| --- | --- |
|  | 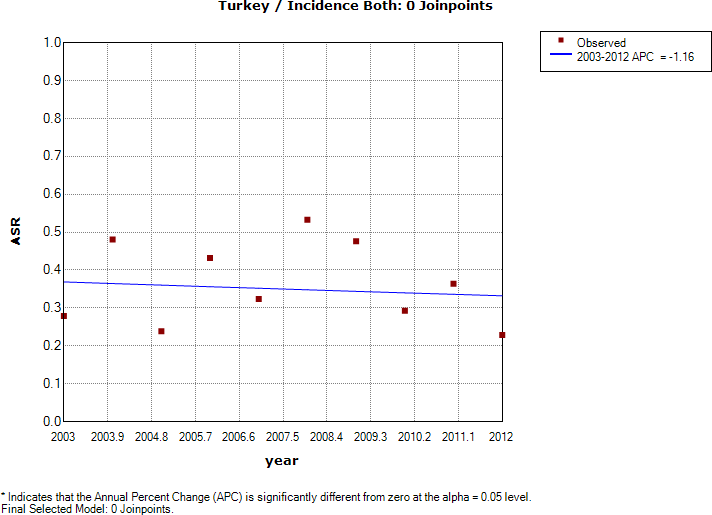 |
| **Oceania** 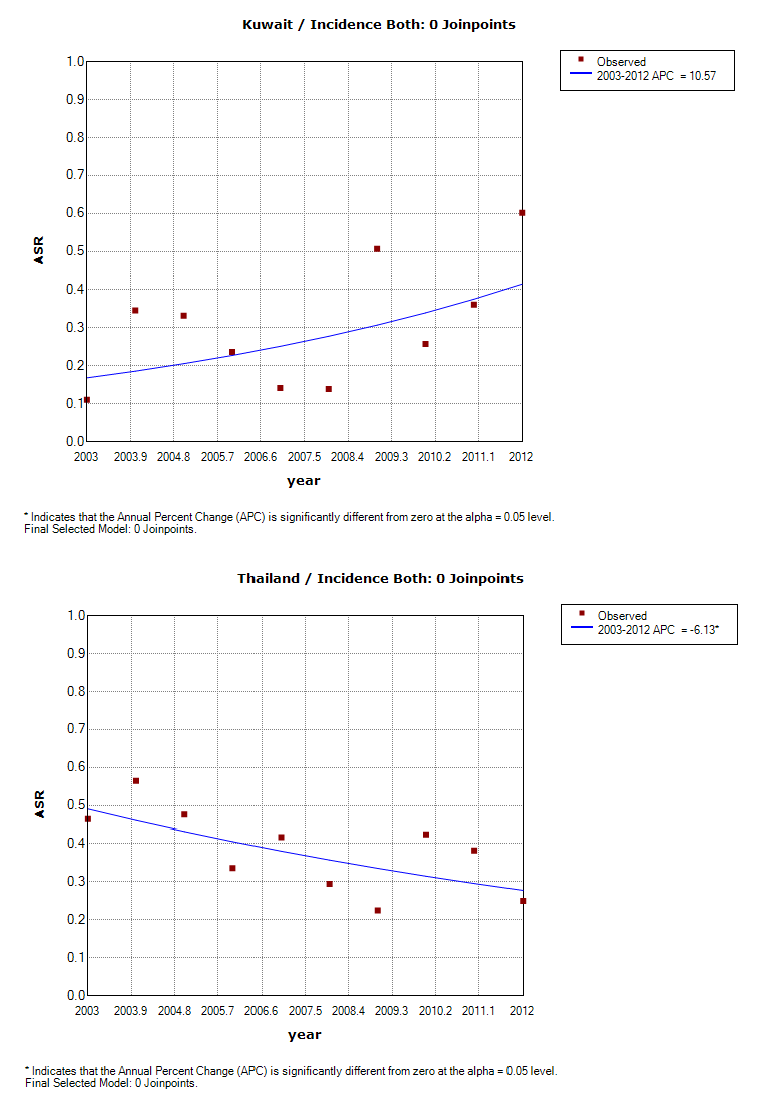 | |
| 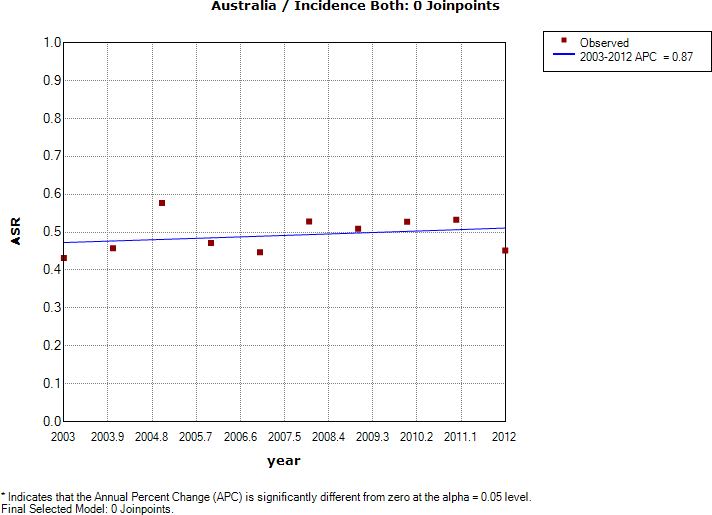 | 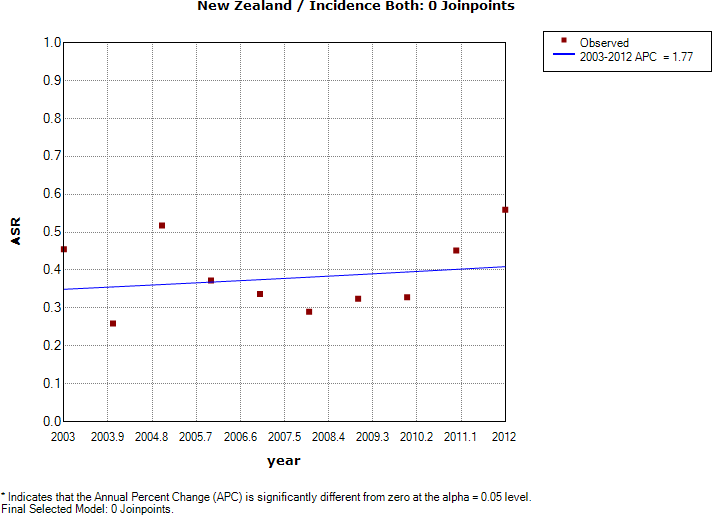 |

| **Northern America** | |
| --- | --- |
| 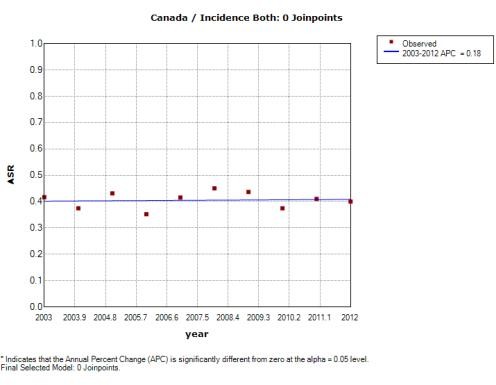 | 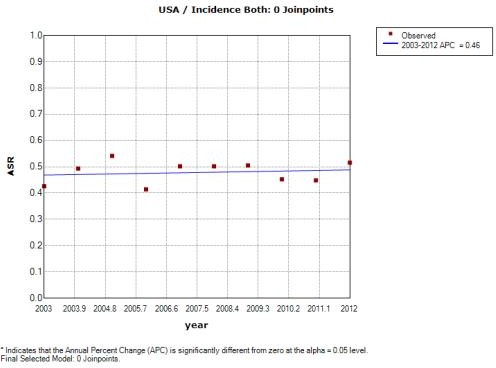 |
| **Southern America** 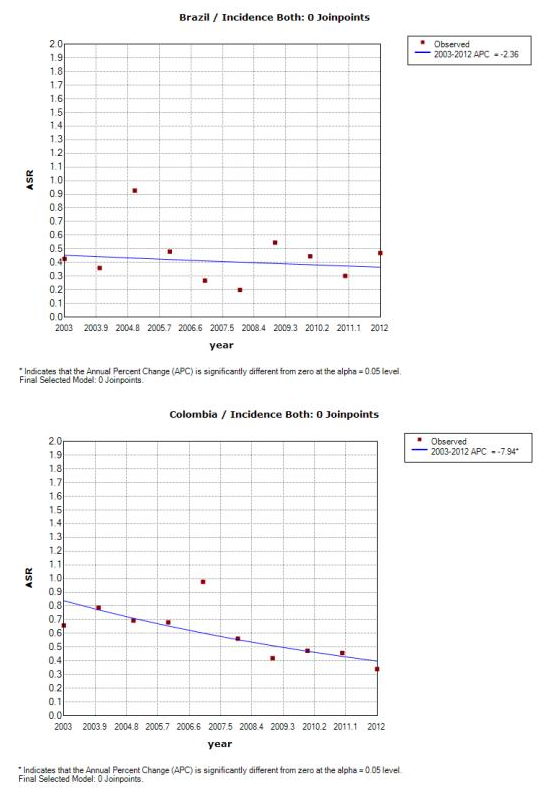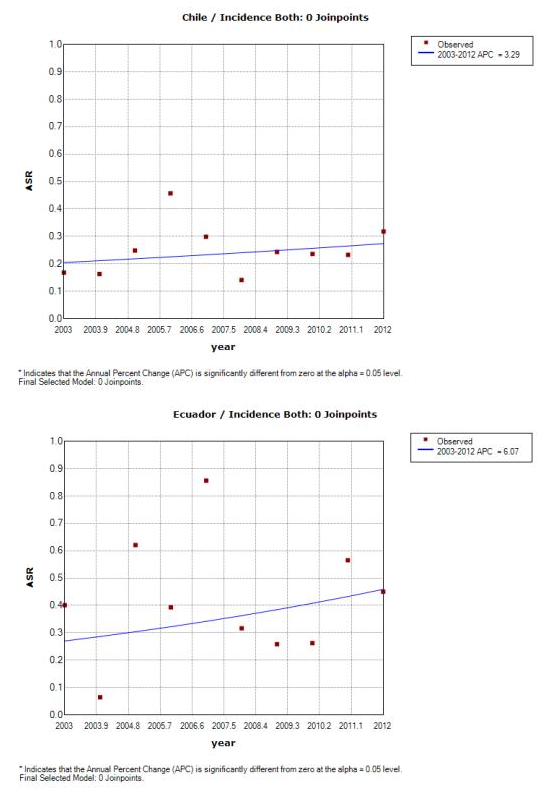 | |
|  |  |
|  |  |
| 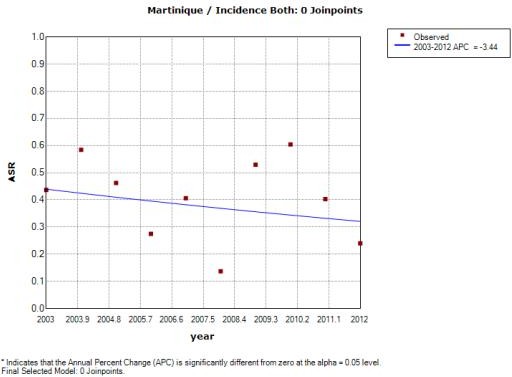 |  |

**Northern Europe**


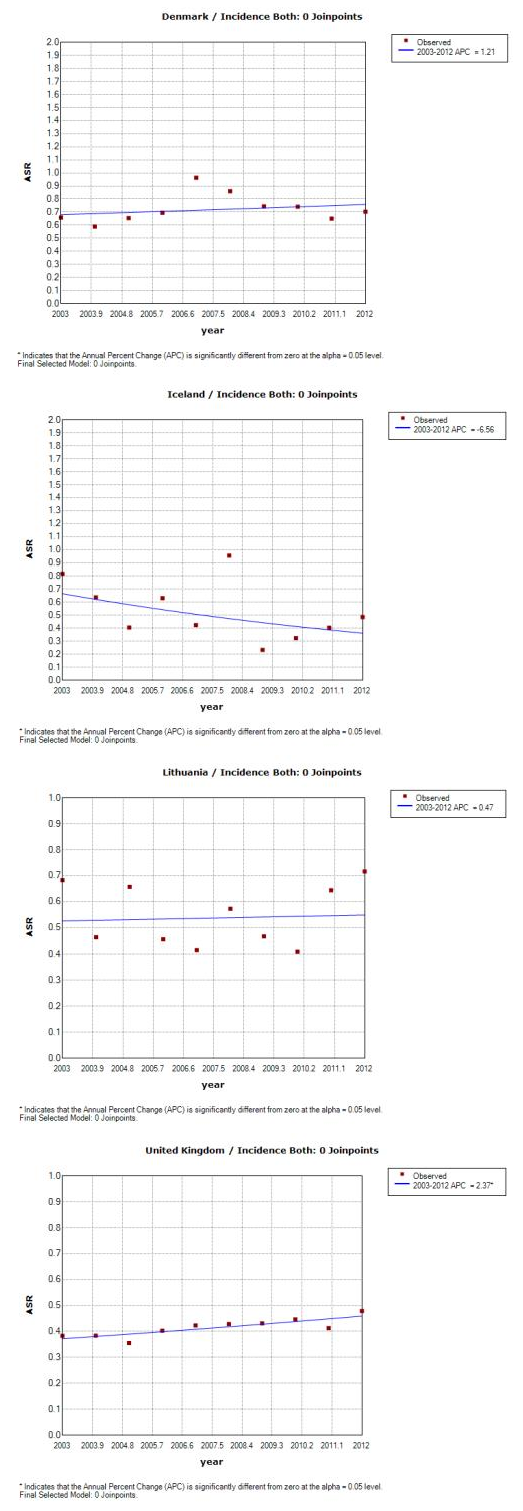


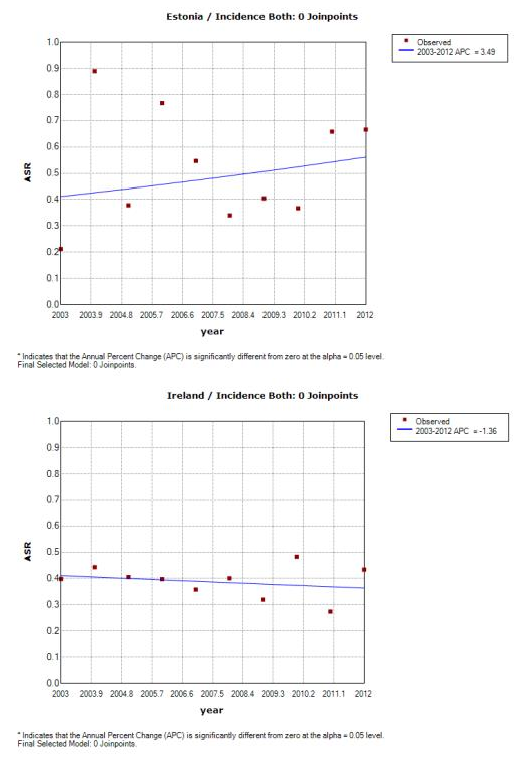

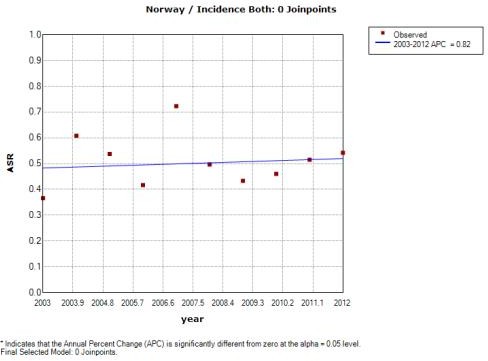


**Western Europe**


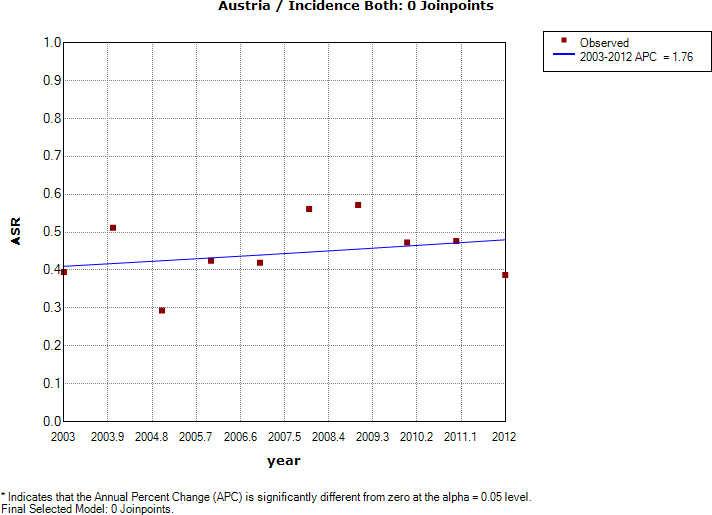

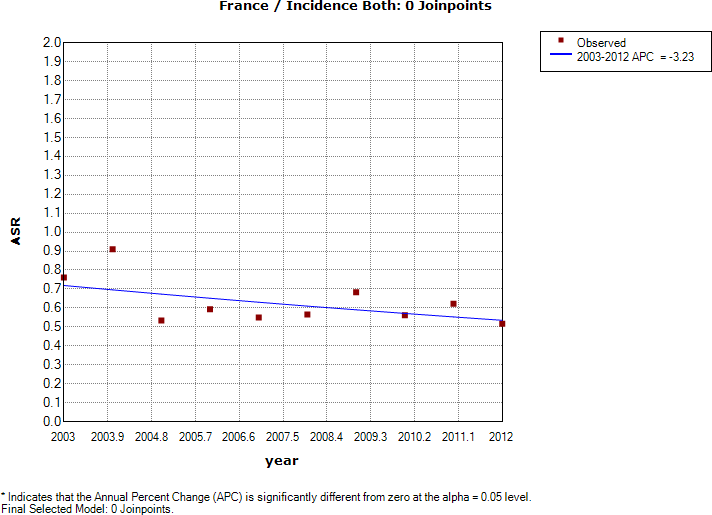

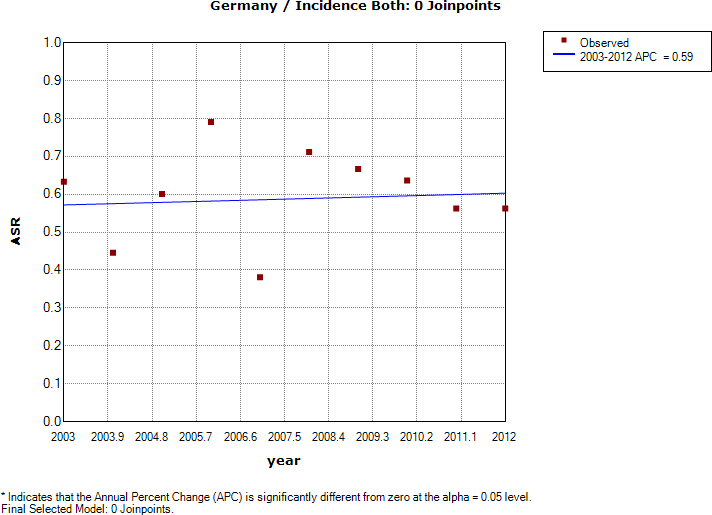

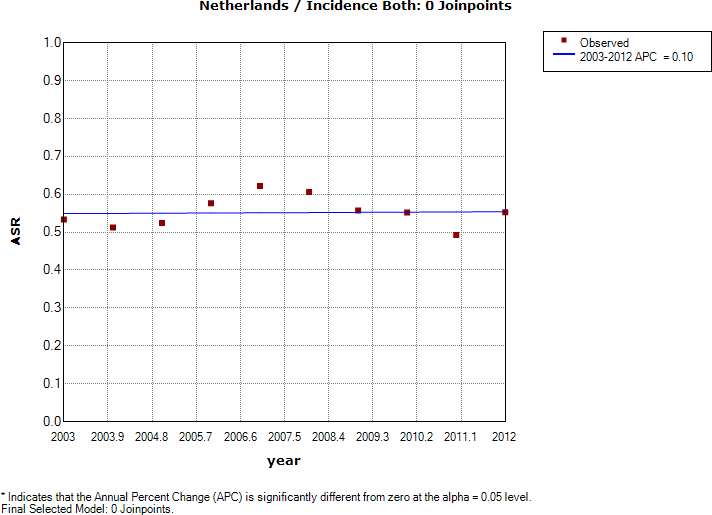

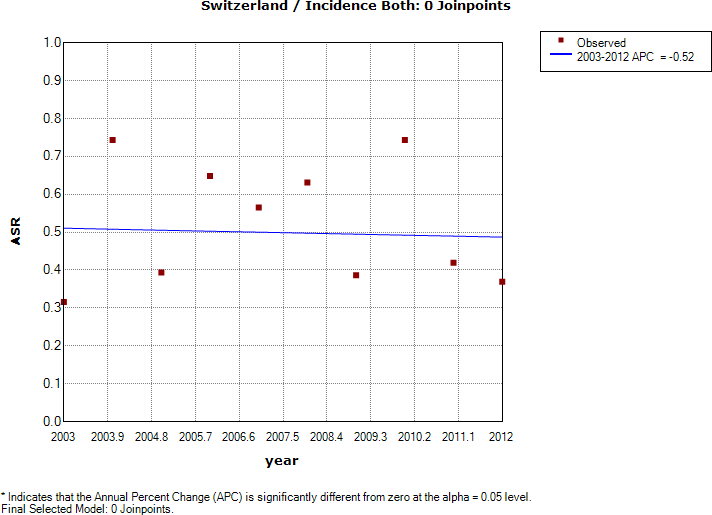


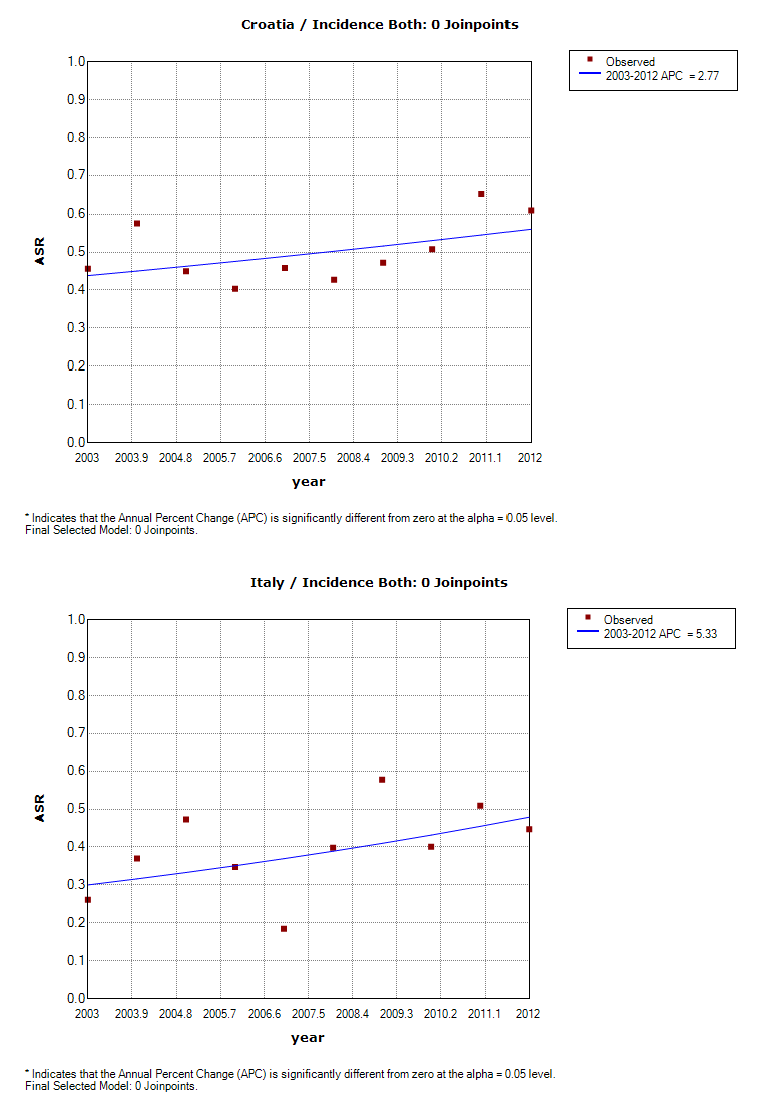

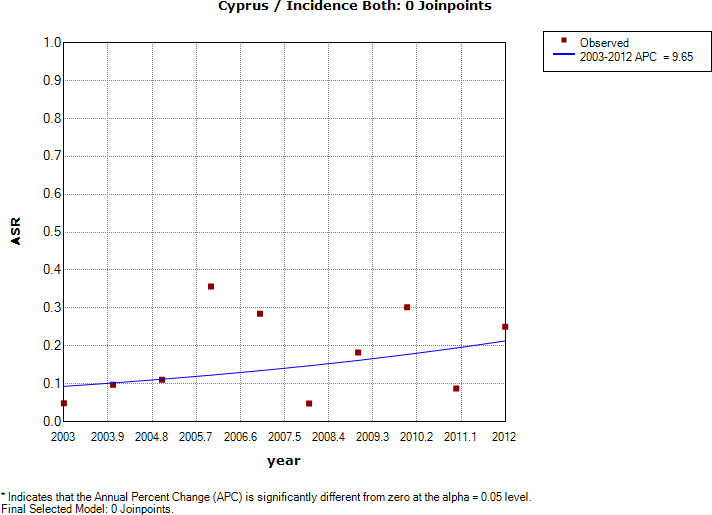

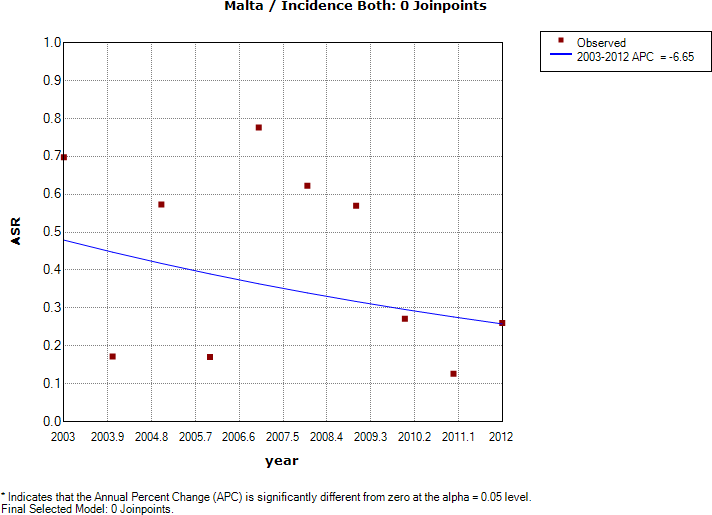
**Southern Europe**


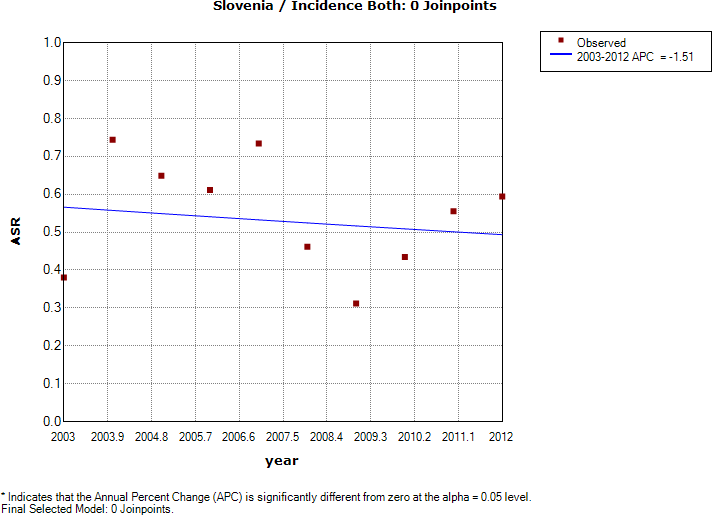

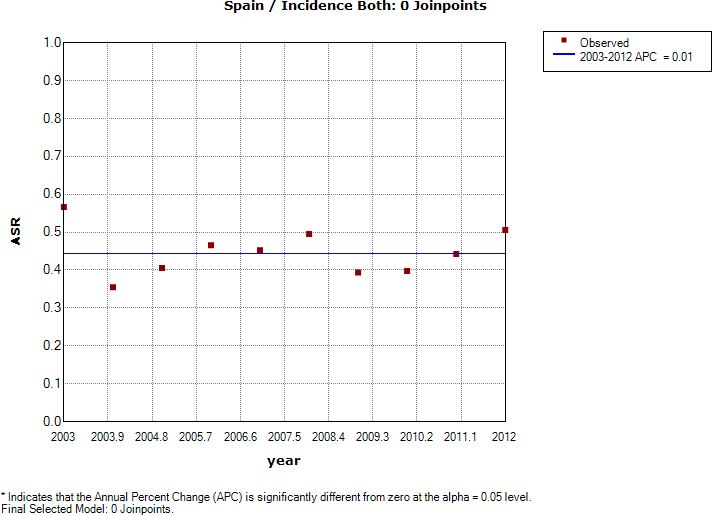


| **Eastern Europe** 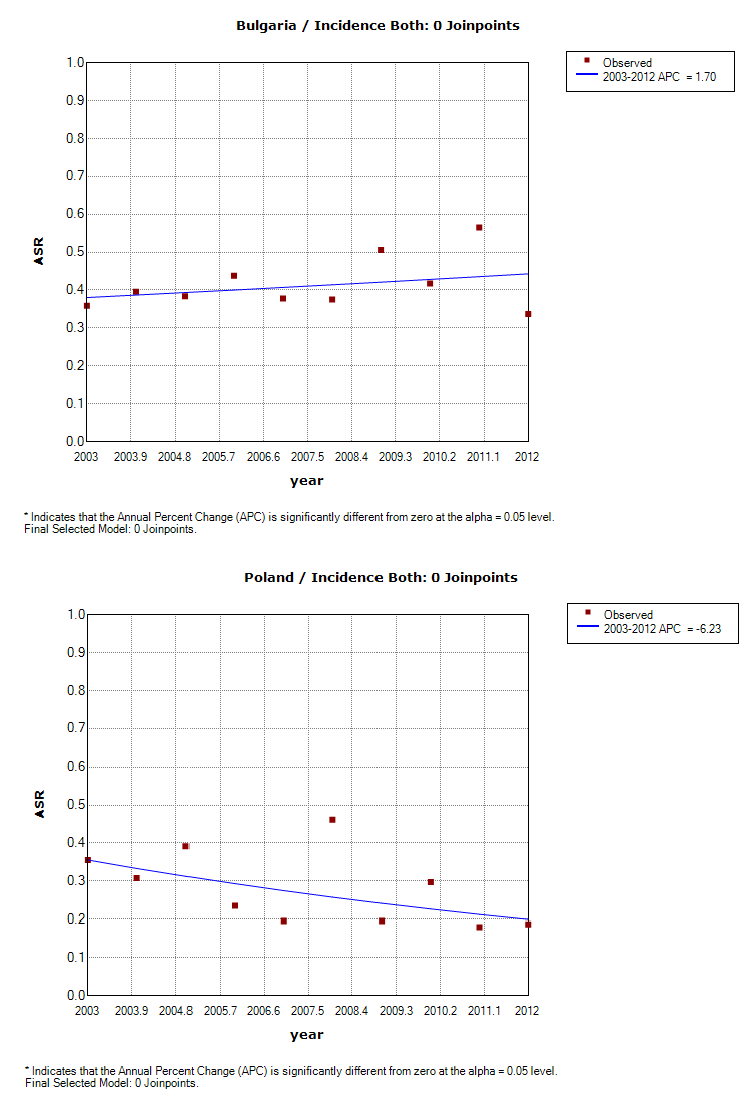 | |
| --- | --- |
|  | 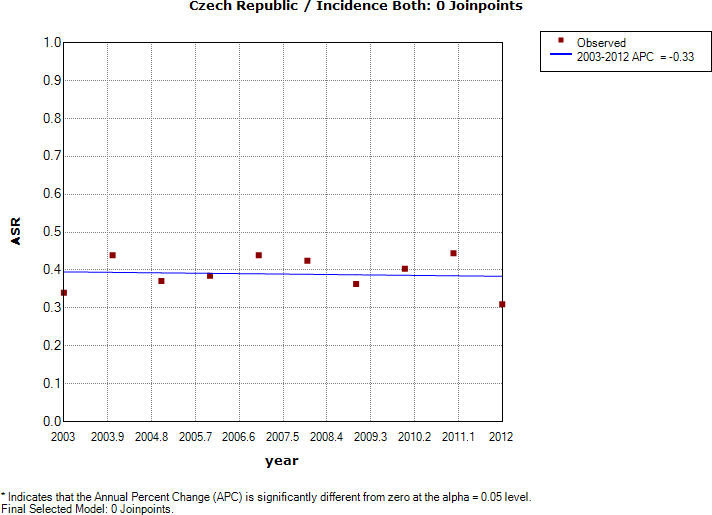 |
|  |  |
| **Africa** | |
| 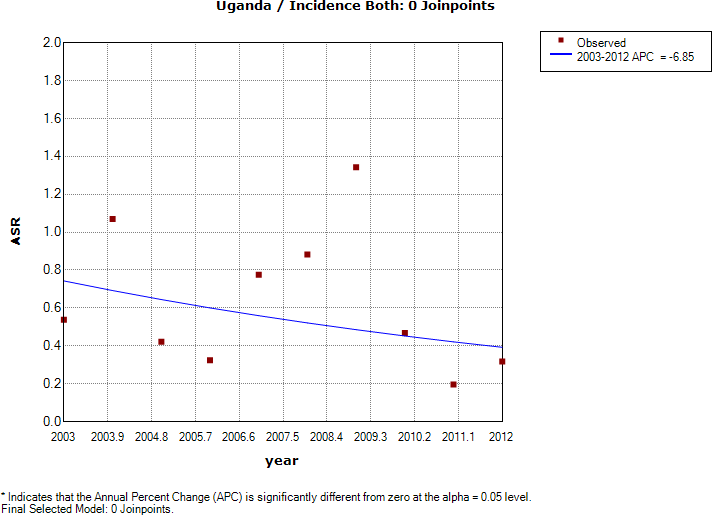 |  |

1. Young

| **Asia** | |
| --- | --- |
|  |  |
|  |  |
|  |  |

|  |  |
| --- | --- |
|  |  |
| **Oceania** | |
|  |  |

| **Northern America** | |
| --- | --- |
|  |  |
| **Southern America** | |
|  |  |
|  |  |
|  |  |

**Northern Europe**

| **Western Europe** | |
| --- | --- |
|  |  |
|  |  |
|  |  |

| **Southern Europe** | |
| --- | --- |
|  |  |
|  |  |
|  |  |

| **Eastern Europe** | |
| --- | --- |
|  |  |
|  |  |
| **Africa** | |
|  |  |

1. Old

| **Asia** | |
| --- | --- |
|  |  |
|  |  |
|  |  |

|  |  |
| --- | --- |
|  |  |
| **Oceania** | |
|  |  |

| **Northern America** | |
| --- | --- |
|  |  |
| **Southern America** | |
|  |  |
|  |  |
|  |  |

**Northern Europe**

**Western Europe**

**Southern Europe**

| **Eastern Europe** | |
| --- | --- |
|  |  |
|  |  |
| **Africa** | |
|  |  |
